# Widespread Pesticide Distribution in the European Atmosphere Questions their Degradability in Air

**DOI:** 10.1021/acs.est.3c08488

**Published:** 2024-02-07

**Authors:** Ludovic Mayer, Céline Degrendele, Petr Šenk, Jiři Kohoutek, Petra Přibylová, Petr Kukučka, Lisa Melymuk, Amandine Durand, Sylvain Ravier, Andres Alastuey, Alex R. Baker, Urs Baltensperger, Kathrin Baumann-Stanzer, Tobias Biermann, Pernilla Bohlin-Nizzetto, Darius Ceburnis, Sébastien Conil, Cédric Couret, Anna Degórska, Evangelia Diapouli, Sabine Eckhardt, Konstantinos Eleftheriadis, Grant L. Forster, Korbinian Freier, François Gheusi, Maria I. Gini, Heidi Hellén, Stephan Henne, Hartmut Herrmann, Adéla Holubová Šmejkalová, Urmas Hõrrak, Christoph Hüglin, Heikki Junninen, Adam Kristensson, Laurent Langrene, Janne Levula, Marie Lothon, Elke Ludewig, Ulla Makkonen, Jana Matejovičová, Nikolaos Mihalopoulos, Veronika Mináriková, Wolfgang Moche, Steffen M. Noe, Noemí Pérez, Tuukka Petäjä, Véronique Pont, Laurent Poulain, Etienne Quivet, Gabriela Ratz, Till Rehm, Stefan Reimann, Ivan Simmons, Jeroen E. Sonke, Mar Sorribas, Ronald Spoor, Daan P. J. Swart, Vasiliki Vasilatou, Henri Wortham, Margarita Yela, Pavlos Zarmpas, Claudia Zellweger Fäsi, Kjetil Tørseth, Paolo Laj, Jana Klánová, Gerhard Lammel

**Affiliations:** † Faculty of Science, RECETOX, Masaryk University, Brno 602 00, Czech Republic; ‡ Laboratory of Chemistry and Environment (LCE), CNRS, Aix-Marseille University, Marseille 13003, France; § Spanish Research Council (CSIC), Institute of Environmental Assessment and Water Research (IDAEA), Barcelona 08034, Spain; ∥ Centre for Ocean and Atmospheric Sciences, University of East Anglia, Norwich NR4 7TJ, United Kingdom; ⊥ Laboratory of Atmospheric Chemistry, Paul Scherrer Institute, Villigen 5232, Switzerland; # GeoSphere Austria, Wien 1190, Austria; ∇ Centre for Environmental and Climate Research, Lund University, Lund 223 62, Sweden; ○ Norwegian Institute for Air Research (NILU), Kjeller 2007, Norway; ◆ School of Natural Sciences and Centre for Climate and Air Pollution Studies, Ryan Institute, University of Galway, Galway H91 CF50, Ireland; ¶ DRD/GES Observatoire Pérenne de l’Environnement, ANDRA, Bure 55290, France; †† German Environment Agency (UBA), Zugspitze 82475 Germany; ‡‡ Institute of Environmental Protection, National Research Institute, Warsaw 02-170, Poland; §§ National Centre of Scientific Research “Demokritos”, Institute of Nuclear Radiological Science Technology, Energy and Safety, ENRACT, Agia Paraskevi 15310, Greece; ∥∥ National Centre for Atmospheric Sciences, University of East Anglia, Norwich NR4 7TJ, United Kingdom; ⊥⊥ Bavarian Environment Agency, Augsburg 86179, Germany; ## Laboratoire d’Aérologie, CNRS/IRD, University of Toulouse, Toulouse 31400, France; ∇∇ Finnish Meteorological Institute, Helsinki 00560, Finland; ○○ Swiss Federal Laboratories for Materials Science and Technology (Empa), Dübendorf 8600, Switzerland; ◆◆ Atmospheric Chemistry Department, Leibniz Institute for Tropospheric Research (TROPOS), Leipzig 04318, Germany; ¶¶ National Atmospheric Observatory Košetice, KošeticeCzech Hydrometeorological Institute, Košetice 395 01, Czech Republic; ††† Institute of Physics, University of Tartu, Tartu 50411, Estonia; ‡‡‡ Department of Physics, Lund University, Lund 223 63, Sweden; §§§ Institute for Atmospheric and Earth System Research (INAR), University of Helsinki, Helsinki 00100, Finland; ∥∥∥ Slovak Hydrometeorological Institute, Bratislava 833 15, Slovakia; ⊥⊥⊥ Department of Chemistry, University of Crete, Heraklion 715 00, Greece; ### Environment Agency Austria, Wien 1090, Austria; ∇∇∇ Institute of Forestry and Engineering, Estonian University of Life Sciences, Tartu 51014, Estonia; ○○○ Environmental Research Station Schneefernerhaus (UFS), Zugspitze 82475, Germany; ◆◆◆ UK Centre for Ecology and Hydrology, Penicuik EH260QB; United Kingdom; ¶¶¶ Géosciences Environnement Toulouse, CNRS/IRD, University of Toulouse, Toulouse 31400, France; †††† Atmospheric Sounding Station El Arenosillo, National Institute for Aerospace Technology (INTA), Huelva 21130, Spain; ‡‡‡‡ National Institute for Public Health and the Environment (RIVM), Bilthoven 3721, MA, the Netherlands; §§§§ Institut des Géoscience de l’Environnement, University Grenoble Alpes, Grenoble 38058, France; ∥∥∥∥ Multiphase Chemistry Department, Max Planck Institute for Chemistry, Mainz 55128, Germany

**Keywords:** pesticides, atmosphere, transport, degradation, risk assessment

## Abstract

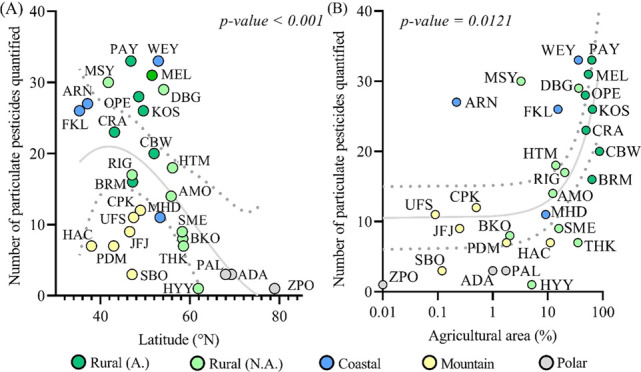

Risk assessment of pesticide impacts on remote ecosystems makes use of model-estimated degradation in air. Recent studies suggest these degradation rates to be overestimated, questioning current pesticide regulation. Here, we investigated the concentrations of 76 pesticides in Europe at 29 rural, coastal, mountain, and polar sites during the agricultural application season. Overall, 58 pesticides were observed in the European atmosphere. Low spatial variation of 7 pesticides suggests continental-scale atmospheric dispersal. Based on concentrations in free tropospheric air and at Arctic sites, 22 pesticides were identified to be prone to long-range atmospheric transport, which included 15 substances approved for agricultural use in Europe and 7 banned ones. Comparison between concentrations at remote sites and those found at pesticide source areas suggests long atmospheric lifetimes of atrazine, cyprodinil, spiroxamine, tebuconazole, terbuthylazine, and thiacloprid. In general, our findings suggest that atmospheric transport and persistence of pesticides have been underestimated and that their risk assessment needs to be improved.

## Abstract 2

Atmospheric pesticide presence and levels in remote areas mirror those in continental Europe, calling into question the current risk assessment assumptions related to degradation in air.

## Introduction

Pesticides are synthetic chemicals used for their toxic effects.^1^ Their agricultural use has significantly increased globally from 2.4 million tons in 1990 to 4.1 million tons in 2020.^2,3^ Chemicals authorized for pesticidal use vary widely in their chemical structures and physicochemical properties.^4,5^ Since the 1960s, growing environmental and health concerns have led to usage restrictions of the previously dominant organochlorine pesticides, to their substitution by more biodegradable ones, and, where possible, to less intensive application.^6^ Because of the toxicity of pesticides and their metabolites to nontarget organisms, environmental exposure to pesticides is a concern.^7,8^ Upon bioaccumulation and biomagnification, lipophilic pesticides (with an octanol–water partition coefficient *K*_OW_ of > 10^5^) may reach effect levels in top predators and humans.^9−11^

Most pesticides are semivolatile organic compounds (SVOCs).^10^ They enter the atmosphere upon application via direct emission from spray drift, through wind erosion of soil particles containing pesticides, and via volatilization from soil, vegetation, or water surfaces.^3,12−14^ Currently used pesticides have been found in rural air^15−17^ and ecosystems^18,19^ but have also been observed far from the sources, at high mountains,^20^ in the marine boundary layer,^21,22^ and in the Arctic.^10,23,24^ Such observations have been sporadic because unlike organochlorine pesticides, these pesticides are rarely included in air-monitoring programs (in Europe apart from France and Sweden^25^).

Evidence for long-range transport to remote areas is one of the criteria for a chemical to be considered a persistent organic pollutant (POP).^26,27^ Unlike organochlorine pesticides (e.g., DDT, lindane) which are classified as POPs, it has been largely considered that the new generation of pesticides, currently in use globally, are not prone to long-range atmospheric transport (LRAT) due to their short atmospheric half-lives (i.e., <2 days).^27^ The atmospheric lifetime of SVOCs is determined by gas/particle partitioning and their reactivity in the gaseous and particulate phases and, if degradation is resisted in Earth surface compartments, might be enhanced by multiple cycles of deposition and revolatilization (grasshopper effect).^28,29^ However, gas/particle partitioning of currently used pesticides is incompletely understood and experimental data for reactions with atmospheric oxidants are available for only few substances.^30−35^ Without such data, degradability in air is often assessed based on model-estimated reactivity with the hydroxyl (OH) radical in the homogeneous gas phase.^36^ However, recent findings have shown the presence of pesticides with theoretical persistence in air below the LRAT potential threshold also in remote areas.^22−24^ This questions the accuracy of the pesticide risk assessment in Europe to protect the atmospheric environment and remote ecosystems, which considers only the atmospheric half-life, for which only the modeled reactivity with the OH radical in the gas phase is considered.

Here, taking advantage of air-monitoring infrastructures, we present the continental-scale distributions of 76 pesticides in the atmosphere over Europe. To this end, 77 particulate and 17 gas-phase samples were collected during the main pesticide application period in spring 2020 at 29 rural, coastal, mountain, and polar sites across Europe and the European Arctic (Figure S1 and Table S1).

## Materials and Methods

### Sampling Sites

The contributing 29 sampling sites (Table S1) are observational platforms of the Aerosol, Clouds and Trace Gases Research Infrastructure (ACTRIS, www.actris.eu/) and/or the Co-operative Programme for Monitoring and Evaluation of the Long-range Transmission of Air Pollutants in Europe (EMEP, www.emep.int/) and have long-term expertise with atmospheric aerosol particle and trace gas sampling. These 29 sampling sites are located in 17 different European countries and in the European Arctic (Figure S1) and were classified as rural (*n* = 16), coastal (*n* = 4), mountain (*n* = 6), and polar (*n* = 3) based on their geographical characteristics and/or land use analysis. Indeed, the mountain and polar sites were defined based on their geographical characteristics with, respectively, an altitude of >2000 m a.s.l. and a latitude of >67°N. In the second step, the type of land use surrounding each sampling site (10 km radius) was characterized using the CORINE Land Cover 2018 for all sites except the Zeppelin Observatory for which the Global Land Cover 2000 was used (Table S2).^37^ To this end, the many categories available from these databases were grouped into more relevant ones considering the aim of this study (Table S3). The coastal sites were defined as those having >35% of their surrounding areas (10 km radius) as water bodies, while for rural sites, it was >60% of agricultural land, forest and shrub, and/or herbaceous vegetation associations. In addition, the rural sites were subcategorized as agricultural-adjacent (A.) or nonagricultural-adjacent (N.A.) if their share of agricultural land in their surrounding area was above or below 45%, respectively.

### Sampling

Sampling took place simultaneously at all 29 sites in the main pesticide application season in spring 2020 during three 48 h sampling periods (namely, 28–30/04, 12–14/05, and 26–28/05). Sampling was performed with active air samplers (low or high volume, based on on-site availability, Table S1). Due to Covid-19 epidemic-related restrictions in some countries, only 22 sites collected samples during all three sampling periods, while 4 and 3 sites collected samples during 2 and 1 sampling period, respectively (Table S1).

All sites collected the particulate phase on quartz fiber filters (QFF, QM-A, Whatman, U.K.), preferentially (but not always) with a PM_10_ inlet, as CUPs have previously been found in both the fine and coarse particles.^15^ In addition, six sites (ADA, BKO, KOS, SBO, UFS, and ZPO) sampled also the gaseous phase on a sandwich sorbent (i.e., PUF/XAD2/PUF sandwich), consisting of a polyurethane foam plug (PUF, Molitan a.s., Czech Republic, density 0.030 g cm^–3^, 5 cm depth, diameter of 5.5/11 cm for the low/high-volume air sampler), a layer of XAD resin (Supelpak-2, Supelco), and another PUF plug, separated by cotton wool. This sandwich configuration has been shown to be the most efficient for the collection of gaseous pesticides.^38^ Prior to sampling, PUFs and XAD2 were precleaned via Soxhlet extraction with acetone for 8 h, followed by 8 h of extraction in methanol. All sampling media were provided by RECETOX and shipped to the sites. In total, 77 samples and 35 field blank samples were collected (i.e., 77 QFFs and 17 PUF/XAD2/PUF sandwiches) and kept in the freezer at −18 °C until extraction.

### Sample Preparation, Analysis, and QAQC

All samples underwent spiking with labeled standards (Table S4) before extraction. The extraction process involved using an automatic extractor (Büchi Extraction System, B-811, Switzerland) with 5 mM ammonium acetate in methanol. The extraction process consisted of 1 h of warm Soxhlet, followed by 1 h of solvent rinsing, and a concentration step to 1 mL using nitrogen. After centrifugation for 10 min (12,000 G, Z-36 HK, Hermle Labortechnik, Germany) within polypropylene tubes (Corning Costar Spin-X), the extracts were filtered (cellulose acetate membrane and 0.22 μm pore size) and further concentrated to 0.5 mL under nitrogen.

Postextraction, the samples were divided into three 100 μL aliquots, each undergoing a different analysis. The three analyses allowed the quantification of 76 pesticides (35 herbicides, 22 insecticides, and 19 fungicides) (Table S5). Among these pesticides, 40 are approved for agricultural use in Europe, 22 are among the most globally used pesticides, 34 are characterized as priority active ingredients to be monitored in France, 13 are highly hazardous pesticides, and 25 are high-risk pesticides.

To ensure quality assurance, field blanks were analyzed alongside air samples collected. Blank levels of most individual analytes were generally below or low (Tables S6 and S7). Procedural recoveries were assessed by spiking sampling media with native standards and their corresponding isotopic-labeled standards, followed by processing as per sample. Most procedural recoveries fell within the range of 60–120% with standard deviations below 20%, except for a few exceptions.

Detailed information about the procedures is provided in the Supporting Information.

### Air Mass Origin

The Lagrangian particle dispersion model FLEXPART^39^ was used to identify the potential source regions by calculating the residence times in the surface layer of air sampled.

The meteorological data used (0.5 and 1° and 3 h horizontal and temporal resolutions, 137 vertical levels) were obtained from the European Centre for Medium-Range Weather Forecast (www.ecmwf.int, last access: 21/06/2022). For each simulation, 100,000 particles were continuously released from the sites at the ground level for the polar sites and at altitudes ranging 190–210 m agl for mountain sites and were followed for 30 and 10 days backward in time at polar sites and mountain sites, respectively (Figures S2–S5).

### Advection to Mountain Sites

For the six high-mountain sites, the advection was characterized using various combinations of on-site tracer measurements and meteorological data and modeling. For all mountain sites, the conclusions on the planetary boundary layer (PBL) influence on the sampled air were based on the site-specific experiences (Table S8).

At CPK, regional chemistry-transport modeling data (ALADIN model, 2 km × 2 km horizontal resolution)^40^ were used to judge whether the sampled air was within the free troposphere (FT) or within the PBL. The CPK site was within the FT during most of the sampling time. However, due to short-term (<1 h) liftings of the inversion (which occurred 2, 10, and 2 times during the sampling periods 1, 2, and 3, respectively), PBL air was mixed into all three air samples.

The model terrain is ca. 500 m below the true altitude. Moreover, due to resolution limitations of the model, in particular, in complex terrain, upslope movement of air from valleys will be systematically underestimated. For HAC, the on-site gaseous (i.e., CO, CO_2_) and aerosol tracer measurements, meteorological parameters, and planetary boundary layer height (obtained from ECMWF) were used.^41−43^ Air collected during period 2 represented almost exclusively the FT. It was heavily affected by Sahara dust long-range transport. Sampling period 3 was characterized by relatively low and stable concentrations of specific tracers (i.e., aerosol absorption and particle number concentration), but the station was mostly in clouds, indicating conditions characteristic of the interface between FT and PBL. PBL influence on air collected cannot be excluded. No sample was collected during period 1. For JFJ, on-site Rn measurements^44^ and data from a ceilometer, obtained at the foot of the site (measured at Kleine Scheidegg, altitude difference of 1510 m; 6 km direct distance from JFJ),^45^ were used to judge free tropospheric vs boundary layer air. JFJ was above the planetary boundary layer during short periods in all three sampling periods. The samples represent mixed FT and PBL air. At PDM, the analysis of ^222^Rn measurements (α-detection) suggested mixed FT and PBL air during sampling period 3. No samples were collected during periods 1 and 2. For SBO, data from a ceilometer (Vaisala CL51)^46^ obtained at the foot of the site (measured at Kolm-Saigurn, altitude difference of 1466 m; 5 km direct distance from SBO) were used to judge on FT vs PBL air. Heights for measuring periods with signals under the detection limit, which occurred during nighttime, were interpolated. The derived heights of the PBL were below SBO during all three sampling periods. No sample was collected during period 1. At UFS, the ^222^Rn measurements (α-detection)^47^ suggested FT air with some PBL influence during the sampling periods 1 and 2 and almost exclusively FT air during sampling period 3. The attribution is supported by on-site measurements of humidity and other meteorological parameters.^48^ In addition, advection and possible collection of FT air were investigated for two other elevated sites, i.e., RIG (Switzerland, 1031 m a.s.l.) and ZPO (Svalbard, 474 m a.s.l.). In situ and ceilometer data,^49^ respectively, besides others, indicated that at both sites, the air collected during the three sampling periods was PBL air or PBL with little FT air mixed in.

### Data Analysis

All the statistical analysis was performed using software GraphPad Prism (v9.0.0). For these analyses, when the concentration of a compound was lower than that of iLOD, iLOQ, or LOQb, these values were not taken into account. Substitutions of values below LOQ by LOQ/2 were used to determine the relative standard deviation (Table 1).

**Table 1:** Spatial Homogeneity of Distributions: Concentration Range (Expressed as log(*c*_max_/*c*_min_)), Relative Standard Deviation (RSD; %) at All Sites and Comparison of Mean Particulate Concentrations at Remote Sites, *c*_rs_ (Polar + Free Tropospheric Mountain Sites) and Other Sites, *c*_os_ (Coastal + Nonfree Tropospheric Mountain + Rural Sites)^a^^,^^b^

	*c*_rs_ (pg m^–3^)	*c*_os_ (pg m^–3^)	log(*c*_os_/*c*_rs_)	log(*c*_max_/*c*_min_)	RSD (%)
2,4-D	<LOQ	4.46	N.A.^b^	2.1	159
atrazine	0.11	0.30	0.4	1.6	107
cyprodinil	26.5	215	0.9	5.7	602
fenpropidin	7.30	127	1.2	4.5	548
fenpropimorph	2.37	46.1	1.3	3.6	199
metazachlor	<LOQ	2.59	N.A.^b^	3.2	263
S-metolachlor	5.16	81.7	1.2	3.2	147
spiroxamine	11.9	78.3	0.8	4.0	378
tebuconazole	0.95	10.3	1.0	3.4	213
terbuthylazine	54.3	53.5	0.0	3.4	291
thiacloprid	0.24	1.14	0.7	1.7	103

a Values < LOQ not included. Substances with a quantification frequency higher than 50% only.

b N.A. = not applicable.

Detailed information about the procedures is provided in the Supporting Information.

## Results and Discussion

### European Distribution of Atmospheric Pesticides

**Figure 1:**
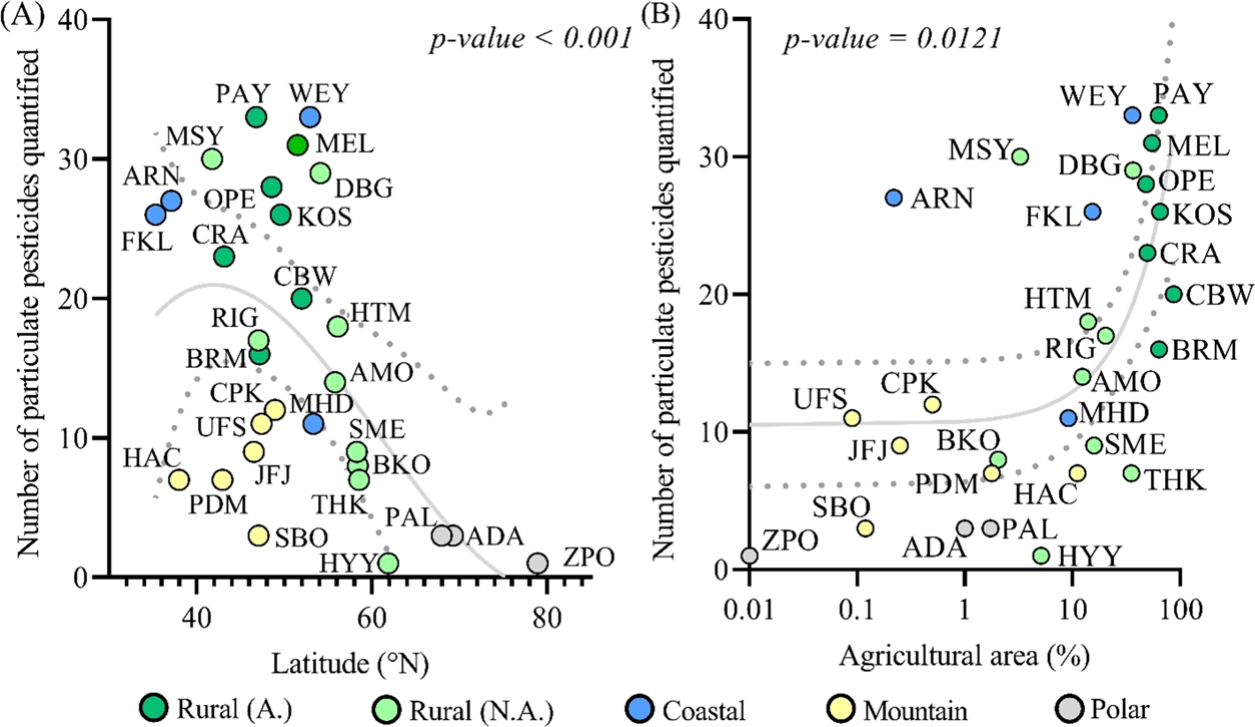
Number of pesticides quantified in the particulate phase: (A) latitudinal distribution (third-order polynomial regression) and (B) related to agriculture (area fraction within 10 km, in %). On each figure, the gray line represents the regression and the dotted lines represent the 95% confidence interval.

Out of the 76 pesticides targeted, 58 were found in the atmosphere, including the European Arctic. In the atmospheric particulate phase, 51, 38, 24, and 6 pesticides were found at rural, coastal, mountain, and polar sites, respectively (Figures S6–S8). Overall, the number of particulate pesticides decreases with latitude and increases with the proximity to agricultural fields (Figure 1). Among these 58 pesticides present in European air, about 50% were rarely found (1–5 sites), while around 20% were quantified in more than half of the sites investigated (Table S9).

**Figure 2:**
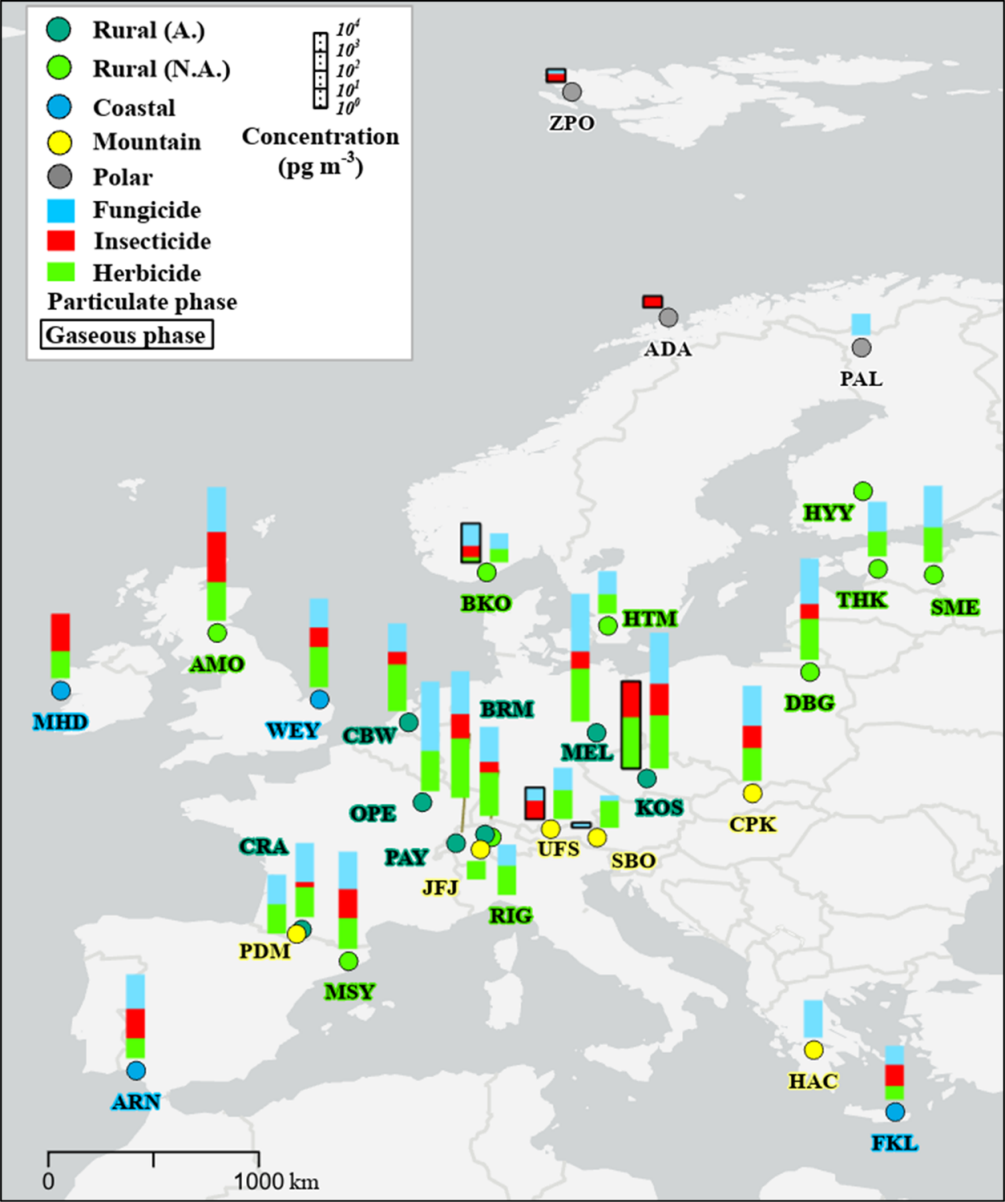
Pesticide mean concentrations quantified at each site in the particulate (all sites) and gaseous phases (6 sites).

The concentrations of pesticides quantified on aerosol particles were minimal at ZPO (Svalbard) with 24.5 fg m^–3^ on average and ranged from 0.14 to 3.9 ng m^–3^ at agricultural sites (CRA and OPE sites, respectively) (Figure 2). On an individual substance point of view, cyprodinil, fenpropidin, prosulfocarb, and spiroxamine were the pesticides with the highest concentrations (i.e., up to 7.51, 3.53, 1.71, and 1.82 ng m^–3^, respectively), while for all other pesticides, their concentrations were mostly below 1 ng m^–3^.

8 out of 11 pesticides exhibiting a quantification frequency exceeding 50% (Table S9) (i.e., cyprodinil, fenpropidin, fenpropimorph, metazachlor, S-metolachlor, spiroxamine, tebuconazole, and terbuthylazine) had their particulate concentrations spanning 3 to 6 orders of magnitude (log(*c*_max_/*c*_min_) = 3–6), while the other three (i.e., 2,4-D, atrazine, and thiacloprid) exhibited a more uniform distribution across the continent (log(*c*_max_/*c*_min_) ≈ 2 and RSD < 200%) (Table 1 and Figures S9 and S10). Moreover, for atrazine and thiacloprid, the concentration gradient from the source areas was particularly small: The average concentrations at remote sites (free tropospheric mountain and Arctic) were within an order of magnitude of the average concentrations at other sites (rural + coastal + mountain) (log(*c*_os_/*c*_rs_) < 1; Table 1). The same was found for cyprodinil, spiroxamine, tebuconazole, and terbuthylazine. Such uniform distributions suggest that the atmospheric lifetime of the compounds is similarly long or its source distribution is similarly wide as that of atrazine. Atrazine, an herbicide banned since 2004,^50^ continues to be present in European air, likely a consequence of its persistence and long-range transport from regions where it is still used.^3,51−53^ Because of its persistence, atrazine keeps cycling in air, even 25 years after its ban in Europe. It is re-emitted from secondary sources, but primary sources outside Europe may also contribute.^54^

Among these frequently observed pesticides, 8 are approved for agricultural use in Europe, except for atrazine, fenpropimorph, and thiacloprid (Tables S5). Fenpropimorph is a fungicide banned shortly before our sampling campaign but remained authorized for use as a biocide until 2021.^55^ Thiacloprid, banned for agricultural use in May 2020, is another exception. Among the less frequently found pesticides, acetochlor, carbaryl, and simazine, their European approvals had lapsed more than 9 years before our sampling. Interestingly, concentrations of these substances at remote sites did not significantly differ from those at rural sites by more than an order of magnitude (Table 1), indicating their persistence and long-range transport from regions where they are still in use, such as North America and Africa.

By hierarchical cluster analysis, we find a high degree of similarity in particulate pesticide substance patterns between sites far from application, i.e., polar and mountain sites, particularly evident during the third sampling period (Figures S11–S13).

### Long-Range Atmospheric Transport (LRAT) of Pesticides

The substances prone to long-range atmospheric transport were identified using the samples collected at the polar sites as well as those collected at high mountain sites from free tropospheric air (see the Supporting Information for the determination of free tropospheric conditions at each site). Indeed, in the free troposphere, zonal and meridional transport is more efficient due to higher wind speeds and longer depositional lifetime.^56^

**Figure 3:**
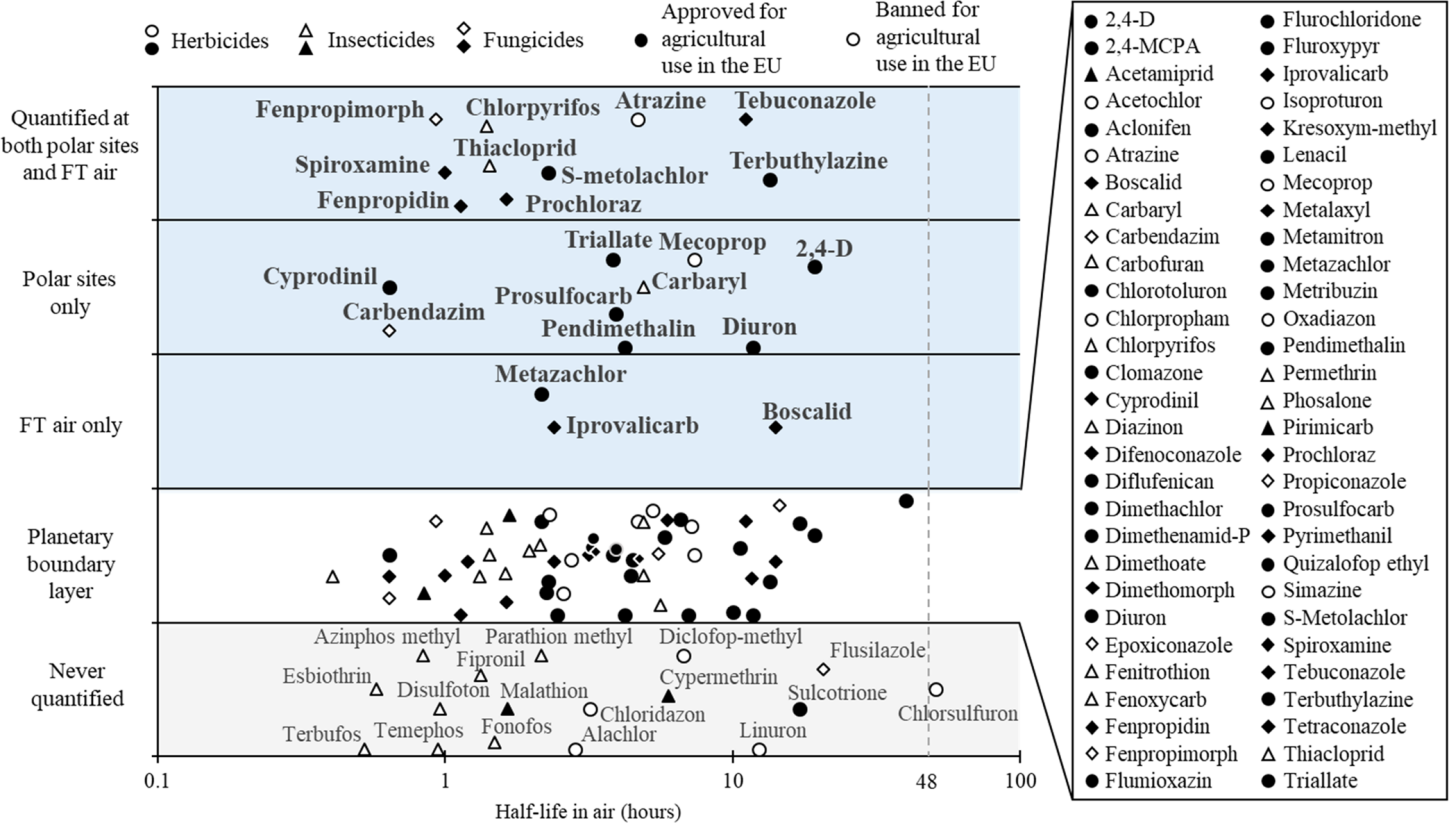
Pesticides identified as prone to long-range atmospheric transport (blue area, FT = free tropospheric), pesticides quantified in the planetary boundary layer (white area), and pesticides never observed (gray area) ordered along model estimate of half-life in air.^36^ Not pictured is one pesticide never observed, i.e., fluazinam, with a model-estimated half-life of 1956 h. Annual mean OH concentration of 7.5 × 10^5^ cm^−3^ used for calculation of half-life in air.

In the polar atmosphere, 19 pesticides were quantified, including 15 for which this is the first evidence of their potential to reach the Arctic. Out of these 19 pesticides, 12 were approved for agricultural use in Europe in 2020. In particular, most of these pesticides were quantified in the gas phase at the ZPO site, Svalbard (78.9°N). 13 pesticides were found in the four samples at mountain sites that were confirmed to have been collected exclusively in free tropospheric air (Table S8). This included 10 approved for agricultural use and 10 that were also found at the polar sites. Therefore, by combining the results from the polar and free tropospheric air samples, 22 pesticides were identified to be prone to LRAT (blue area in Figure 3), which included 15 approved for agricultural use in Europe.

Source regions, based on 30-day footprint analyses by the FLEXPART model, for the air collected from 28 to 30/04/2020 and 26–28/05/2020 at ZPO, Svalbard, are located in the North Atlantic and the inner Arctic (Figure S2) and contained the lowest number and concentrations of pesticides. Higher levels were quantified in the air collected from 12 to 14/05/2020 at ZPO, which originated from North America and the northernmost agricultural regions of Russia (Figure S2). Similar behavior has also been found for the two other polar sites, where higher numbers of pesticides were encountered in the samples, where air masses were influenced by Scandinavia and the British Isles (Figures S3 and S4). The origins of free tropospheric air collected at mountain sites were all from continental Europe (Figure S5).

Previous observations of more than 20 currently used pesticides in the polar regions in recent years have highlighted the LRAT potential of currently used pesticides^10,22−24^ (Table S10). However, these studies have mainly focused on a limited number of currently used pesticides. In this study, 19 pesticides were found to be associated with LRAT at the polar sites. Out of these, four substances, atrazine, chlorpyrifos, pendimethalin, and triallate, had previously been reported in polar regions, whereas we are establishing the first evidence of LRAT for 15 pesticides that have previously not been encountered in these regions (Figure 3). Three of these substances, atrazine, chlorpyrifos, and diuron, are water policy-prioritized in the EU. The potential risk for the environment and human health is also evidenced by the fact that among the pesticides identified as prone to LRAT, 2 are carcinogens, 6 are genotoxic, 10 are endocrine disruptors, and 9 are reprotoxic.^10,57^ In addition, the 22 long-range transported pesticides belong to 16 different chemical classes^57^ and their physicochemical properties range widely, i.e., saturation vapor pressure ranging from 9.2 × 10^–9^ to 0.67 Pa with log *K*_OW_ from 1.55 to 6.52 (Table S11), with chlorpyrifos, fenpropidin, fenpropimorph, pendimethalin, S-metolachlor, and spiroxamine potentially (log*K*_ow_ > 5) known to be bioaccumulative.^19^

There was a considerable shift of the pesticides’ gas/particle partitioning from the continent to the Arctic, with a higher gaseous fraction for 14 out of 15 pesticides observed at the polar sites than at midlatitude sites. This trend was most prominent for terbuthylazine, which was on average 99% in the particulate phase at rural sites but >98% in the gaseous phase at polar sites. The exception was triallate, which at one polar site was quantified in the particulate phase only (>87%, Table S12). Our observations suggest that pesticides which are predominantly partitioning to the particulate phase in aerosols over Europe tend to be found in the gaseous phase in the Arctic (Figure 2). This pattern is consistent with previous studies, with pesticides such as 2,6-dichlorobenzonitrile, chloroneb, dicofol, nitrapyrin, and triallate observed only in the gas phase of Arctic air^22,24^ (Table S10), while in in this study, pesticides in continental Europe preferentially partitioned to the particulate phase, as suggested by the results from mountains and rural sites where the gas phase was also collected.^15^

A preference for partitioning to the gas phase at polar sites can be explained by low aerosol mass and surface concentrations and the prevalence of hydrophilic particulate matter (PM) components in particular seasalt.^58^ For example, at the central European rural site KOS (Czech Republic), the concentration of PM_10_ was 12.8 μg m^–3^ on average during the study, while 4.3 μg m^–3^ was measured at the polar site ZPO, Svalbard.^59^ Organic matter is a key constituent influencing the partitioning of chemicals onto PM.^60^ In continental Europe, the fraction of organic matter in PM is generally higher than that in the Arctic. At the rural sites adjacent to agriculture, the particulate organic carbon averaged 1.53 μg m^–3^ (≈12%), whereas at polar sites, it was more than 10-fold lower, 0.11 μg m^–3^ (≈3%) on average. No in-depth analysis of gas/particle partitioning is possible because of the lack of particulate phase chemical composition data for most of the sites. Another possible explanation could be volatilization of CUPs from melting snow, mixed into sample air shortly before collection, i.e., before complete relaxation to phase equilibrium. Volatilization from melting snow is expected for organic substances, which within snowpacks would partition significantly to the pore space,^61^ however, this does not apply for the CUPs, which partitioning apparently shifted to the gas phase (Table S12).

The three polar sites included in this study had similar environmental conditions (temperature, PM concentration) but are distanced from each other by >1000 km and were influenced by different air masses (Figures S1–S5). Still, out of the 19 pesticides encountered at these sites, five were observed at more than one polar site, which provides independent evidence of long-range atmospheric transport. Moreover, we quantified 13 pesticides in free tropospheric air collected at three high mountain sites, indicating that they are prone to LRAT. 11 pesticides identified at those mountain sites were also identified at polar sites, thus providing strong evidence of their LRAT potential.

### Current Limitations Regarding Pesticide Environmental Risk Assessments

Pesticide authorization in Europe presupposes environmental risk assessment with criteria for persistence, bioaccumulation potential, long-range transport potential, and toxicity for soil and water. However, there is no threshold value to consider a pesticide as persistent in the atmosphere.^27^ The only parameter through which the atmosphere is included in the risk assessment procedure is the potential for long-range atmospheric transport. To assess this potential, the atmospheric half-life is used as a proxy. For this, only the reactivity with the OH radical in the gas phase is considered, using global and annual mean OH radical concentrations.^62^ A pesticide exhibiting a half-life higher than 2 days is considered as prone to LRAT.

However, all 22 pesticides identified as prone to LRAT in this study have been estimated to have atmospheric half-lives shorter than 2 days based on the model currently used in the risk assessment^36^ (Table S11). But OH concentrations can be much lower seasonally, at high latitudes, as well as during nighttime or in the polar night.^63^ In these cases, degradation processes are substantially reduced, with correspondingly longer effective atmospheric half-lives and travel distances.^24^ For instance, the atmospheric and total environmental lifetimes of atrazine are almost 1 order of magnitude longer for midlatitude winter than in summer^64^ and the characteristic travel distance (CTD) of chlorpyrifos increases from 30 to 290 km with a 10-fold reduced OH concentration.^52^ CTD is an indicator of a chemical’s long-range transport potential in a generic multicompartment environment under steady-state conditions and is influenced by also lifetime in soil and water.^52,65^ It is defined as the distance from the source region at which the concentration is reduced by 63%.^66^ Note that because of the generic nature of the underlying multimedia model, CTD is not suitable to test substance fate or should not be interpreted in absolute terms (km). CTDs of the targeted pesticides are estimated mostly below 100 km (median is 92 km) and are estimated even somewhat lower with a median of 68 km for the substances identified as prone to LRAT, suggesting that these chemicals are unlikely to reach remote locations. The CTD values or their ranking do not correspond with substances suggested from this or earlier studies to have high long-range transport potential, with only one exception, i.e., thiacloprid (Figure S14 and Table S11).

Moreover, for pesticides transported in the particulate phase, degradation is expected to be slower than in the gas phase due to diffusion limitation in low-viscosity aerosol particles.^33,67^ Experiments on degradation rate coefficients with OH in the particulate phase suggest atmospheric half-lives of weeks (given global mean OH radical concentration).^33,34^ Oxidation is particularly slow in aerosols transported at high altitudes or to high latitudes and expectedly most relevant for moderately polar pesticides such as carbamates, triazines, thiophosphoric acid esters, phenols, and anilines.^34,67^ As confirmed in this study, pesticides tend to have higher particle-bound mass fractions in continental air than in polar air, and their persistence in air is therefore likely underestimated. This aspect is neglected by the current risk assessment practice.

Furthermore, the effective atmospheric lifetime of semivolatiles resisting degradation in soils and surface water can be much longer than the residence time in air based on degradation kinetics because of several cycles of revolatilization and deposition enhancing the LRAT potential (multihopping).^28^

In addition to the lack of information about active ingredients, the influence of application methods, formulants, and adjuvants on CUP emissions is not well known. While aerial application is banned in Europe, spraying of soil and plant,^12^ volatilization,^68,69^ and also pellet application to soil and seed treatment contribute to pesticide emissions.^70^ Formulants and adjuvants are used to improve the effectiveness of application but can modify their effective vapor pressure and atmospheric half-lives.^71^ A recent experimental study suggested that the reactivity of the chlorpyrifos with OH radicals was different in a commercial formulation than that of the substance alone.^35^

This study shows the limits of the risk assessment in place in the regulatory process on the atmospheric fate of pesticides and, in particular, their potential for LRAT, by providing empirical evidence in direct contrast to current model predictions. There is a real need to revise the current methods used for environmentally relevant conditions (different temperature and/or OH concentrations) as well as to obtain more experimental data on atmospheric degradability of pesticides including pesticide formulations and preparations, in addition to data from monitoring studies. Currently, the framework does not consider partitioning into the particulate phase or slowed degradation in soil/water during the multihopping. More realistic modeling is extremely important if we want to ensure that the pesticides authorized for agricultural use in Europe (and elsewhere) do not contaminate the environment and pose health risks hundreds of thousands of kilometers away from the source areas.

## Supporting Information Available

Materials and methods, map of the 29 sites, 30-days footprint sensitivities and 10-day backward trajectories (FLEXPART model), CUPs physico-chemical parameters, detailed site results, gas-particle partitioning, hierarchical cluster analysis, sampling methodology, land-use analysis, internal standards and chemical analyses information, recoveries, information on previous CUPs found in Arctic and high-mountain sites (PDF)
