# Supplementary material for: Widespread Pesticide Distribution in the European Atmosphere Questions their Degradability in Air
Source: Environ Sci Technol. 2024 Feb 7;58(7):3342–52. doi: 10.1021/acs.est.3c08488 (PMC10882970; doi:10.1021/acs.est.3c08488)
Supplement: Supplementary file 1 — es3c08488_si_001.pdf [file es3c08488_si_001.pdf]

Supplementary Materials

Widespread pesticide distribution in European atmosphere questions their degradability in air

Ludovic Mayer[1] †, Céline Degrendele[1,2] †‡, Petr Šenk[1], Jiři Kohoutek[1], Petra Přibylová[1], Petr Kukučka[1], Lisa Melymuk[1], Amandine Durand[2], Sylvain Ravier[2], Andres Alastuey[3], Alex R. Baker[4], Urs Baltensperger[5], Kathrin Baumann-Stanzer[6], Tobias Biermann[7], Pernilla Bohlin-Nizzetto[8], Darius Ceburnis[9], Sébastien Conil[10], Cédric Couret[11], Anna Degórska[12], Evangelia Diapouli[13], Sabine Eckhardt[8], Konstantinos Eleftheriadis[13], Grant L. Forster[4,14], Korbinian Freier[15], François Gheusi[16], Maria I. Gini[13], Heidi Hellén[17], Stephan Henne[18], Hartmut Herrmann[19], Adéla Holubová Šmejkalová[20], Urmas Hõrrak[21], Christoph Hüglin[18], Heikki Junninen[21], Adam Kristensson[22], Laurent Langrene[10], Janne Levula[23], Marie Lothon[16], Elke Ludewig[6], Ulla Makkonen[17], Jana Matejovičová[24], Nikolas Mihalopoulos[25], Veronika Mináriková[24], Wolfgang Moche[26], Steffen M. Noe[27], Noemí Pérez[3], Tuukka Petäjä[23], Véronique Pont[16], Laurent Poulain[19], Etienne Quivet[2], Gabriela Ratz[15], Till Rehm[28], Stefan Reimann[18], Ivan Simmons[29], Jeroen E. Sonke[30], Mar Sorribas[31], Ronald Spoor[32], Daan P.J. Swart[32], Vasiliki Vasilatou[13], Henri Wortham[2], Margarita Yela[31], Pavlos Zarmpas[25], Claudia Zellweger Fägi [18], Kjetil Tørseth[8], Paolo Laj[33,23], Jana Klánová[1] and Gerhard Lammel[1,34*]*

[1]Masaryk University, Faculty of Science, RECETOX, Brno, Czech Republic; [2]Aix-Marseille University, Laboratory of Chemistry and Environment (LCE), CNRS, Marseille, France; [3]Spanish Research Council (CSIC), Institute of Environmental Assessment and Water Research (IDAEA), Barcelona, Spain; [4]University of East Anglia, Centre for Ocean and Atmospheric Sciences, Norwich, United Kingdom; [5]Paul Scherrer Institute, Laboratory of Atmospheric Chemistry, Villigen, Switzerland; [6]GeoSphere Austria, Wien, Austria; [7]Lund University, Centre for Environmental and Climate Research, Lund, Sweden; [8]Norwegian Institute for Air Research (NILU), Kjeller, Norway; [9]School of Natural Sciences and Centre for Climate and Air Pollution Studies, Ryan Institute, University of Galway, Galway, Ireland; [10]ANDRA, DRD/GES Observatoire Pérenne de l'Environnement, Bure, France; [11]German Environment Agency (UBA), Zugspitze, Germany; [12]National Research Institute, Institute of Environmental Protection, Warsaw, Poland; [13]National Centre of Scientific Research Demokritos, Institute of Nuclear Radiological Science Technology, Energy and Safety, Agia Paraskevi, Greece; [14]National Centre for Atmospheric Sciences, University of East Anglia, Norwich, United Kingdom; [15]Bavarian Environment Agency, Augsburg, Germany; [16]University of Toulouse, Laboratoire d'Aérologie, CNRS/IRD, Toulouse, France; [17]Finnish Meteorological Institute, Helsinki, Finland; [18]Swiss Federal Laboratories for Materials Science and Technology (Empa), Dübendorf, Switzerland; [19]Leibniz Institute for Tropospheric Research (TROPOS), Atmospheric Chemistry Department, Leipzig, Germany; [20]Czech Hydrometeorological Institute, National Atmospheric Observatory Košetice, Košetice, Czech Republic; [21]University of Tartu, Institute of Physics, Tartu, Estonia; [22]Lund University, Department of Physics, Lund, Sweden; [23]University of Helsinki, Institute for Atmospheric and Earth System Research (INAR), Helsinki, Finland; [24]Slovak Hydrometeorological Institute, Bratislava, Slovakia; [25]University of Crete, Department of Chemistry, Heraklion, Greece; [26]Environment Agency Austria, Wien, Austria; [27]Estonian University of Life Sciences, Institute of Forestry and Engineering, Tartu, Estonia; [28]Environmental Research Station Schneefernerhaus (UFS), Zugspitze, Germany; [29]UK Centre for Ecology and Hydrology, Penicuik, United Kingdom; [30]University of Toulouse, Géosciences Environnement Toulouse, CNRS/IRD, Toulouse, France; [31]National Institute for Aerospace Technology (INTA), Atmospheric Sounding Station El Arenosillo, Huelva, Spain; [32]National Institute for Public Health and the Environment

(RIVM), Bilthoven, Netherlands; [33]University Grenoble Alpes, Institut des Géoscience de l'Environnement, Grenoble, France; [34]Max Planck Institute for Chemistry, Multiphase Chemistry Department, Mainz, Germany

† these authors contributed equally to this work; ‡ now unemployed; * corresponding authors: gerhard.lammel@recetox.muni.cz and celine.degrendele@recetox.muni.cz

Number of pages (including the cover page): 70

Number of text sections: 5

Number of figures: 14

Number of tables: 18

Tables of Contents:

Text: Materials and methods..S7

Sample preparation..S7

Sample analysis..S7

Quality assurance and quality control..S9

Data analysis S10

Literature review..S11

Figures:

Figure S1. Map of the 29 sites participating to this study. Abbreviations of the sampling site described in Tables S1..S11

Figure S2. 30 days footprint sensitivities (FLEXPART model) for the first, second and third sample collected at Zeppelin Observatory (ZPO) in which 0, 0 and 1 pesticide, respectively, were found in the particulate phase, in addition to, 1, 12 and 3 pesticides, respectively, found in the gaseous phase S12

Figure S3. 30 days footprint sensitivities (FLEXPART model) for the first, second and third sample collected at Andøya (ADA) in which each one pesticide was found in the particulate phase, in addition to one, three and four pesticides, respectively, found in the gaseous phase S13

Figure S4. 30 days footprint sensitivities (FLEXPART model) for the first, second and third sample collected at Pallas Atmosphere-Ecosystem Supersite (PAL) in which one, zero and two pesticides were found in the particulate phase, respectively..S14

Figure S5. 10 days backward trajectories (FLEXPART model) of the samples that were within the free troposphere at the mountain sites..S15

Figure S6. Number of pesticides quantified at each site in the particulate (all sites) and the gaseous phases (6 sites)..S16

Figure S7. Number of pesticides quantified in the particulate phase at each site. S17

Figure S8. Number of pesticides quantified in the particulate phase at each site category. The pesticides are classified according to their types and regulatory status in the European Union. S18

Figure S9. Concentrations in the particulate phase for the individual pesticides that showed a significant spatial variation between rural and coastal sites with the highest concentrations found at the rural sites S19

Figure S10. Concentrations in the particulate phase for those individual pesticides that do not show a significant spatial variation between the different site categories S21
Figure S11. Hierarchical cluster analysis performed on the particulate phase concentrations of pesticides measured during the 1st sampling period (28-30.04.2020).................................. S27
Figure S12. Hierarchical cluster analysis performed on the particulate phase concentrations of pesticides measured during the 2nd sampling period (12-14.05.2020) S28
Figure S13. Hierarchical cluster analysis performed on the particulate phase concentrations of pesticides measured during the 3rd sampling period (26-28.05.2020) S29
Figure S14. Pesticides identified as prone to long-range atmospheric transport (orange area, FT = free tropospheric), pesticides quantified in the planetary boundary layer (white area), and pesticides never observed (grey area) ordered along model estimate of characteristic travel distance. Not pictured is one pesticide never observed i.e., esbiothrin, due to unavailability of some input parameters. S30

Tables:

Table S1. Sampling methodology at the (A) rural, (B) coastal, (C) mountain and (D) polar sites. Site codes in bold where gas phase samples were also collected. S31
Table S2. Types of land-use within a 10 km radius for the rural, adjacent (A) and non-adjacent (B) to agricultural land, coastal (C), mountain (D) and polar (E) sites S35
Table S3. Allocation of this study land use categories to Corine Land Cover (A) and Global Land Cover (B) categories S37
Table S4. Information on the chemical analysis performed and standards used for individual herbicides (A), insecticides (B) and fungicides (C) S38
Table S5. Information on the individual herbicides (A), insecticides (B) and fungicides (C) analysed in this study S41
Table S6. Concentrations of individual pesticides (in pg m^{-3}) observed in field blanks (FB, QFFs) for the rural (A), coastal (B), mountain and polar (C) sites. The average sampled volume at each site was used to estimate the concentrations. The cells in blue and orange

represent results lower than the instrumental limit of detection (iLOD) and the instrumental limit of quantifications (iLOQ), respectively. S47

Table S7. Concentrations of individual pesticides (in pg m^{-3}) observed in field blanks (FB, PUF-XAD2-PUF sandwiches) at all sites. The average sampled volume at each site was used to estimate the concentrations. The cells in blue and orange represent results lower than the instrumental limit of detection (iLOD) and the instrumental limit of quantifications (iLOQ), respectively........ S50

Table S8. Origin of air samples collected at mountain sites S51

Table S9. Quantification frequency (QF, in %) per site of individual pesticides present in the particulate phase S52

Table S10. Summary of pesticides previously quantified in the atmosphere of polar sites. . S53

Table S11. Physico-chemical properties and half-lives in different environmental matrices of the individual herbicides (A), insecticides (B) and fungicides (C) analysed in this study S54

Table S12. Average particulate mass fraction (θ, %) of pesticides measured at both rural and polar sites where both phases were sampled S57

Table S13. Selected HPLC-MS/MS experimental parameters, instrumental limits of detection (iLODs) and instrumental limits of quantification (iLOQs) S58

Table S14. Selected GC-MS/MS and UPLC-MS/MS experimental parameters, instrumental limits of detection (iLODs) and instrumental limits of quantification (iLOQs) S59

Table S15. Limits of quantifications determined from field blanks (LOQb) (in pg m^{-3})...... S60

Table S16. Recoveries (in %) and standard deviations (SD) of individual herbicides (A), insecticides (B) and fungicides (C) analysed using HPLC-MS/MS determined from spike recovery tests of air sampling media (QFFs and PUF-XAD2-PUF sandwiches, n=5 each) . S61

Table S17. Results of the multiple linear regression statistical analysis applied to the number of pesticides found at each site S64

Table S18. Summary of pesticides previously quantified in the atmosphere at high mountain sites. S65

References S66

Materials and Methods

Sample preparation

Prior to the extraction, all samples were spiked with labelled standards (Table S4). The samples were extracted with 5 mM of ammonium acetate in methanol using an automatic extractor (Büchi Extraction System, B-811, Switzerland). The extraction consisted of one-hour warm Soxhlet followed by one hour of solvent rinsing and then a concentration step to 1 mL under a gentle stream of nitrogen. The concentrated extracts were centrifuged for 10 min (12000 G, Z-36 HK, Hermle Labortechnik, Germany) within polypropylene tubes (Corning Costar Spin-X, USA), then filtered (cellulose acetate membrane and 0.22 µm pore size) and concentrated to 0.5 mL under a gentle stream of nitrogen.

Sample analysis

After extraction, the samples were divided into three 100 µL aliquots, which underwent each a different analysis.

The first aliquot was analysed at RECETOX, in Brno (Czech Republic) by two different methods using a high-performance liquid chromatograph (HPLC, Agilent 1290, Agilent Technologies, USA) coupled to a mass spectrometer (QTRAP 5500, AB Sciex, Framingham, USA). The first analytical method used a Phenomenex Synergi Fusion C-18 column and formic acid in water and in methanol mobile phase gradient and has been explained in detail elsewhere [15]. This method allowed for the quantification of 28 analytes. Additionally, two new methods were developed using the same instruments allowing for the quantification of 23 analytes. Both of the new methods used an Acquity UPLC BEH C18 column (1.7 µm, 100 × 2.1 mm i.d., Waters, USA). However, the first new method used a non-linear binary pump gradient (increase in the mobile phase from 95% 0.1% formic acid in water (A) and 5% 0.1% formic acid in methanol (B) at 0 minutes to 100% B at 5.75 minutes, then decrease to 5% B at 8.5 minutes for equilibration at initial conditions (5% B). The total time of analysis was 11.5 min with a flow rate of the mobile phase of 0.30 mL min^{-1}. For this method, 10 µL of individual sample was injected. Ions were detected in positive mode. The ionization parameters were as follows: desolvation temperature 550°C; curtain gas 35 psi, ion source gas 1 30 psi and ion source gas 2 at 60 psi. For the second new method, a non-linear binary pump gradient was used (increase in the mobile phase from 95% 1 mM ammonium acetate in water (A) and 5% 1 mM ammonium acetate in acetonitrile (B) at 0 minutes to 100% B at 5.75 minutes, then decrease to 5% B at 8.5

minutes for equilibration at initial conditions (5% B). The total time of analysis was 10 min with a flow rate of the mobile phase of 0.40 mL min^{-1}. For this method, 10 µL of individual sample was injected. Ions were detected in negative mode. The ionization parameters were as follows: desolvation temperature 450°C; curtain gas 20 psi, ion source gas 1 50 psi and ion source gas 2 at 30 psi. The remaining parameters were the same as for the initial method, previously described [15].

The second and third methods were analysed at the Laboratory of Chemistry and Environment, Aix-Marseille University, Marseille (France) and required a solvent transfer from methanol to acetonitrile. A gas chromatograph (GC, GC Ultra, Thermo Scientific, USA) coupled to a tandem mass spectrometer (MS/MS, TSQ QuantumTM triple quadrupole, Thermo Scientific, USA) using electron impact ionization and an ultra-performance liquid chromatograph (UPLC Acquity, Waters, USA) coupled to a MS/MS (quadrupole time-of-flight mass spectrometer Synapt G2 HDMS, Waters, USA) equipped with an electrospray ion source were used. Further details about these methods are described elsewhere [16]. However, different settings were used for the GC-MS/MS analysis: the interface temperature was 250 °C and the holding step was 3 min at 35 °C.

The precursor to product ions were monitored in scheduled multiple reaction monitoring mode (MRM) (Tables S13-and S14). The identification of individual pesticides was based on the comparison of intensity ratios of ions and retention times with standards and quantification was done using internal calibration with deuterated standards (Table S4).

Overall, the three analyses allowed for the quantification of 76 pesticides (35 herbicides, 22 insecticides and 19 fungicides) belonging to 34 chemical classes (Table S5). These compounds were chosen according to results from previous monitoring studies [15,16], but also global pesticide usage [72] and their potential harmful effects to environmental and human health [73,74]. Among these 76 CUPs analysed (based on the analytical methods available), 40 have been approved for agricultural use in Europe [75], 22 are among the most used pesticides globally [72], 34 are characterized as priority active ingredients that need to be monitored in France [73], 13 as highly hazardous pesticides and 25 as high-risk pesticides [74].

Quality assurance and quality control

In addition to the air samples collected, 35 field blanks (i.e., 29 QFFs and six sandwiches of PUF/XAD/PUF) were analysed as per sample. These field blanks were placed for several seconds within the sampler without air being pumped. Moreover, five solvent blanks were also analysed. In general, blank levels of most individual analytes were below detection or otherwise low (Tables S6 and S7). The concentrations of individual pesticides reported here were blank corrected by subtracting the average of the corresponding field blanks.

The concentrations measured in the samples were compared to the instrumental limits of detection (iLODs) and quantification (iLOQs), which were determined by distinguishing the quantity of analytes with a signal-to-noise ratio of 3:1 and 10:1, respectively. We defined the limit of quantification as the maximum between iLOQ and the limit of quantification determined from field blanks (LOQb). LOQb were determined as the average of the concentration of a pesticide found in the field blanks plus three-times their standard deviations (Table S15). Therefore, LOQb allows for discriminating between “contaminated” blanks and analyte concentration present in environmental samples.

The recoveries of individual pesticides were assessed by spiking sampling media (i.e., five QFFs and five PUF/XAD2/PUF sandwiches) with the native standards and their corresponding isotopic labelled standards, which were then processed as per sample.

For the 51 pesticides analysed using the HPLC-MS/MS, the recoveries of individual analytes ranged from 52 % ± 27 (iprovalicarb) to 152 % ± 20 (fenpropimorph) for QFFs and from 34.1 % ± 13 (diazinon) to 116 % ± 5.90 (phosalone) for sandwiches (Table S16). Besides few exceptions, most of the procedural recoveries were in the range of 60–120% and had standard deviations lower than 20%. For these 51 pesticides, the concentrations have been adjusted for recoveries by the instrumental software during data acquisition and treatment of the selected precursor-to-products ions reaction mass chromatograms. For the remaining pesticides, the extraction recoveries were taken into account for calibration by applying the experimental protocol used for the samples to a blank matrix spiked with calibration standards. For these analyses, the recovery results were within the acceptable range, 60-120%.

Data analysis

All statistical analyses were performed using the software GraphPad Prism® (v9.0.0). Concentrations that were lower than iLOD, iLOQ or LOQb were not taken into account. Substitutions of values below LOQ by LOQ/2 were used to determine the relative standard deviation (Table 1).

The quantification frequencies per site of individual pesticides were calculated (Table S9) as well as the number of pesticides quantified in the particulate phase at each site (Figures S3 and S4).

Multiple linear regressions were performed to study a possible influence of the site category and the percentage of agricultural areas surrounding each site on the number of pesticides quantified in the particulate phase and on the concentrations of individual pesticides (Table S17). For those parameters that were significant, regressions were tested (Figure 1). The influence of latitude on the number of pesticides found in European air was assessed by using a 3rd order polynomial regression to test the null hypothesis, which expects the pesticide distribution to follow the cropland's meridional distribution (Figure 1). In the study region (30 – 80°N/-10 – 30°E), the regression shows a minimum at latitudes with high area fraction of sea and a maximum at central European latitudes.

A hierarchical cluster analysis was performed to assess the similarities in terms of the particulate concentration profile of pesticides at the 29 sites (Figures S7 to S9). This analysis was performed for each of the three sampling periods.

The differences in the concentrations of pesticides measured at each site category were assessed using a non-parametric Mann-Whitney test when concentrations were available for at least two categories while a non-parametric Kruskal-Wallis test was used when data were available for three or four categories of sites (Figures S5 and S6). We considered a *p*-value <0.05 to be statistically significant.

Figure 2, S1 and S2 were done using the geographic information system software ArcGIS pro® (v2.5.0) and Figure 3 using Microsoft Excel® (v2202).

Literature review

A review on the current-use pesticides (other than the legacy organochlorine pesticides) that were previously reported in the atmosphere of polar and mountain sites was performed (Tables S10 and S18).

In addition, information related to organic matter concentrations was taken from the EBAS Database (https://ebas.nilu.no/).

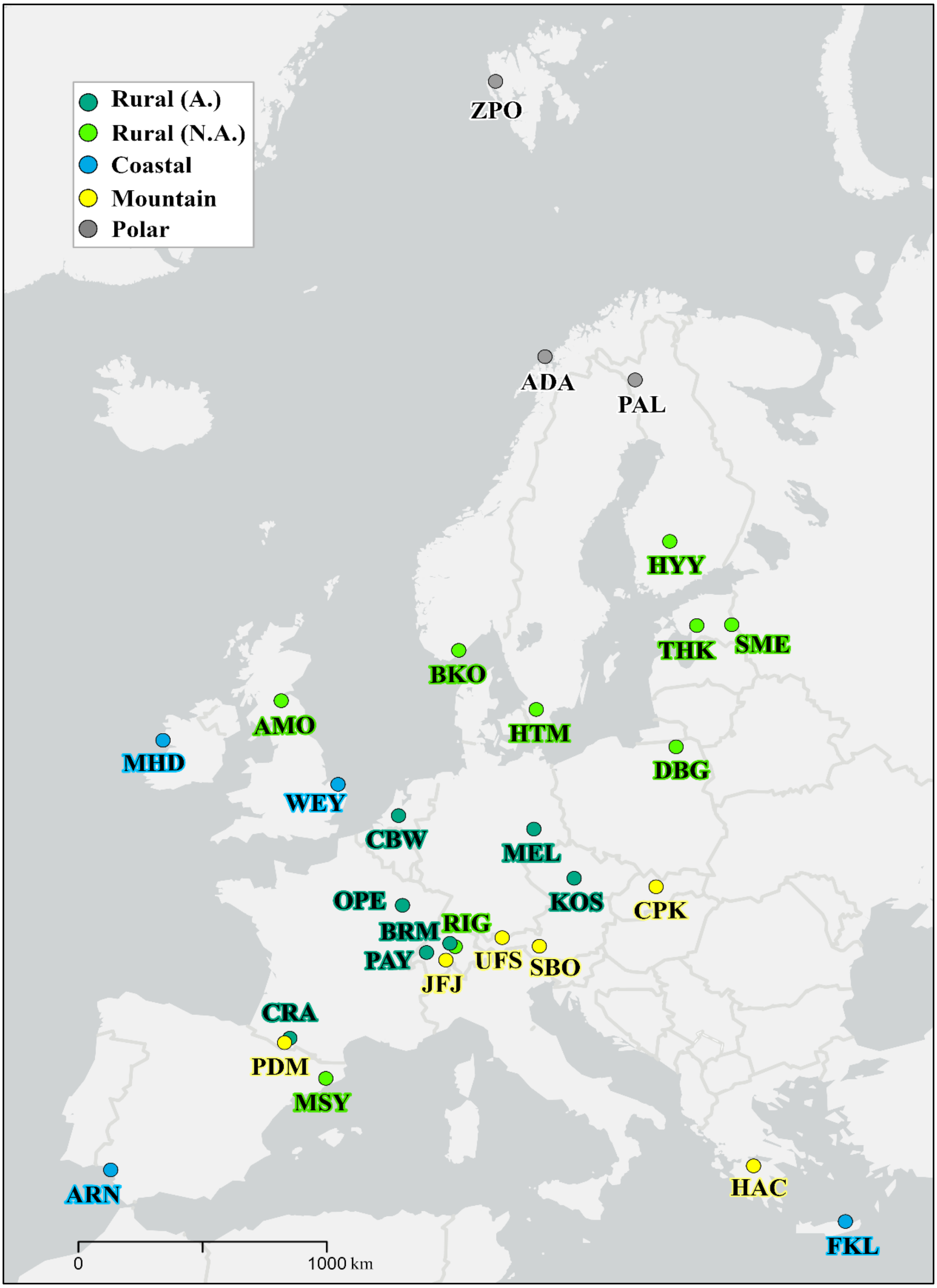

Figure S1. Map of the 29 sites participating to this study. Abbreviations of the sampling site described in Tables S1

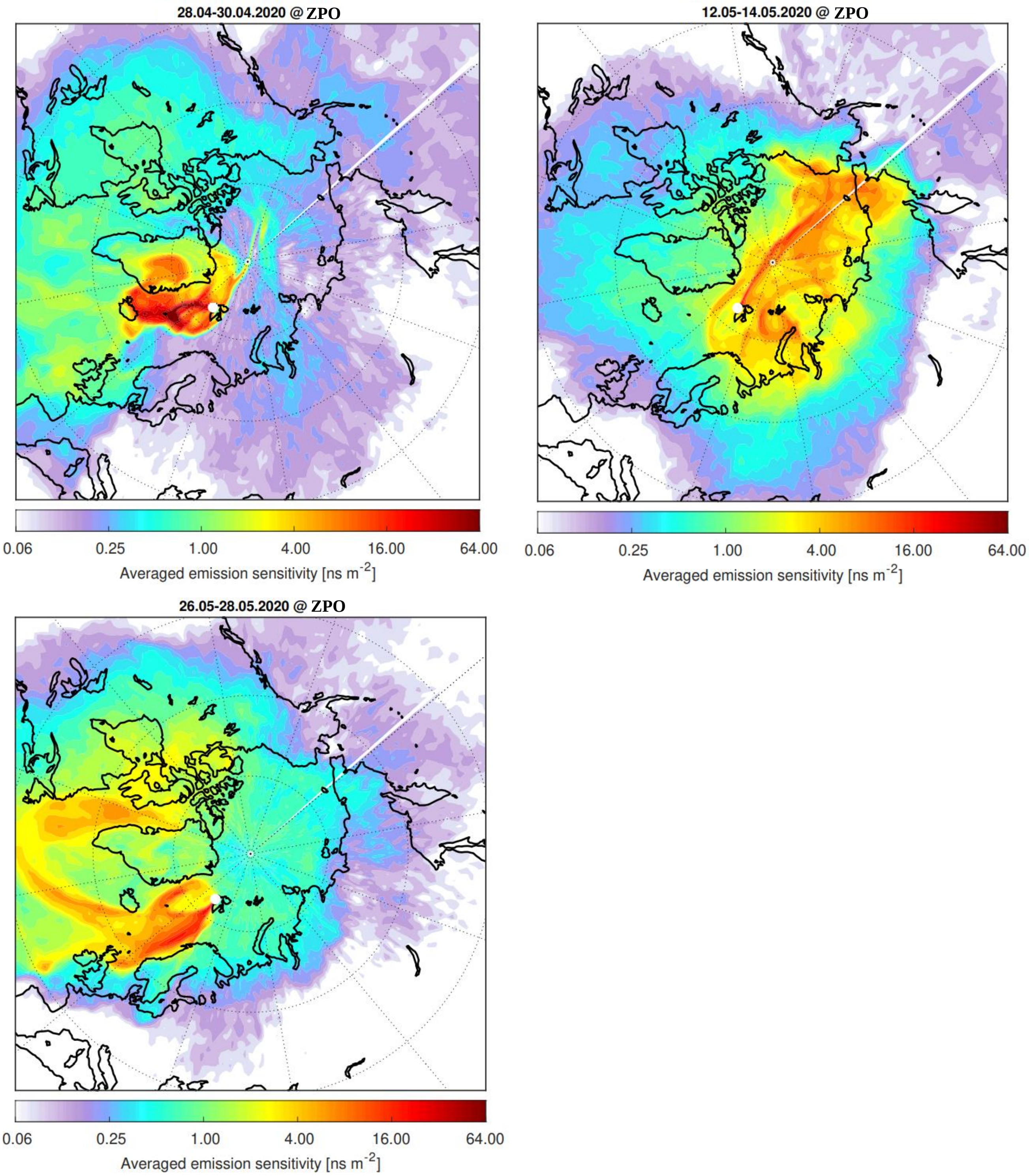

Figure S2. 30 days footprint sensitivities (FLEXPART model) for the first, second and third sample collected at Zeppelin Observatory (ZPO) in which 0, 0 and 1 pesticide, respectively, were found in the particulate phase, in addition to, 1, 12 and 3 pesticides, respectively, found in the gaseous phase

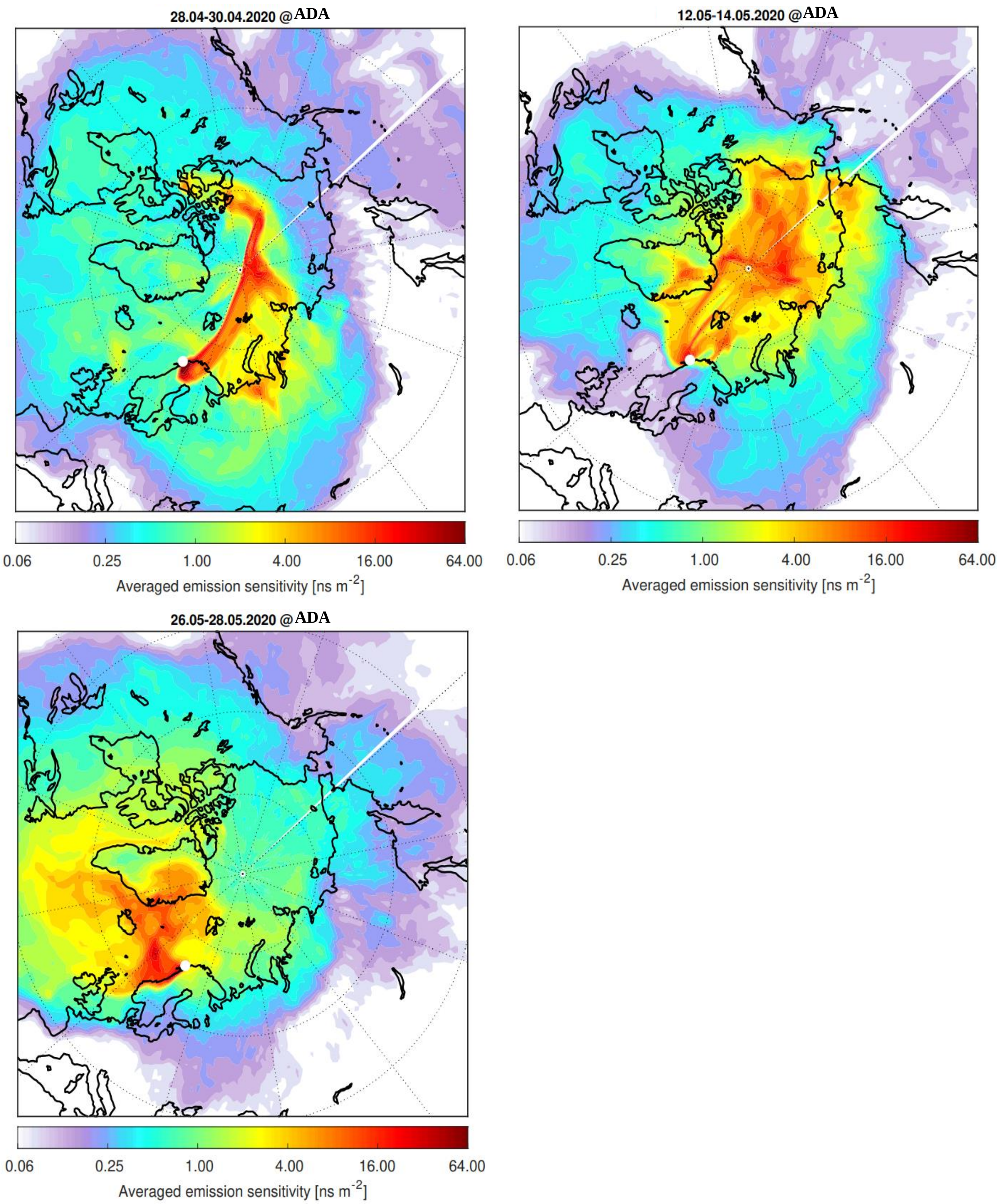

Figure S3. 30 days footprint sensitivities (FLEXPART model) for the first, second and third sample collected at Andøya (ADA) in which each one pesticide was found in the particulate phase, in addition to one, three and four pesticides, respectively, found in the gaseous phase

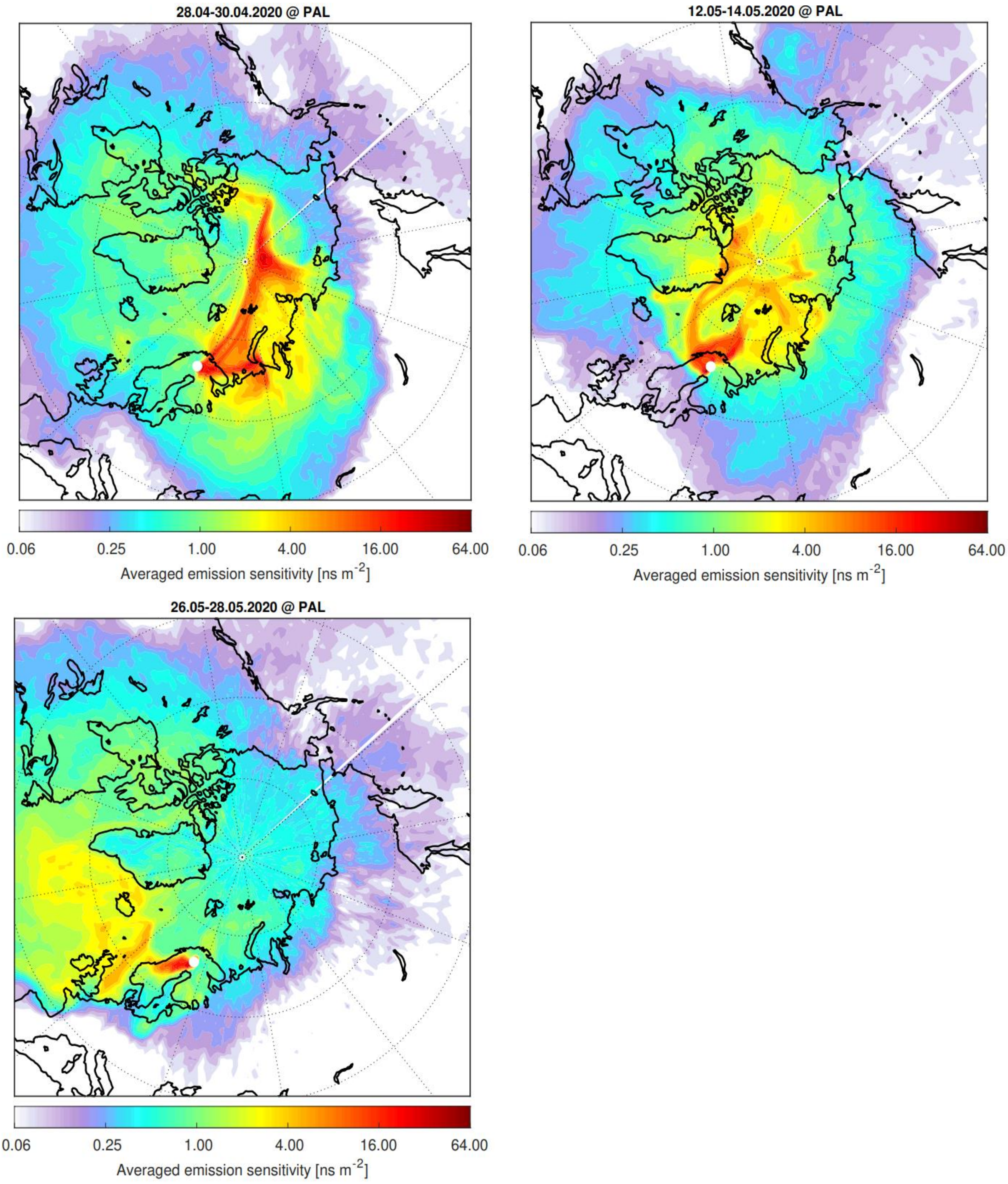

Figure S4. 30 days footprint sensitivities (FLEXPART model) for the first, second and third sample collected at Pallas Atmosphere-Ecosystem Supersite (PAL) in which one, zero and two pesticides were found in the particulate phase, respectively

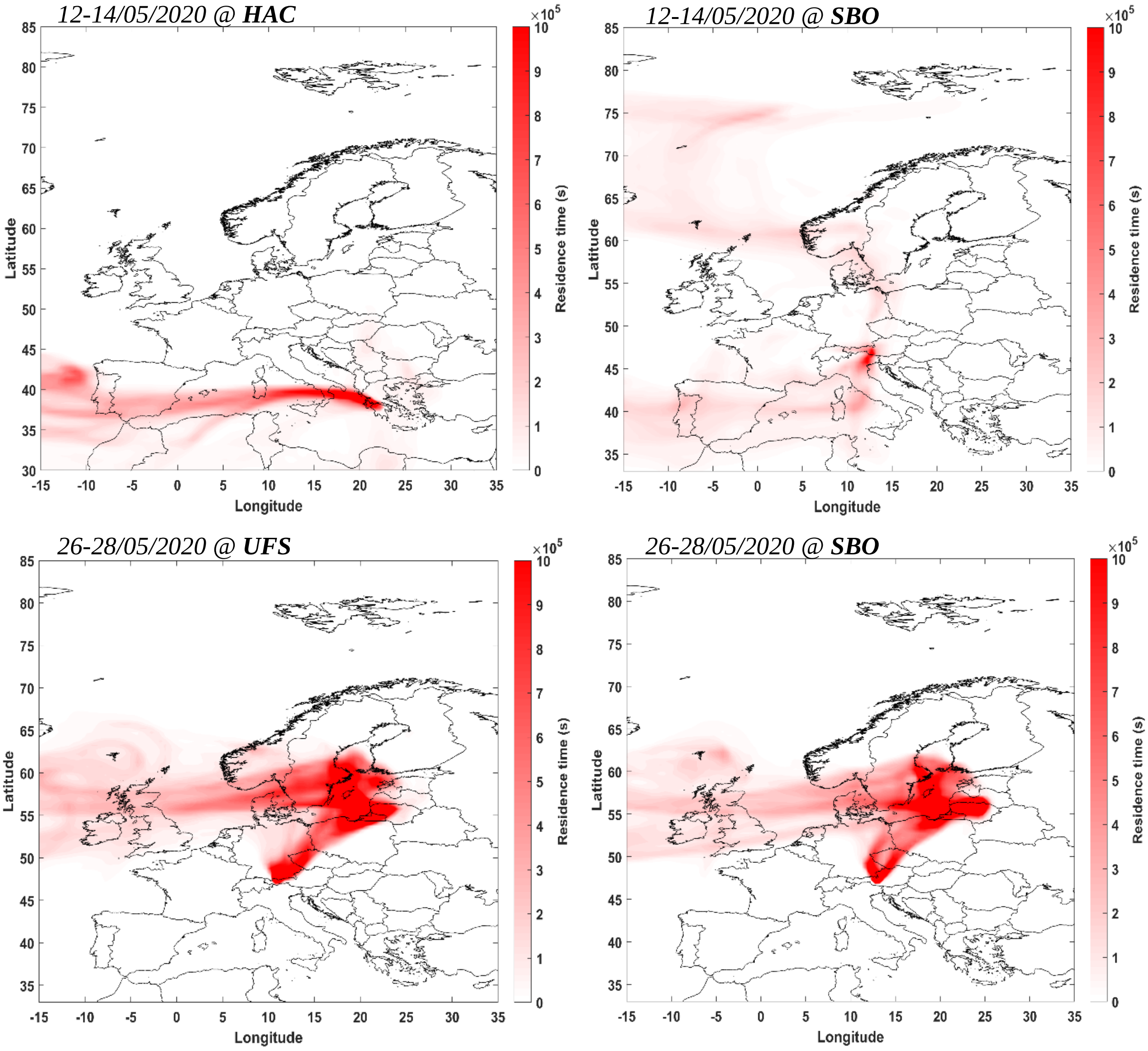

Figure S5. 10 days backward trajectories (FLEXPART model) of the samples that were within the free troposphere at the mountain sites

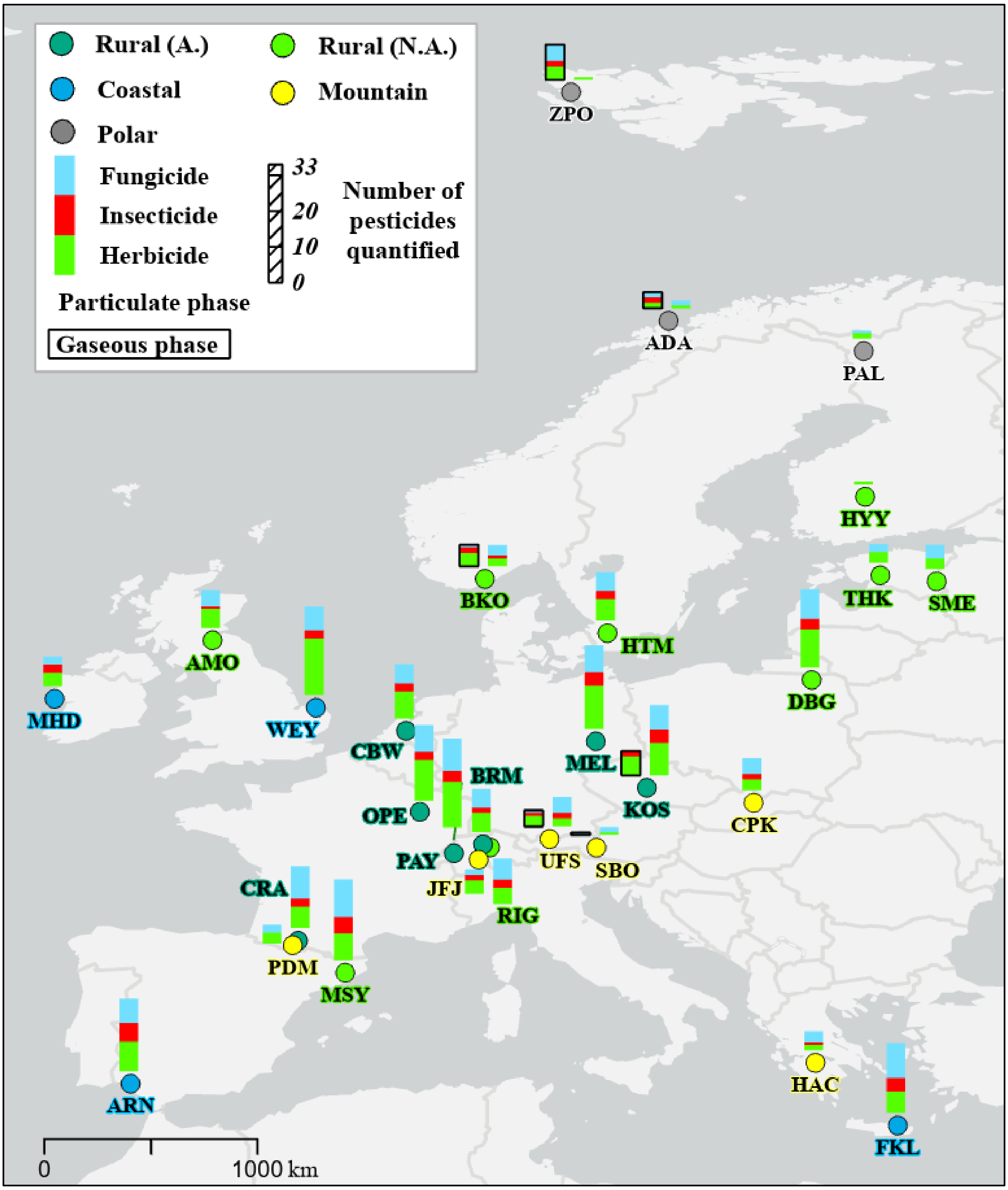

Figure S6. Number of pesticides quantified at each site in the particulate (all sites) and the gaseous phases (6 sites).

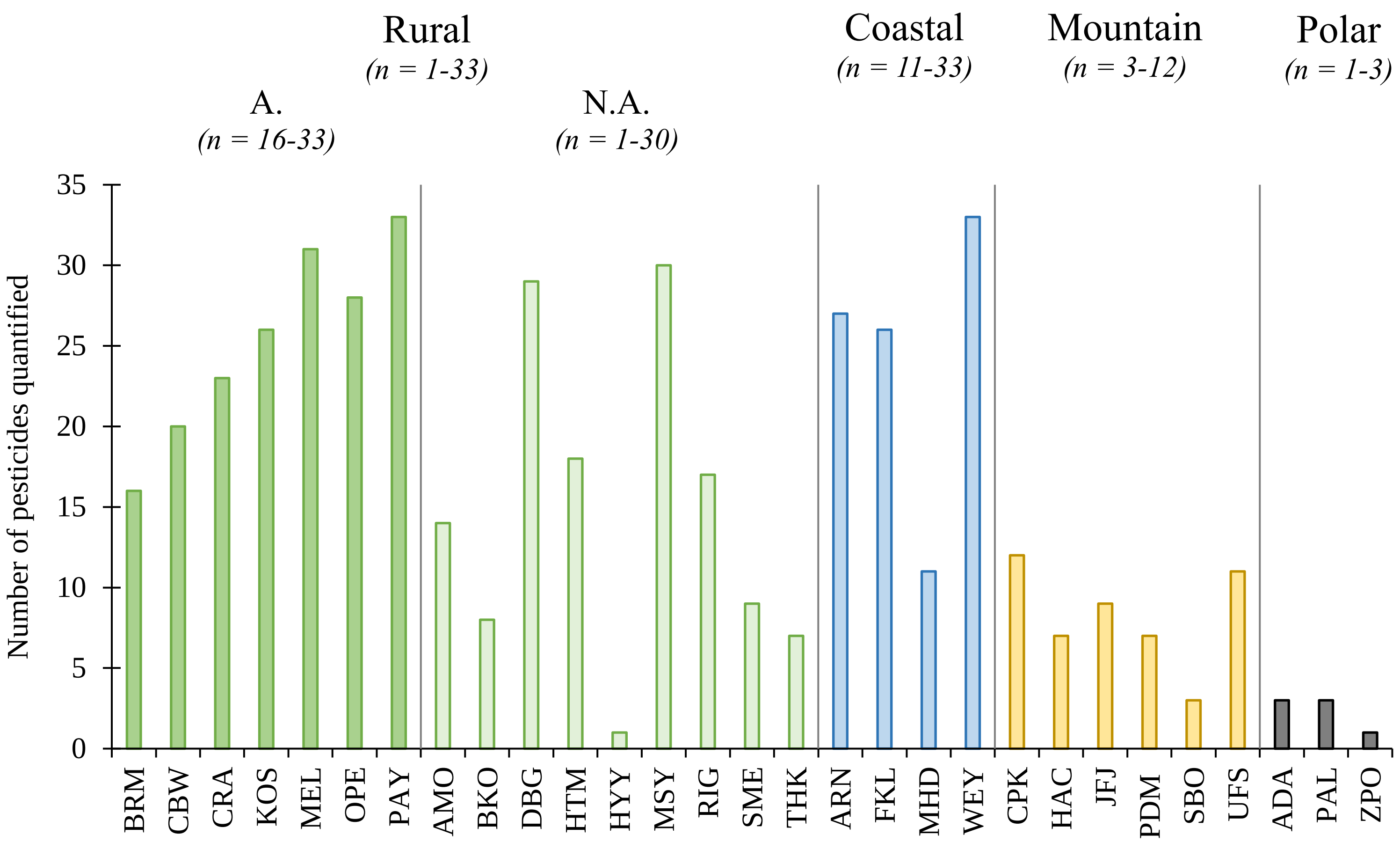

Figure S7. Number of pesticides quantified in the particulate phase at each site.

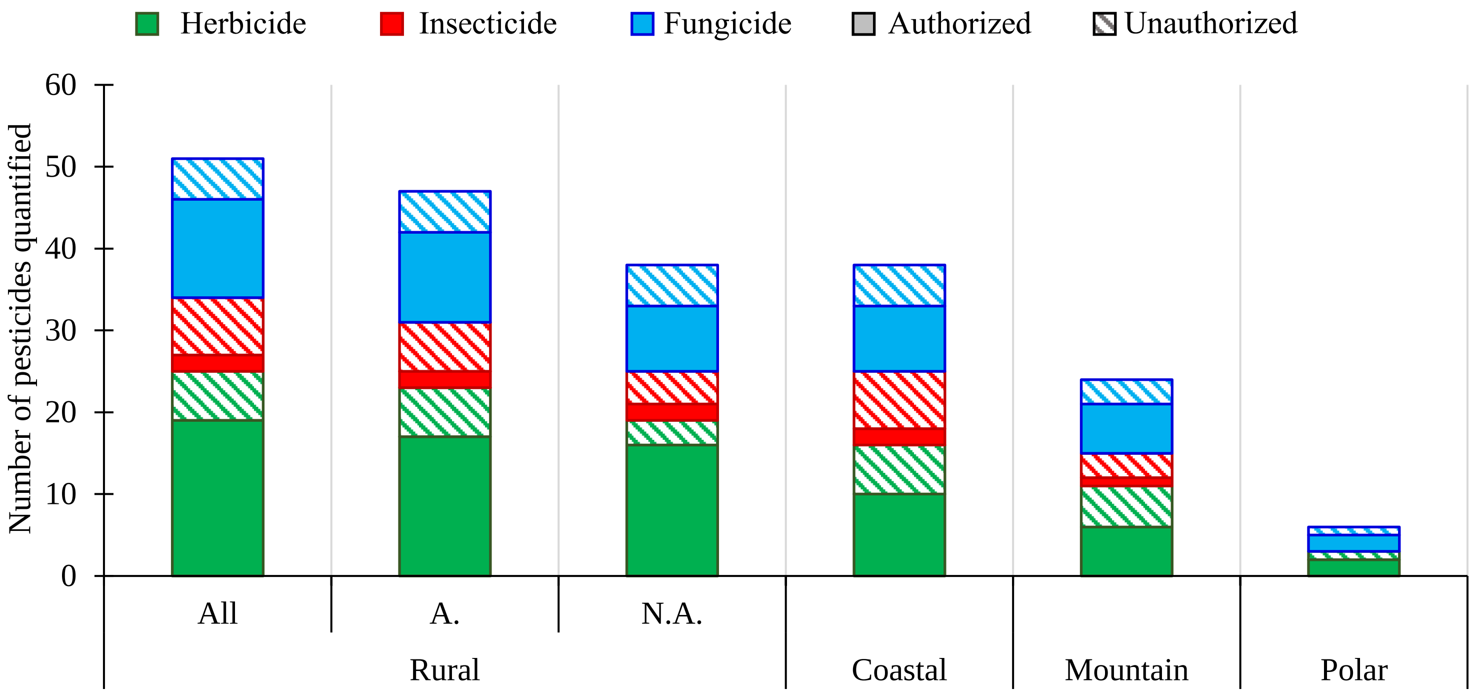

Figure S8. Number of pesticides quantified in the particulate phase at each site category. The pesticides are classified according to their types and regulatory status in the European Union.

The site categories used were rural (n=16) including agricultural adjacent sites (A., n=7) and non-agricultural adjacent sites (N.A., n=9), coastal (n=4), mountain (n=6) and polar sites (n=3).

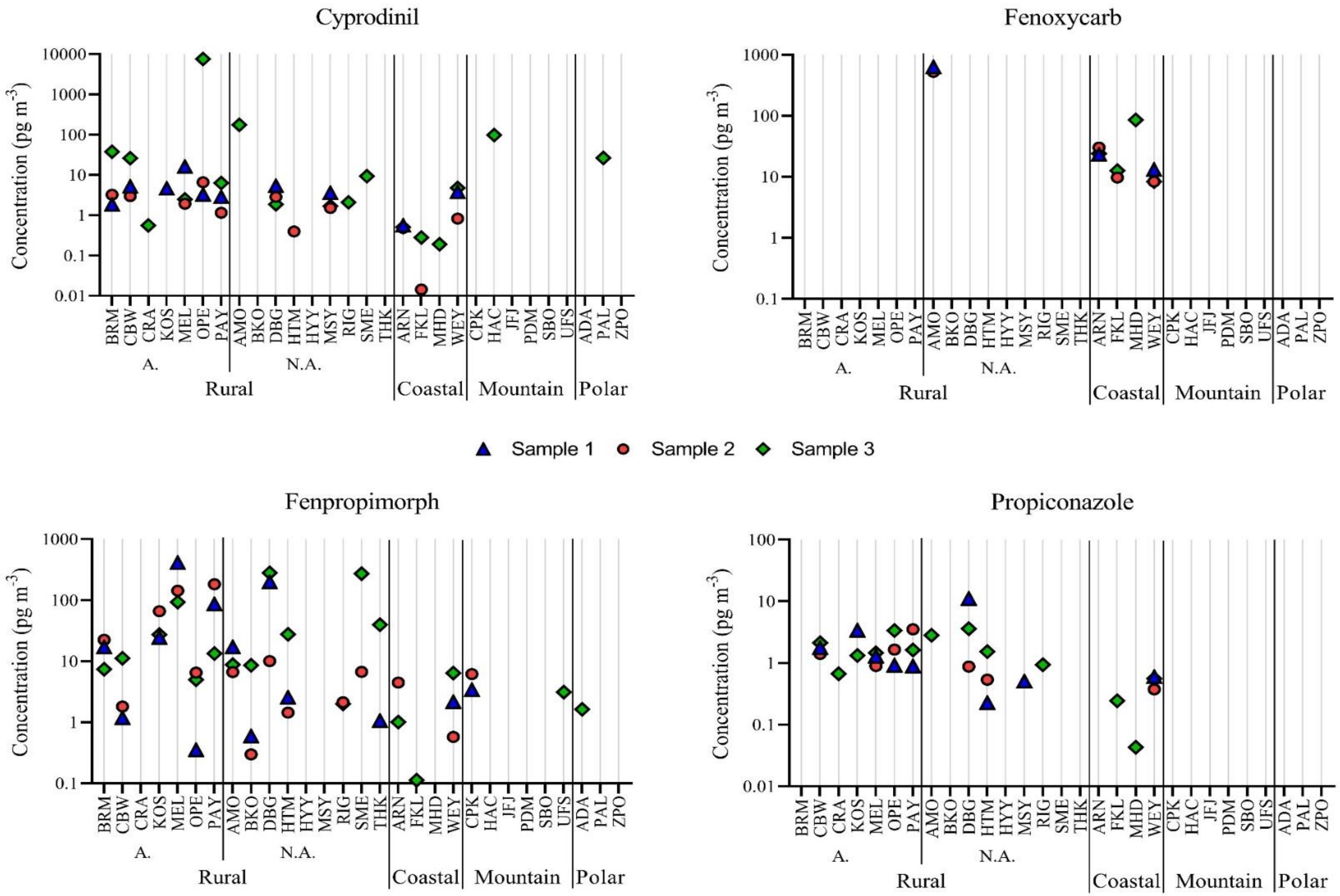

Figure S9. Concentrations in the particulate phase for the individual pesticides that showed a significant spatial variation between rural and coastal sites with the highest concentrations found at the rural sites

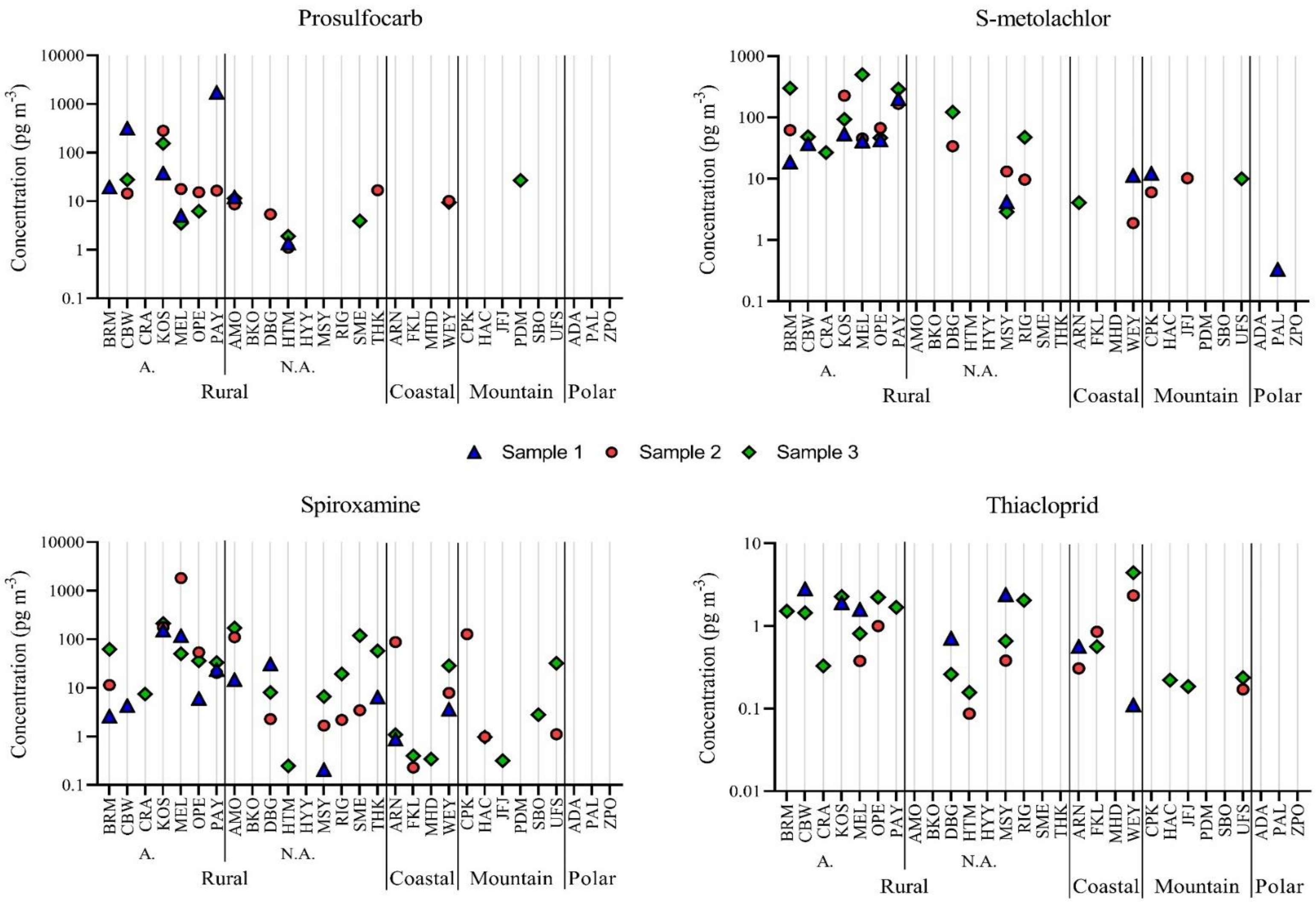

Figure S9. Concentrations in the particulate phase for the individual pesticides that showed a significant spatial variation between rural and coastal sites with the highest concentrations found at the rural sites (continued)

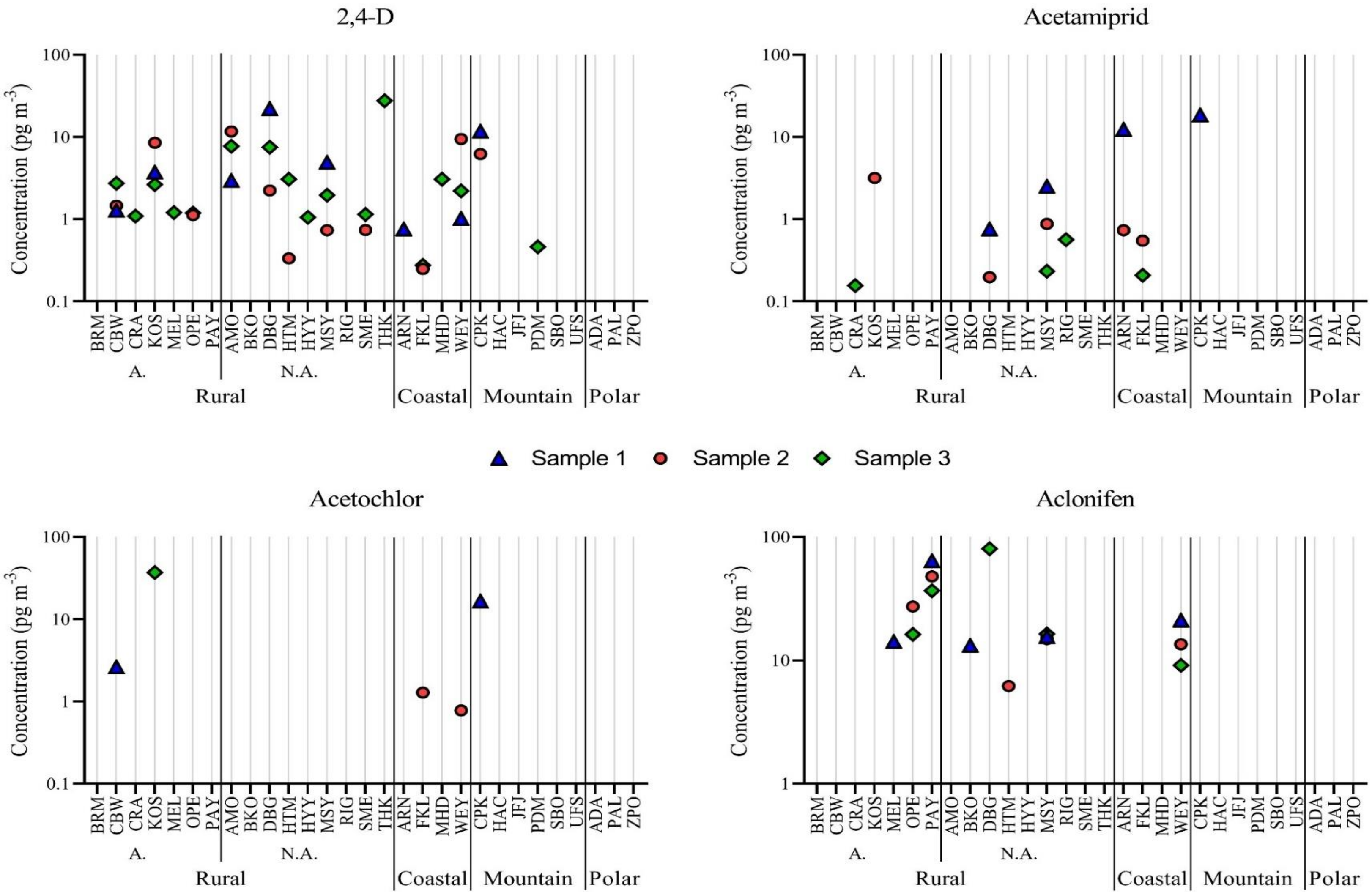

Figure S10. Concentrations in the particulate phase for those individual pesticides that do not show a significant spatial variation between the different site categories

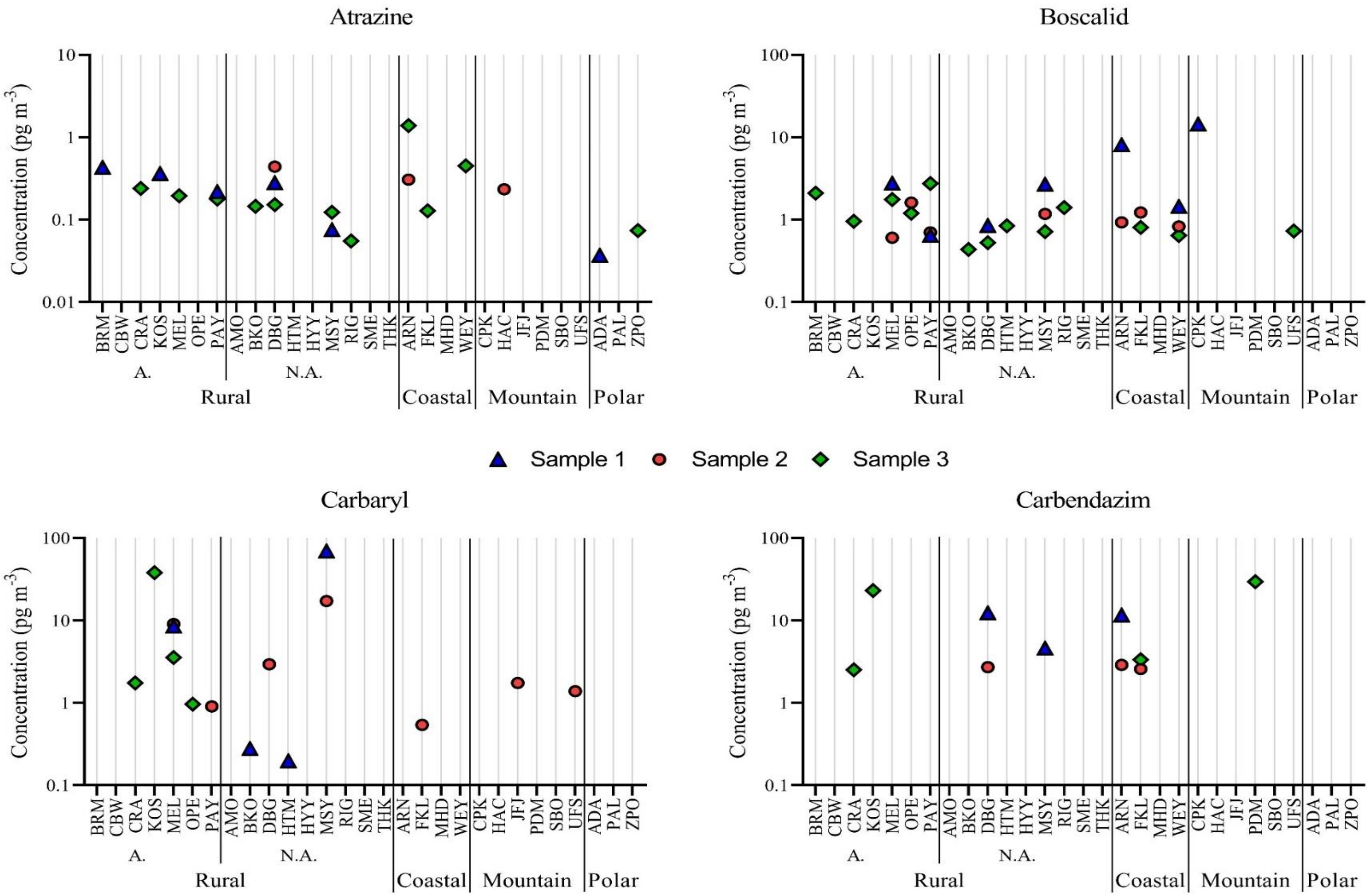

Figure S10. Concentrations in the particulate phase for those individual pesticides that do not show a significant spatial variation between the different site categories (continued)

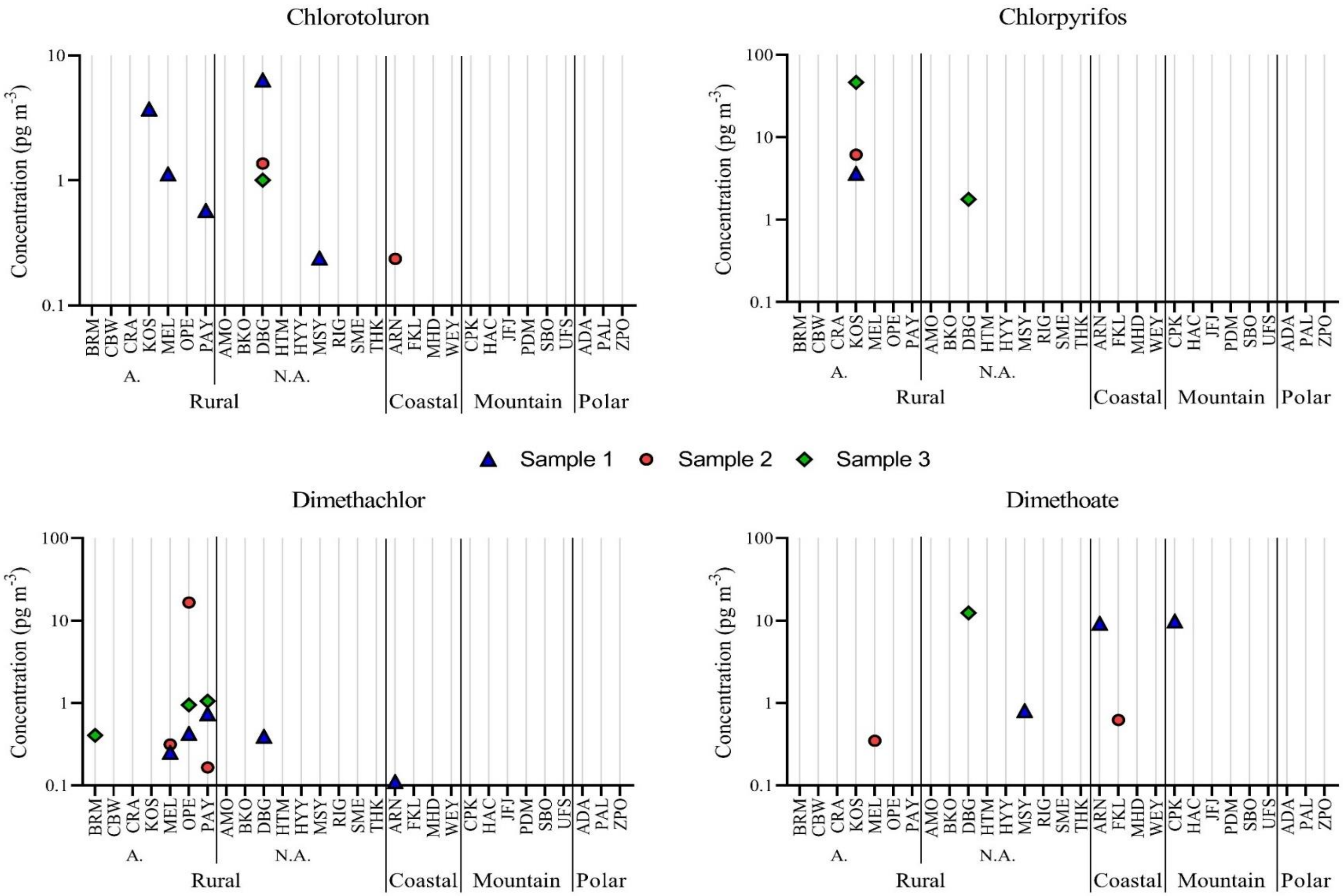

Figure S10. Concentrations in the particulate phase for those individual pesticides that do not show a significant spatial variation between the different site categories (continued)

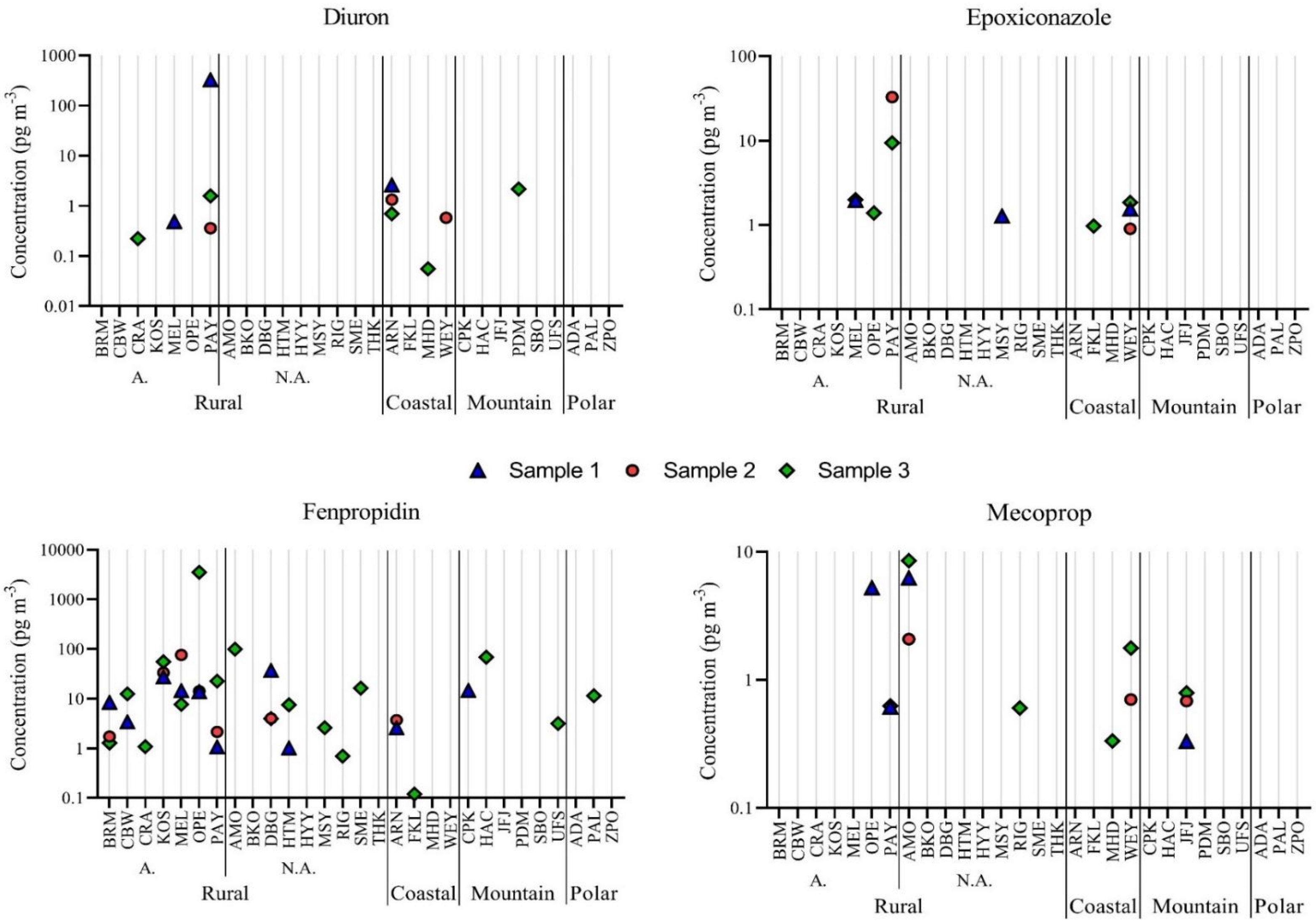

Figure S10. Concentrations in the particulate phase for those individual pesticides that do not show a significant spatial variation between the different site categories (continued)

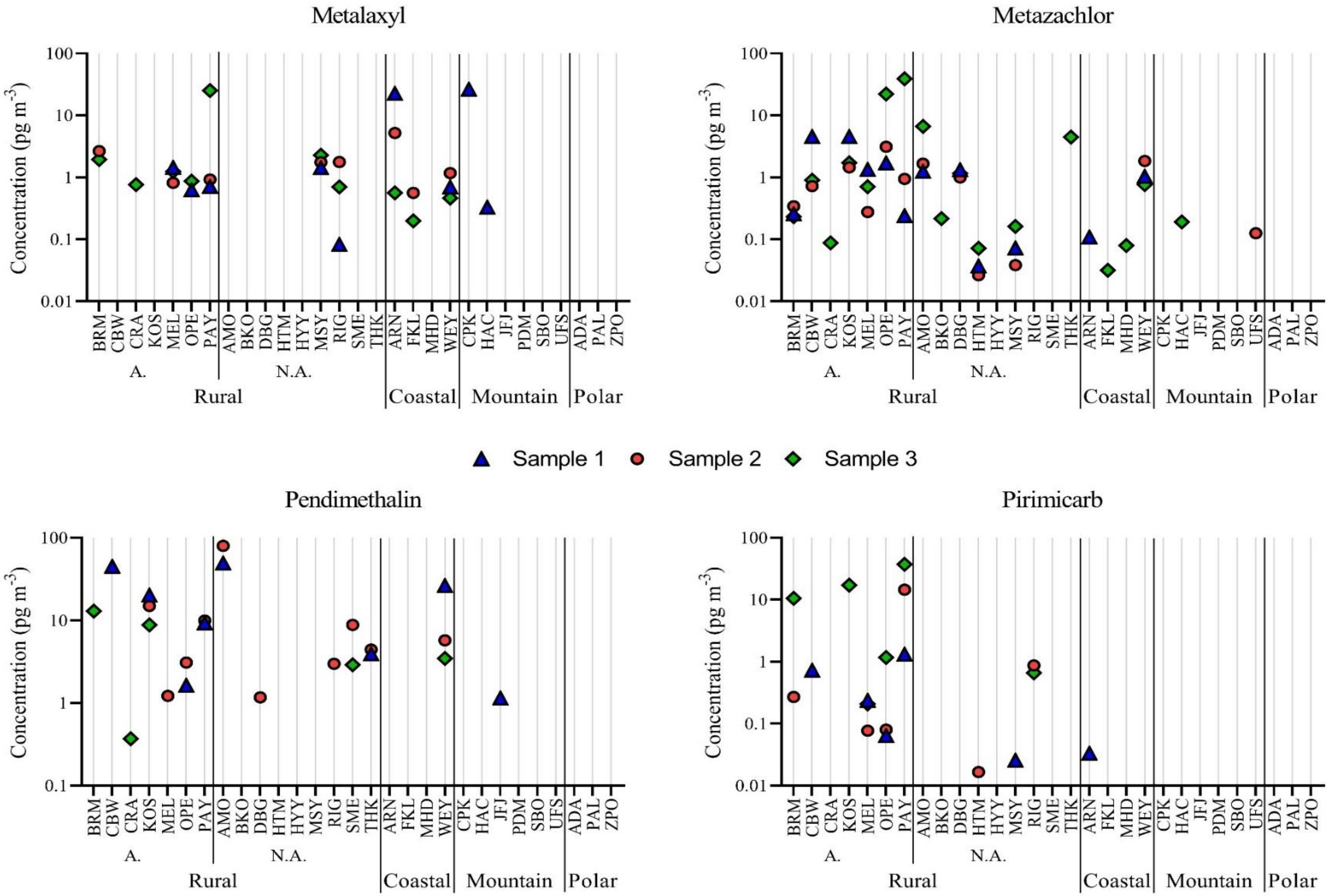

Figure S10. Concentrations in the particulate phase for those individual pesticides that do not show a significant spatial variation between the different site categories (continued)

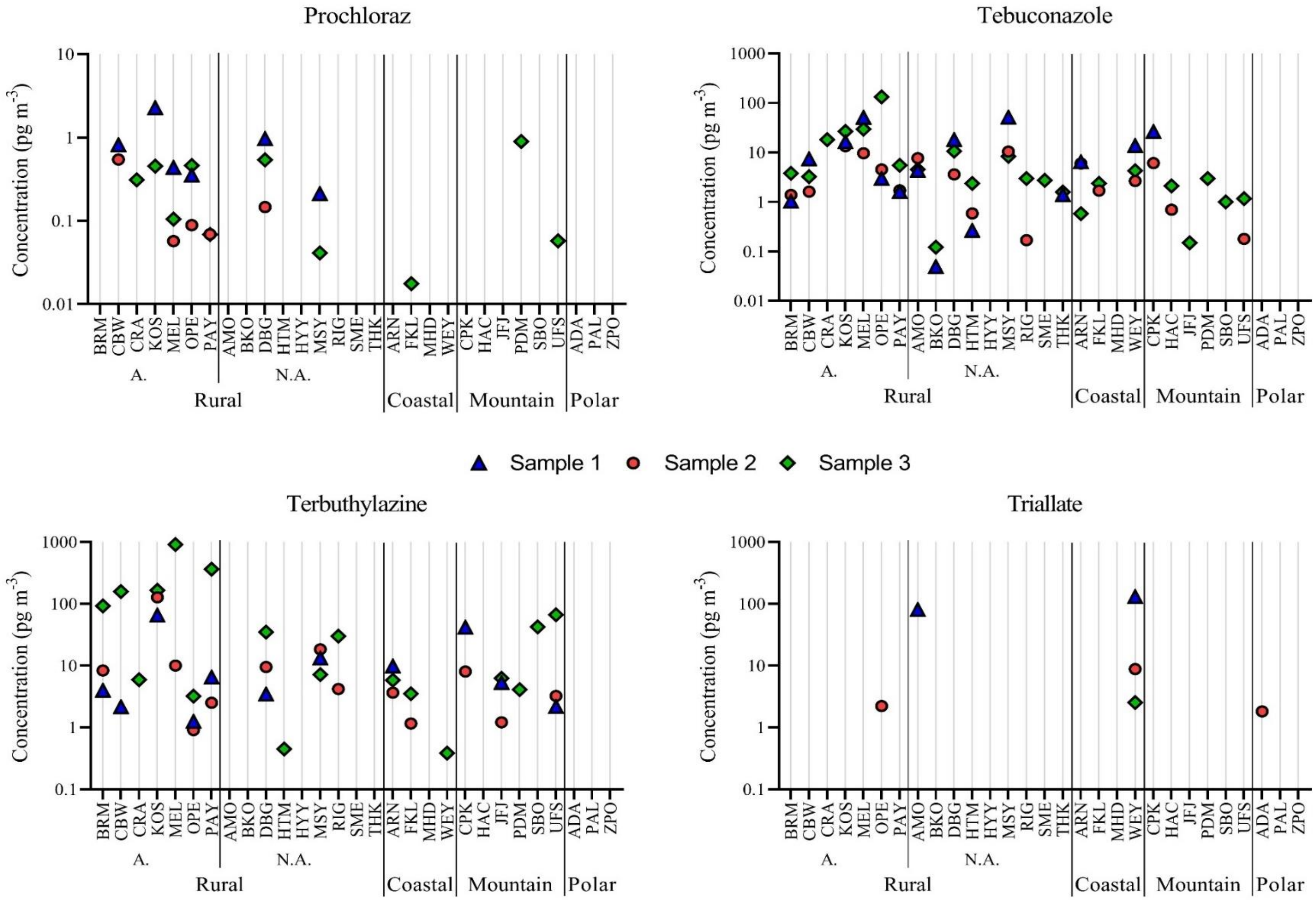

Figure S10. Concentrations in the particulate phase for those individual pesticides that do not show a significant spatial variation between the different site categories (continued)

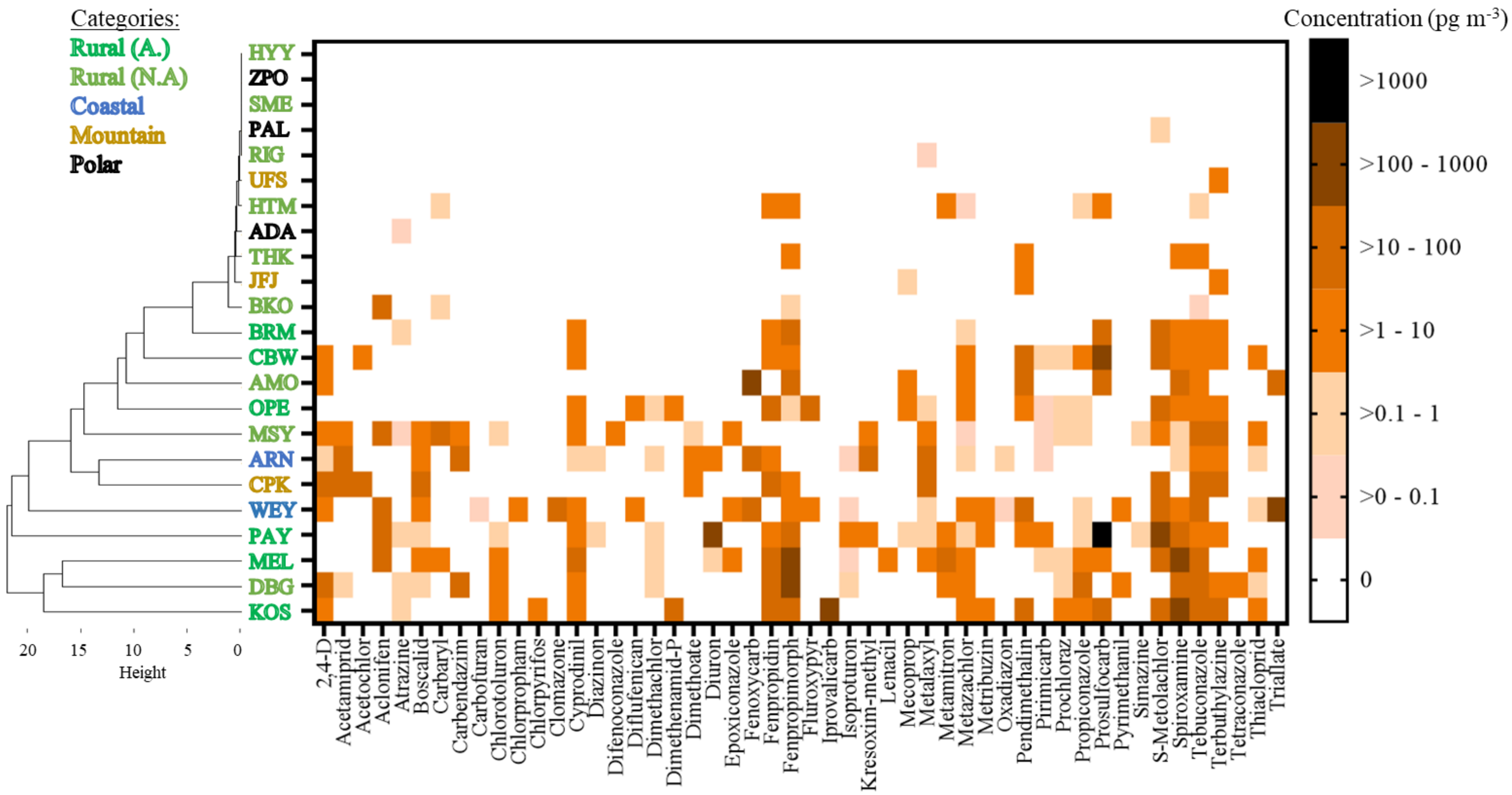

Figure S11. Hierarchical cluster analysis performed on the particulate phase concentrations of pesticides measured during the 1st sampling period (28-30.04.2020)

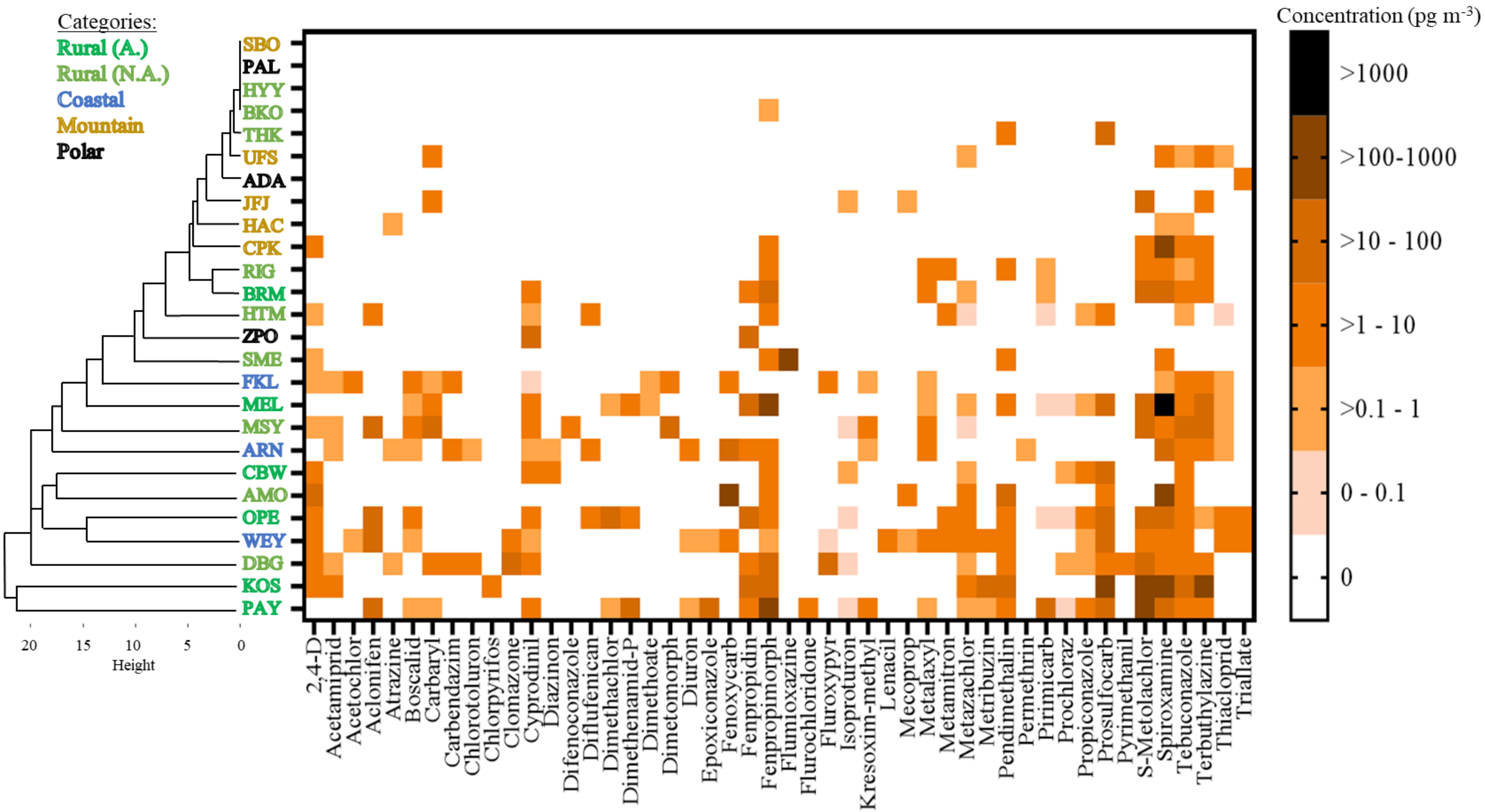

Figure S12. Hierarchical cluster analysis performed on the particulate phase concentrations of pesticides measured during the 2nd sampling period (12-14.05.2020)

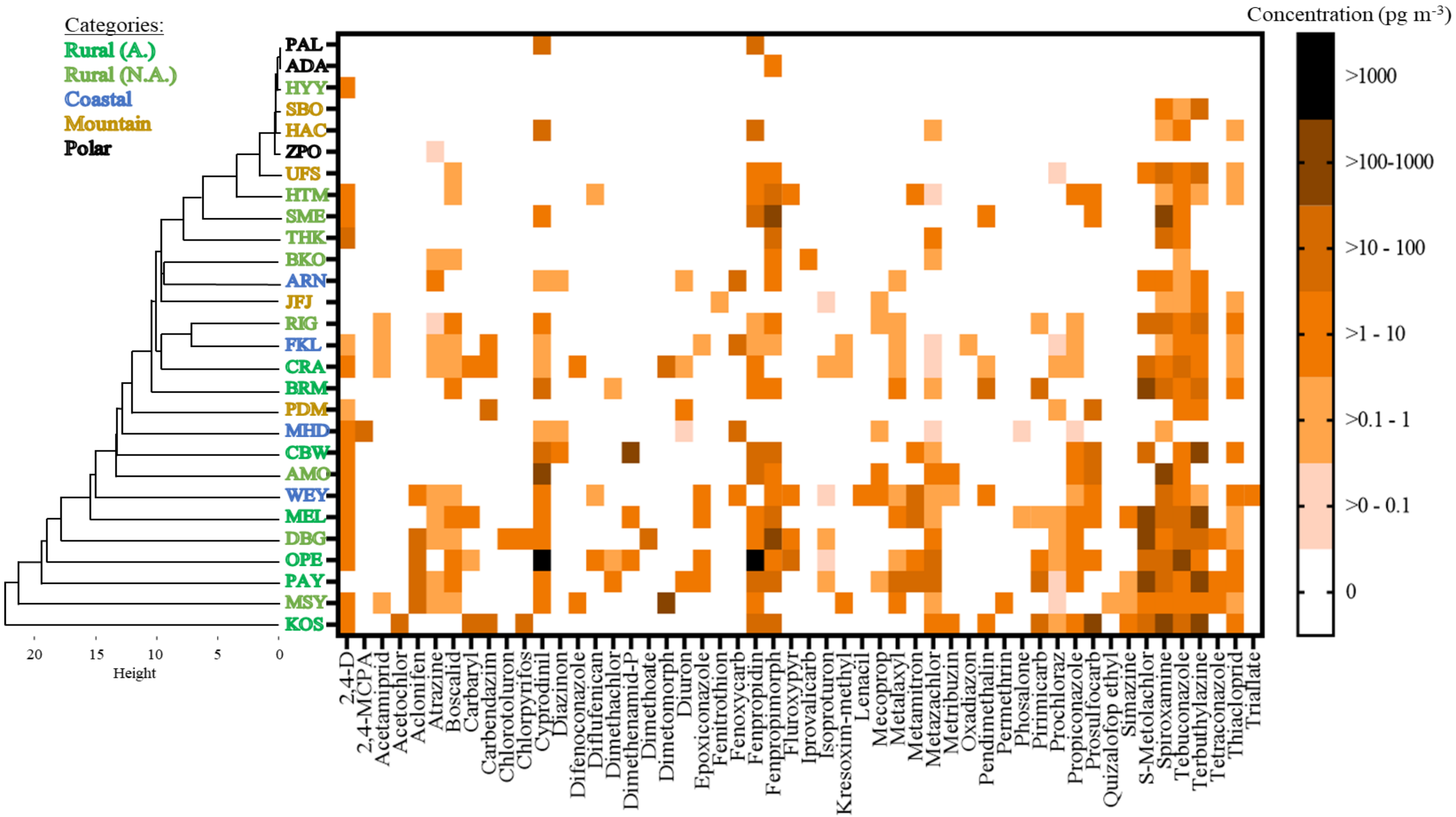

Figure S13. Hierarchical cluster analysis performed on the particulate phase concentrations of pesticides measured during the 3rd sampling period (26-28.05.2020)

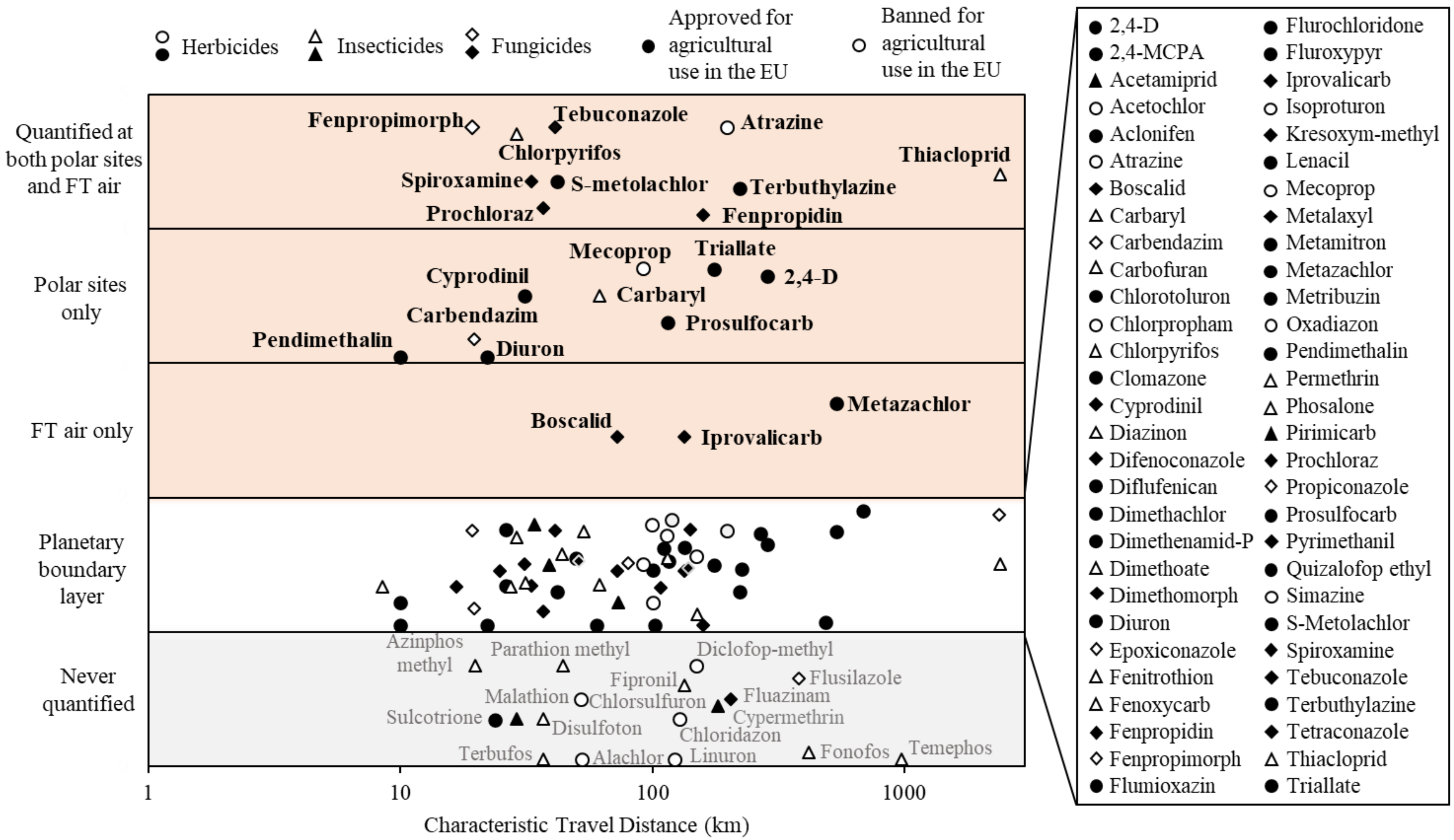

Figure S14. Pesticides identified as prone to long-range atmospheric transport (orange area, FT = free tropospheric), pesticides quantified in the planetary boundary layer (white area), and pesticides never observed (grey area) ordered along model estimate of characteristic travel distance. Not pictured is one pesticide never observed i.e., esbiothrin, due to unavailability of some input parameters.

Table S1. Sampling methodology at the (A) rural, (B) coastal, (C) mountain and (D) polar sites. Site codes in bold where gas phase samples were also collected.

(A)

Site code	Sampling site	Rural subcategories	Sampler type	Sampler used	Inlet particle size fraction	Flow-rate (m^3 h^{-1})	Sampled volume (m^3) Average [Min-Max]	Filter QFF	Number of samples collected
AMO	Auchencorth Moss	NA	LVAS	PARTISOL 2025 (Thermo Fisher Scientific, Waltham, USA)	PM_{10}	1	48	b	3
**BKO**	**Birkenes Observatory**	NA	HVAS	Digitel DHA-77 (Digitel, Volketswil, CH)	PM_{10}	30	1633 [1625 - 1642]	a	3
BRM	Beromünster	A	HVAS	Digitel DHA-80 (Digitel, Volketswil, CH)	PM_{10}	30	761 [743 - 797]	c	3
CBW	Cabauw – Wielsekade	A	LVAS	LVS 3.1 (Combde-Derenda, Stahnsdorf, DE)	PM_{10}	2.3	110	b	3
CRA	Centre de Recherches Atmosphériques	A	HVAS	Algade EAS100K (Algade, Bessines-sur-Gartempe, FR)	PM_{10}	80	3847	a	1
DBG	Integrated Monitoring Station Puszcza Borecka, Diabla Gora	NA	HVAS	Digitel DHA-80 (Digitel, Volketswil, CH)	PM_{10}	30	1587 [1572 - 1607]	a	3
HTM	Hyltemossa	NA	HVAS	Dekati (Dekati, Kangasala, FI)	TSP	60	2880	a	3
HYY	Station for Measuring Ecosystem - Atmosphere Relations (SMEARII), Hyytiälä	NA	LVAS	Dekati PM10 (Dekati, Kangasala, FI)	PM_{10}	1.4	66 [65 - 67]	b	3
**KOS**	**National Atmospheric Observatory Košetice**	A	LVAS	Leckel (Leckel, Berlin, Germany) / Tisch Environmental, Cleves, USA)	PM_{10}	2.3	110	a	3
MEL	TROPOS research station Melpitz	A	HVAS	Digitel DHA-80 (Digitel, Volketswil, CH)	PM_{10}	30	1435 [1403 - 1469]	a	3
MSY	Montseny	NA	HVAS	Digitel DH-80 (Digitel, Volketswil, Switzerland)	PM_{10}	30	1465 [1459 - 1469]	a	3
OPE	Observatoire Pérenne de l'Environnement	A	HVAS	Digitel DA-80 (Digitel, Volketswil, CH)	PM_{10}	30	1352 [1345 - 1364]	a	3
PAY	Payerne	NA	HVAS	Digitel DHA-80 (Digitel, Volketswil, CH)	PM_{10}	30	1459 [1452 - 1463]	c	3
RIG	Rigi	NA	HVAS	Digitel DHA-80 (Digitel, Volketswil, CH)	PM_{10}	30	1451 [1445 - 1455]	c	3
SME	SMEAR Estonia	NA	LVAS	Custom made	TSP	1.9	92 [83 - 97]	a	3
THK	Tahkuse	NA	LVAS	Custom made	TSP	1.8	86 [84 - 87]	a	3

a = 150 mm (Whatman, Maidstone, UK); b = 47 mm (Whatman, Maidstone, UK); c = PALLFLEX Tissuquartz 2500 QAT-UP (Pall, New-York, USA); HVAS = high-volume air sampler; LVAS = low-volume air sampler; PM_{10} = particulate matter with an aerodynamic diameter <10 µm; TSP = total suspended particles; A = agriculture adjacent; NA= non-agriculture adjacent

(B)

Site code	Sampling site	Sampler type	Sampler used	Inlet particle size fraction	Flow-rate (m^3 h^{-1})	Sampled volume (m^3) Average [Min-Max]	Filter QFF	Number of samples collected
ARN	El Arenosillo	HVAS	MCV CAV-A (MCV, SA, Barcelona, ES)	PM_{10}	30/30	1465 [1459 - 1469]	b	3
FKL	Finokalia Station	HVAS	TE 5170 (Tisch Environmental, Cleves, USA)	TSP	49.8	3918 [3850 - 3986]	d	2
MHD	Mace Head	HVAS	Sierra Andersen Impactor (USA)	TSP	97.2	4665	d, e	1
WEY	Weybourne Atmospheric Observatory	HVAS	TE 5170 (Tisch Environmental, Cleves, USA)	TSP	66	3168	d	3

a = 150 mm (Whatman, Maidstone, UK); b = 47 mm (Whatman, Maidstone, UK); c = 203 × 254 mm (Whatman, Maidstone, UK); d = slotted filters (Whatman, Maidstone, UK); HVAS = high-volume air sampler; PM_{10} = particulate matter with an aerodynamic diameter <10 µm; TSP = total suspended particles

(C)

Site code	Sampling site	Sampler type	Sampler used	Inlet Particle size fraction	Flow-rate (m^3 h^{-1})	Sampled volume (m^3) Average [Min-Max]	Filter QFF	Number of samples collected
CPK	Chopok	LVAS	Custom made	PM_{10}	0.9	31 [30 - 32]	b	2
HAC	Helmos Mt. Hellenic Atmospheric Aerosol and Climate Change station	HVAS	Digitel DHA-80 (Digitel, Volketswil, CH)	TSP	30	1627 [1623 - 1632]	a	2
JFJ	Jungfraujoch	HVAS	Digitel DHA-80 (Digitel, Volketswil, CH)	PM_{10}	45	2175 [2166 - 2187)	c	3
PDM	Pic du Midi de Bigorre	LVAS	Custom made	PM_{10}	2.8	133	b	1
**SBO**	**Sonnblick Observatory**	HVAS	Digitel DHA-80 (Digitel, Volketswil, CH)	PM_{10}	3	323	a	2
**UFS**	**Environmental Research Station Schneefernerhaus, Mt. Zugspitze**	HVAS/LVAS	Digitel DHA-80 (Digitel, Volketswil, CH) / Digitel-Kroneis-Genius5 hybrid, CH	PM_{10} /PM_{10}	30 /7.2	1401 [1394 - 1408] / 396 [395-397]	a/a	2/3

a = 150 mm (Whatman, Maidstone, UK); b = 47 mm (Whatman, Maidstone, UK); c = PALLFLEX Tissuquartz 2500 QAT-UP (Pall, New-York, USA); HVAS = high-volume air sampler; LVAS = low-volume air sampler; PM10 = particulate matter with an aerodynamic diameter <10 µm; TSP = total suspended particles

(D)

Site code	Sampling site	Sampler type	Sampler used	Inlet particle size fraction	Flow-rate ($m^3 h^{-1}$)	Sampled volume (m^3) Average [Min-Max]	Filter QFF	Number of samples collected
**ADA**	**Andøya**	HVAS	Digitel DHA-77 (Digitel, Volketswil, CH)	PM_{10}	30	1652 [1645 - 1656]	a	3
PAL	Pallas Atmosphere-Ecosystem Supersite	LVAS	LVS 3.1 (Combde-Derenda, Stahnsdorf, DE)	PM_{10}	1	47 [45 - 48]	b	3
**ZPO**	**Zeppelin Observatory**	HVAS	Digitel DHA-77 (Digitel, Volketswil, CH)	TSP	30	1357	a	3

a = 150 mm (Whatman, Maidstone, UK); b = 47 mm (Whatman, Maidstone, UK); HVAS = high-volume air sampler; LVAS = low-volume air sampler; PM_{10} = particulate matter with an aerodynamic diameter <10 µm; TSP = total suspended particles

Table S2. Types of land-use within a 10 km radius for the rural, adjacent (A) and non-adjacent (B) to agricultural land, coastal (C), mountain (D) and polar (E) sites

(A)

	BRM	CBW	CRA	KOS	MEL	OPE	PAY
Artificial surface with possible pesticides uses	11	9	4	3	5	<1	9
Artificial surface without possible pesticides uses	<1	1	1	0	2	<1	1
Agricultural areas	64	87	50	65	55	48	63
Forest	15	0	31	29	23	39	17
Shrub and/or herbaceous vegetation associations	2	<1	14	3	14	12	7
Open spaces with little or no vegetation	0	0	0	0	0	0	0
Wetlands	0	1	0	0	0	0	<1
Water bodies	8	2	0	0	2	0	3

(B)

	AMO	BKO	DBG	HYL	HYY	MSY	RIG	SME	THK
Artificial surface with possible pesticides uses	3	<1	1	2	<1	2	10	<1	<1
Artificial surface without possible pesticides uses	1	<1	0	<1	<1	<1	1	0	<1
Agricultural areas	12	2	37	14	5	3	20	16	35
Forest	8	88	52	75	81	86	26	52	38
Shrub and/or herbaceous vegetation associations	57	3	3	5	9	9	16	14	17
Open spaces with little or no vegetation	0	0	0	0	0	0	0	0	0
Wetlands	17	2	<1	2	2	0	1	11	9
Water bodies	1	4	7	2	2	0	26	7	0

(C)

	ARN	FKL	MHD	WEY
Artificial surface with possible pesticides uses	<1	<1	<1	3
Artificial surface without possible pesticides uses	<1	<1	0	0
Agricultural areas	0	15	9	36
Forest	7	<1	<1	6
Shrub and/or herbaceous vegetation associations	39	31	<1	4
Open spaces with little or no vegetation	19	0	4	0
Wetlands	0	0	17	4
Water bodies	35	53	69	46

(D)

	CPK	HAC	JFJ	PDM	SBO	UFS
Artificial surface with possible pesticides uses	3	<1	1	0	<1	3
Artificial surface without possible pesticides uses	0	0	0	0	<1	<1
Agricultural areas	<1	11	<1	2	<1	<1
Forest	65	44	12	19	14	49
Shrub and/or herbaceous vegetation associations	31	38	20	49	50	21
Open spaces with little or no vegetation	<1	7	67	30	34	25
Wetlands	0	0	0	0	0	<1
Water bodies	0	<1	0	<1	<1	1

(E)

	ADA	PAL	ZPO
Artificial surface with possible pesticides uses	<1	0	0
Artificial surface without possible pesticides uses	1	0	0
Agricultural areas	2	0	0
Forest	4	71	0
Shrub and/or herbaceous vegetation associations	12	7	8
Open spaces with little or no vegetation	3	2	38
Wetlands	19	10	0
Water bodies	59	11	55

Table S3. Allocation of this study land use categories to Corine Land Cover (A) and Global Land Cover (B) categories

(A)

This study's land-use categories	Corine Land Cover 2018 nomenclature
Artificial surface with possible pesticides uses	discontinuous urban fabric; roads and rail networks and associated land; green urban areas; sport and leisure facilities
Artificial surface without possible pesticides uses	continuous urban fabric; industrial or commercial units; port areas; airports; mineral extraction sites; dump sites; construction sites
Agricultural areas	non-irrigated arable land; permanently irrigated land; rice fields; vineyards; fruit trees and berry plantations; olive groves; pastures; annual crops associated with permanent crops; complex cultivation patterns; land principally occupied by agriculture, with significant areas of natural vegetation; agro-forestry areas
Forest	broad-leaved forest; coniferous forest; mixed forest
Shrub and/or herbaceous vegetation associations	natural grasslands; moors and heathland; sclerophyllous vegetation; transitional woodland-shrub
Open spaces with little or no vegetation	beaches, dunes, sands; bare rocks, sparsely vegetated areas; burnt areas; glaciers and perpetual snow
Wetlands	industrial or commercial units; peat bogs; salt marshes; salines; interdital flats; unclassified land surface
Water bodies	water courses; water bodies; coastal lagoons; estuaries; sea and ocean; unclassified water bodies

(B)

This study's land-use categories	Global Land Cover 2000
Artificial surface without possible pesticides uses	artificial surfaces and associated areas
Agricultural areas	cropland/tree cover/other natural vegetation; cropland/shrub and/or herbaceous cover
Forest	tree cover, broadleaved, evergreen; tree cover, broadleaved, deciduous, closed; tree cover, broadleaved, deciduous, open; tree cover, needle-leaved, evergreen; tree cover, needle-leaved, deciduous; tree cover, mixed leaf type; tree cover, regularly flooded, fresh water (and brackish); tree cover, regularly flooded, saline water; tree cover/other natural vegetation; tree cover, burnt
Shrub and/or herbaceous vegetation associations	shrub cover, closed-open, evergreen; shrub cover, closed-open, deciduous; herbaceous cover, closed-open; sparse herbaceous or sparse shrub cover; regularly flooded shrub and/or herbaceous cover
Open spaces with little or no vegetation	bare areas; snow and ice (natural & artificial)
Water bodies	water bodies (natural & artificial)

Table S4. Information on the chemical analysis performed and standards used for individual herbicides (A), insecticides (B) and fungicides (C)

(A)

Pesticide	Instrument	Native standard used	Internal standard
2,4-D	HPLC-MS/MS[a]	Neochema (Germany)	2,4-D - 13C6[d]
2,4-MCPA	UPLC-MS/MS[c]	Neochema (Germany)	TPP[k]
Acetochlor	HPLC-MS/MS[a]	Neochema (Germany)	Acetochlor-D11[e]
Aclonifen	GC-MS/MS[b]	None	Prosulfocarb-D7[g]
Alachlor	HPLC-MS/MS[a]	Neochema (Germany)	Alachlor-D13[e]
Atrazine	HPLC-MS/MS[a]	Chromservis (Czech Republic)	Atrazine-D5[h]
Chloridazon	HPLC-MS/MS[a]	Chromservis (Czech Republic)	Pyrazon-D5[e]
Chlorotoluron	HPLC-MS/MS[a]	Neochema (Germany)	Chlortoluron-D6[e]
Chlorpropham	GC-MS/MS[b]	None	Prosulfocarb-D7[g]
Chlorsulfuron	HPLC-MS/MS[a]	Neochema (Germany)	Metamitron-D5[f]
Clomazone	GC-MS/MS[b]	None	Prosulfocarb-D7[g]
Diclofop-methyl	GC-MS/MS[b]	None	Prosulfocarb-D7[g]
Diflufenican	GC-MS/MS[b]	None	Boscalid-D4[d]
Dimethachlor	HPLC-MS/MS[a]	Chromservis (Czech Republic)	Diuron-D6[h]
Dimethenamid-P	GC-MS/MS[b]	None	Kresoxim-methyl-D7[d]
Diuron	HPLC-MS/MS[a]	Chromservis (Czech Republic)	Diuron-D6[h]
Flumioxazin	GC-MS/MS[b]	None	Boscalid-D4[d]
Flurochloridone	GC-MS/MS[b]	None	Chlorpyrifos methyl-D6[d]
Fluroxypyr	HPLC-MS/MS[a]	Neochema (Germany)	Metribuzin-D3[f]
Isoproturon	HPLC-MS/MS[a]	Chromservis (Czech Republic)	Isoproturon-D3[f]
Lenacil	GC-MS/MS[b]	None	Prosulfocarb-D7[g]
Linuron	GC-MS/MS[b]	None	Prosulfocarb-D7[g]
Mecoprop	HPLC-MS/MS[a]	Neochema (Germany)	Mecoprop-D6[f]
Metamitron	HPLC-MS/MS[a]	Chromservis (Czech Republic)	Metamitron-D5[f]
Metazachlor	HPLC-MS/MS[a]	Neochema (Germany)	Metazachlor-D6[i]
Metribuzin	HPLC-MS/MS[a]	Chromservis (Czech Republic)	Metribuzin-D3[f]
Oxadiazon	HPLC-MS/MS[a]	Neochema (Germany)	Aldicarb-D3[d]
Pendimethalin	HPLC-MS/MS[a]	Chromservis (Czech Republic)	Pendimethalin-D5[f]
Prosulfocarb	HPLC-MS/MS[a]	Neochema (Germany)	Prosulfocarb-D7[g]
Quizalofop ethyl	HPLC-MS/MS[a]	Neochema (Germany)	Quizalofop ethyl-D3[i]
Simazine	HPLC-MS/MS[a]	Chromservis (Czech Republic)	Simazine-D10[f]
S-Metolachlor	HPLC-MS/MS[a]	Neochema (Germany)	Metolachlor-D6[e]
Sulcotrione	UPLC-MS/MS[c]	None	TPP[k]
Terbuthylazine	HPLC-MS/MS[a]	Chromservis (Czech Republic)	Terbuthylazine-D5[e]
Triallate	GC-MS/MS[b]	Neochema (Germany)	Triallate-13C6[j]

[a] Agilent 1290 (Agilent Technologies, Palo, Alto, California, USA), Mass spectrometer: QTRAP 5500 (AB Sciex, Foster City, California, USA); [b] Trace GC Ultra (Thermo Scientific, Waltham, MA, USA), Mass spectrometer: TSQ QuantumTM Triple Quadrupole (Thermo Scientific, Waltham, Massachussets, USA); [c] Acquity (Waters, Milford, Massachussets, USA), Mass spectrometer: Synapt G2 HDMS (Waters, Milford, Massachussets, USA); [d] Toronto Research Chemicals Inc. (Canada); [e] LGC Dr. Ehrenstorfer (Germany); [f] HPC Standards GmbH (Germany); [g] ASCA GmbH (Germany); [h] Restek (United States); [i] Chiron AS (Norway); [j] Cambridge Isotope Laboratories, Inc. (United States); [k] Sigma-Aldrich (United States); HPLC = High performance liquid chromatography; UPLC = Ultra-performance liquid chromatography; GC = Gas chromatography; MS/MS = Tandem mass spectrometer; TPP = Triphenyl phosphate

(B)

Pesticide	Instrument	Native standard used	Internal standard
Acetamiprid	HPLC-MS/MS[a]	Neochema (Germany)	Acetamiprid-D3[e]
Azinphos methyl	HPLC-MS/MS[a]	Chromservis (Czech Republic)	Fenitrothion-D6[d]
Carbaryl	HPLC-MS/MS[a]	Neochema (Germany)	Diuron-D6[g]
Carbofuran	HPLC-MS/MS[a]	Neochema (Germany)	Carbofuran-D3[c]
Chlorpyrifos	HPLC-MS/MS[a]	Chromservis (Czech Republic)	Chlorpyrifos-D10[d]
Cypermethrin	GC-MS/MS[b]	Neochema (Germany)	Permethrin-D5[c]
Diazinon	HPLC-MS/MS[a]	Chromservis (Czech Republic)	Diuron-D6[g]
Dimethoate	HPLC-MS/MS[a]	Neochema (Germany)	Dimethoate-D6[e]
Disulfoton	HPLC-MS/MS[a]	Neochema (Germany)	Chlorpyrifos-D10[d]
Esbiothrin	GC-MS/MS[b]	None	Prosulfocarb-D7[f]
Fenitrothion	HPLC-MS/MS[a]	Neochema (Germany)	Fenitrothion-D6[d]
Fenoxycarb	GC-MS/MS[b]	None	Prosulfocarb-D7[f]
Fipronil	GC-MS/MS[b]	None	Prosulfocarb-D7[f]
Fonofos	HPLC-MS/MS[a]	Chromservis (Czech Republic)	Diuron-D6[g]
Malathion	HPLC-MS/MS[a]	Neochema (Germany)	Simazine-D10[e]
Parathion methyl	HPLC-MS/MS[a]	Chromservis (Czech Republic)	Fenitrothion-D6[d]
Permethrin	GC-MS/MS[b]	Neochema (Germany)	Permethrin-D5[c]
Phosalone	HPLC-MS/MS[a]	Neochema (Germany)	Phosalone-D10[d]
Pirimicarb	HPLC-MS/MS[a]	Neochema (Germany)	Diuron-D6[g]
Temephos	HPLC-MS/MS[a]	Neochema (Germany)	Chlorpyrifos-D10[d]
Terbufos	HPLC-MS/MS[a]	Neochema (Germany)	Diuron-D6[g]
Thiacloprid	HPLC-MS/MS[a]	Neochema (Germany)	Thiacloprid-D4[c]

[a] Agilent 1290 (Agilent Technologies, Palo, Alto, California, USA), Mass spectrometer: QTRAP 5500 (AB Sciex, Foster City, California, USA); [b] Trace GC Ultra (Thermo Scientific, Waltham, MA, USA), Mass spectrometer: TSQ QuantumTM Triple Quadrupole (Thermo Scientific, Waltham, Massachussets, USA); [c] Toronto Research Chemicals Inc. (Canada); [d] LGC Dr. Ehrenstorfer (Germany); [e] HPC Standards GmbH (Germany); [f] ASCA GmbH (Germany); [g] Restek (United States); HPLC = High performance liquid chromatography; GC = Gas chromatography; MS/MS = Tandem mass spectrometer

(C)

Pesticide	Instrument	Native standard used	Internal standard
Boscalid	HPLC-MS/MS[a]	Neochema (Germany)	Boscalid-D4[d]
Carbendazim	HPLC-MS/MS[a]	Neochema (Germany)	Carbendazim-D4[f]
Cyprodinil	HPLC-MS/MS[a]	Neochema (Germany)	Cyprodinil-D5[d]
Difenoconazole	GC-MS/MS[b]	None	Prosulfocarb-D7[g]
Dimethomorph	GC-MS/MS[b]	None	Permethrin-D5[d]
Epoxiconazole	GC-MS/MS[b]	None	Kresoxim-methyl-D7[d]
Fenpropidin	HPLC-MS/MS[a]	Neochema (Germany)	Fenpropidin-D10[d]
Fenpropimorph	HPLC-MS/MS[a]	Chromservis (Czech Republic)	Diuron-D6[h]
Fluazinam	UPLC-MS/MS[c]	None	TPP[i]
Flusilazole	GC-MS/MS[b]	None	Prosulfocarb-D7[g]
Iprovalicarb	HPLC-MS/MS[a]	Neochema (Germany)	Aldicarb-D3[d]
Kresoxim-methyl	HPLC-MS/MS[a]	Neochema (Germany)	Kresoxim-methyl-D7[d]
Metalaxyl	HPLC-MS/MS[a]	Neochema (Germany)	Metalaxyl-D6[d]
Prochloraz	HPLC-MS/MS[a]	Chromservis (Czech Republic)	Prochloraz-D7[e]
Propiconazole	HPLC-MS/MS[a]	Neochema (Germany)	Propiconazole-D5[e]
Pyrimethanil	GC-MS/MS[b]	None	Boscalid-D4[d]
Spiroxamine	HPLC-MS/MS[a]	Neochema (Germany)	Spiroxamine-D4[d]
Tebuconazole	HPLC-MS/MS[a]	Chromservis (Czech Republic)	Tebuconazole-D6[e]
Tetraconazole	GC-MS/MS[b]	None	Prosulfocarb-D7[g]

[a] Agilent 1290 (Agilent Technologies, Palo, Alto, California, USA), Mass spectrometer: QTRAP 5500 (AB Sciex, Foster City, California, USA); [b] Trace GC Ultra (Thermo Scientific, Waltham, MA, USA), Mass spectrometer: TSQ QuantumTM Triple Quadrupole (Thermo Scientific, Waltham, Massachussets, USA); [c] Acquity (Waters, Milford, Massachussets, USA), Mass spectrometer: Synapt G2 HDMS (Waters, Milford, Massachussets, USA); [d] Toronto Research Chemicals Inc. (Canada); [e] LGC Dr. Ehrenstorfer (Germany); [f] HPC Standards GmbH (Germany); [g] ASCA GmbH (Germany); [h] Restek (United States); [i] Sigma-Aldrich (United States); HPLC = High performance liquid chromatography; UPLC = Ultra-performance liquid chromatography; GC = Gas chromatography; MS/MS = Tandem mass spectrometer; TPP = Triphenyl phosphate

Table S5. Information on the individual herbicides (A), insecticides (B) and fungicides (C) analysed in this study

(A)

Pesticide	CAS number[57]	Chemical class[57]	Status under Reg. (EC) No 1107/2009[75]	Date of approval[75]	Expiration of approval[75]	Countries in which the pesticide is or lately was authorized[75]	Biocide[55]	Used in the top 20 crops[72]	Priority active ingredient in France[73]	Highly hazardous pesticide[74]	High risk pesticide[74]
2,4-D	94-75-7 4	Alkychlorophenoxy	Approved	01/01/2016	31/12/2030	AT, BE, BG, CY, CZ, DE, DK, EE, EL, ES, FI, FR, HR, HU, IE, IT, LT, LU, LV, MT, NL, PL, PT, RO, SE, SI, SK, UK		Yes	Yes		
2,4-MCPA	94-74-6	Aryloxyalkanoic acid	Approved	01/05/2006	31/10/2022	AT, BE, BG, CY, CZ, DE, DK, EE, EL, ES, FI, FR, HR, HU, IE, LT, LU, LV, MT, NL, PL, PT, RO, SE, SI, SK					
Acetochlor	34256-82-1	Chloroacetamide	Not approved					Yes	Yes		
Aclonifen	74070-46-5	Diphenyl ether	Approved	01/08/2009	31/07/2022	AT, BE, BG, CY, CZ, DE, DK, EE, EL, ES, FI, FR, HR, HU, IE, IT, LT, LU, LV, NL, PL, PT, RO, SE, SI, SK, UK					
Alachlor	15972-60-8	Chloroacetamide	Not approved					Yes		Yes	
Atrazine	1912-24-9	Triazine	Not approved					Yes		Yes	
Chloridazon	1698-60-8	Pyridazinone	Not approved		31/12/2018	HU, NL, PL, UK					
Chlorotoluron	15545-48-9	Urea	Not approved	01/01/2010	31/12/2019						
Chlorpropham	101-21-3	Carbamate	Approved	01/03/2006	31/10/2022	AT, BE, BG, CZ, DE, EE, EL, ES, FR, HR, HU, IE, LV, PL, PT, RO, SI, SK, UK			Yes		
Chlorsulfuron	15545-48-9	Urea	Not approved	01/01/2010	31/12/2019						
Clomazone	81777-89-1	Isoxazolidinone	Approved	01/11/2008	31/10/2022	AT, BE, BG, CY, CZ, DE, DK, EE, EL, ES, FR, HR, HU, IE, LT, LU, LV, MT, NL, PL, PT, RO, SE, SI, SK, UK		Yes	Yes		
Diclofop-methyl	51338-27-3	Aryloxyphenoxypropionate	Approved	01/06/2011	31/05/2023	El, ES, PT					Yes
Diflufenican	83164-33-4	Carboxamide	Approved	01/01/2009	31/12/2022	AT, BE, BG, CY, CZ, DE, DK, EE, EL, ES, FI, FR, HR, HU, IE, LT, LU, LV, MT, NL, PL, PT, RO, SE, SI, SK, UK			Yes		
Dimethachlor	50563-36-5	Chloroacetamide	Approved	01/01/2010	31/12/2022	AT, BG, CZ, DE, EE, FR, HR, HU, IE, LT, LV, PL, RO, SI, SK, UK					

Table S5. Information on the individual herbicides (A), insecticides (B) and fungicides (C) analysed in this study (continued)

(A)

**Pesticide**	**CAS number[57]**	**Chemical class[57]**	**Status under Reg. (EC) No 1107/2009[75]**	**Date of approval[75]**	**Expiration of approval[75]**	**Countries in which the pesticide is or lately was authorized[75]**	**Biocide[55]**	**Used in the top 20 crops[72]**	**Priority active ingredient in France[73]**	**Highly hazardous pesticide[74]**	**High risk pesticide[74]**
Dimethenamid-P	163515-14-8	Chloroacetamide	Approved	01/09/2019	31/08/2034	AT, BE, BG, CY, CZ, DE, EL, ES, FI, FR, HR, HU, IE, LT, LU, LV, NL, PL, PT, RO, SE, SI, SK, UK		Yes	Yes		Yes
Diuron	330-54-1	Phenylamide	Not approved	01/10/2008	30/09/2020	IT		Yes	Yes		
Flumioxazin	103361-09-7	N-phenylphtalamides	Approved	01/01/2003	28/02/2037	AT, BE, BG, CZ, DE, EL, ES, HR, HU, IE, LV, NL, RO, UK		Yes		Yes	
Flurochloridone	61213-25-0	Pyrrolidine	Approved	01/06/2011	31/05/2022	CZ, EL, FR, HR, HU, IT, PL, RO, SK					
Fluroxypyr	69377-81-7	Pyridine	Approved	01/01/2012	31/12/2024	AT, BE, BG, CY, CZ, DE, DK, EE, EL, ES, FI, FR, HR, HU, IE, LT, LU, LV, MT, NL, PL, PT, RO, SE, SI, SK, UK		Yes			
Isoproturon	34123-59-6	Urea	Not approved		30/06/2016	IT, LT, NL					
Lenacil	01/08/2164	Uracil	Approved	01/01/2009	31/12/2022	AT, BE, CY, CZ, DE, EL, ES, FR, HU, IE, NL, PL, PT, RO, SK, UK			Yes		Yes
Linuron	330-55-2	Urea	Not approved	01/01/2004	31/07/2017				Yes		
Mecoprop	7085-19-0	Aryloxyalkanoic acid	Not approved	01/06/2004	31/01/2017	IT					
Metamitron	41394-05-2	Triazinone	Approved	01/09/2009	31/08/2022	AT, BE, CY, CZ, DE, DK, EE, EL, ES, FI, FR, HR, HU, IE, IT, LT, LU, LV, MT, NL, PL, PT, RO, SE, SI, SK, UK			Yes		
Metazachlor	67129-08-2	Chloroacetamide	Approved	01/08/2009	31/07/2022	AT, BE, BG, CY, CZ, DE, EE, EL, ES, FI, FR, HR, HU, IE, IT, LT, LU, LV, MT, NL, PL, PT, RO, SI, SK, UK			Yes		
Metribuzin	21087-64-9	Triazinone	Approved	01/10/2007	31/07/2022	AT, BE, BG, CY, CZ, DE, EE, EL, ES, FI, FR, HR, HU, IE, IT, LU, LV, MT, NL, PL, PT, RO, SE, SI, SK, UK		Yes	Yes		Yes
Oxadiazon	19666-30-9	Oxidiazole	Not approved		31/12/2018				Yes		Yes
Pendimethalin	40487-42-1	Dinitroaniline	Approved	01/09/2017	30/11/2024	AT, BE, BG, CY, CZ, DE, DK, EE, EL, ES, FI, FR, HR, HU, IE, LT, LU, LV, MT, NL, PL, PT, RO, SI, SK, UK		Yes	Yes		Yes

Table S5. Information on the individual herbicides (A), insecticides (B) and fungicides (C) analysed in this study (continued)

(A)

Pesticide	CAS number[57]	Chemical class[57]	Status under Reg. (EC) No 1107/2009[75]	Date of approval[75]	Expiration of approval[75]	Countries in which the pesticide is or lately was authorized[75]	Biocide[55]	Used in the top 20 crops[72]	Priority active ingredient in France[73]	Highly hazardous pesticide[74]	High risk pesticide[74]
Prosulfocarb	52888-80-9	Thiocarbamate	Approved	01/11/2009	31/10/2022	AT, BE, BG, CY, CZ, DE, DK, EE, EL, ES, FI, FR, HR, HU, IE, LT, LU, LV, NL, PL, PT, RO, SE, SI, SK, UK			Yes		
Quizalofop ethyl	76578-14-8	Aryloxyphenoxypropionate	Approved	01/12/2009	30/11/2022	AT, BE, BG, CY, CZ, DE, EE, EL, ES, FI, FR, HR, HU, IE, LT, LU, LV, MT, NL, PL, PT, RO, SE, SI, SK, UK					
Simazine	122-34-9	Triazine	Not approved					Yes			Yes
S-Metolachlor	87392-12-9	Chloroacetamide	Approved	01/04/2005	31/07/2022	AT, BE, BG, CY, CZ, DE, EL, ES, FR, HR, HU, IE, IT, LU, MT, NL, PL, PT, RO, SI, SK, UK		Yes	Yes		Yes
Sulcotrione	99105-77-8	Triketone	Approved	01/09/2009	31/08/2022	BE, BG, CZ, DE, EL, ES, FR, HU, LU, NL, PL, PT, RO, SK					
Terbuthylazine	5915-41-3	Triazine	Approved	01/01/2012	31/12/2024	AT, BE, BG, CY, CZ, DE, EL, ES, FR, HR, HU, IE, IT, LU, MT, NL, PL, PT, RO, SI, SK, UK					
Triallate	2303-17-5	Thiocarbamate	Approved	01/01/2010	31/12/2022	BE, FR, IE, NL, UK		Yes	Yes		Yes

EU countries code glossary: https://ec.europa.eu/eurostat/statistics-explained/index.php?title=Glossary:Country_codes

Table S5. Information on the individual herbicides (A), insecticides (B) and fungicides (C) analysed in this study (continued)

(B)

Pesticide	CAS number[57]	Chemical class[57]	Status under Reg. (EC) No 1107/2009[75]	Date of approval[75]	Expiration of approval[75]	Countries in which the pesticide is or lately was authorized[75]	Biocide[55]	Used in the top 20 crops[72]	Priority active ingredient in France[73]	Highly hazardous pesticide[74]	High risk pesticide[74]
Acetamiprid	135410-20-7	Neonicotinoid	Approved	01/03/2018	28/02/2033	AT, BE, BG, CY, CZ, DE, DK, EE, EL, ES, FI, FR, HR, HU, IE, IT, LT, LU, LV, MT, NL, PL, PT, RO, SE, SI, SK, UK	X				
Azinphos methyl	86-50-0	Organophosphate	Not approved							X	
Carbaryl	63-25-2	Carbamate	Not approved					X			X
Carbofuran	1563-66-2	Carbamate	Not approved							X	
Chlorpyrifos	2921-88-2	Organophosphate	Not approved	01/02/2005	08/07/2019	EE, UK		X	X		X
Cypermethrin	52315-07-8	Pyrethroid	Approved	01/02/2022	31/01/2029	AT, BE, BG, CY, CZ, DE, DK, EE, EL, ES, FI, FR, HR, HU, IE, LT, LU, LV, MT, NL, PL, PT, RO, SE, SI, SK, UK	X		X		X
Diazinon	333-41-5	Organophosphate	Not approved			IT					X
Dimethoate	60-51-5	Organophosphate	Not approved	01/10/2007	30/06/2019	BE, IT, UK		X	X		X
Disulfoton	298-04-4	Organophosphate	Not approved							X	
Esbiothrine	260359-57-7	Pyrethroid	Not approved								
Fenitrothion	122-14-5	Organophosphate	Not approved								X
Fenoxycarb	72490-01-8	Carbamate	Not approved	01/06/2011	31/05/2021	BE, CY, EL, ES, HR, HU, NL, PT	X				X
Fipronil	120068-37-3	Phenylpyrazole	Not approved		30/09/2017		X		X	X	
Fonofos	944-22-9	Organophosphate	Not approved			IT					
Malathion	121-75-5	Organophosphate	Approved	01/05/2010	30/04/2023	EL, IT		X			X
Parathion methyl	298-00-0	Organophosphate	Not approved							X	
Permethrin	52645-53-1	Pyrethroid	Not approved			IT	X		X		X
Phosalone	2310-17-0	Organophosphate	Not approved								X
Pirimicarb	23103-98-2	Carbamate	Approved	01/02/2007	30/04/2023	AT, BE, CZ, DE, DK, EL, ES, FR, HR, HU, IE, IT, LU, NL, PL, PT, SE, SI, SK, UK			X		X
Temephos	3383-96-8	Organophosphate	Not approved								
Terbufos	13071-79-9	Organophosphate	Not approved			IT		X		X	
Thiacloprid	111988-49-9	Neonicotinoid	Not approved	01/01/2005	03/02/2020	FI, IE, UK					X

EU countries code glossary: https://ec.europa.eu/eurostat/statistics-explained/index.php?title=Glossary:Country_codes

Table S5. Information on the individual herbicides (A), insecticides (B) and fungicides (C) analysed in this study (continued)

(C)

Pesticide	CAS number[57]	Chemical class[57]	Status under Reg. (EC) No 1107/2009[75]	Date of approval[75]	Expiration of approval[75]	Countries in which the pesticide is or lately was authorized[75]	Biocide[55]	Used in the top 20 crops[72]	Priority active ingredient in France[73]	Highly hazardous pesticide[74]	High risk pesticide[74]
Boscalid	188425-85-6	Carboxamide	Approved	01/08/2008	31/07/2022	AT, BE, BG, CY, CZ, DE, DK, EE, EL, ES, FI, FR, HR, HU, IE, IT, LT, LU, LV, MT, NL, PL, PT, RO, SE, SI, SK, UK			X		
Carbendazim	10605-21-7	Benzimidazole	Not approved		30/11/2014		X			X	
Cyprodinil	121552-61-2	Anilinopyrimidine	Approved	01/05/2007	30/04/2023	AT, BE, BG, CY, CZ, DE, DK, EE, EL, ES, FI, FR, HR, HU, IE, LT, LU, LV, MT, NL, PL, PT, RO, SE, SI, SK, UK			X		
Difenoconazole	119446-68-3	Triazole	Approved	01/01/2009	31/12/2022	AT, BE, BG, CY, CZ, DE, DK, EE, EL, ES, FI, FR, HR, HU, IE, LT, LU, LV, MT, NL, PL, PT, RO, SE, SI, SK, UK			X		
Dimethomorph	110488-70-5	Morpholine	Approved	01/10/2007	31/07/2022	AT, BE, BG, CY, CZ, DE, DK, EE, EL, ES, FI, FR, HR, HU, IE, LT, LU, LV, MT, NL, PL, PT, RO, SE, SI, SK, UK					
Epoxiconazole	133855-98-8	Triazole	Not approved	01/05/2009	30/04/2020	HU, IE, NL, PL, PT, UK			X	X	
Fenpropidin	67306-00-7	Morpholine	Approved	01/01/2009	31/12/2022	AT, BE, BG, CZ, DE, EE, EL, ES, FR, HU, IE, IT, LT, LV, NL, PL, PT, RO, SE, SI, SK, UK			X		
Fenpropimorph	67564-91-4	Morpholine	Not approved	01/05/2009	30/04/2019	IT, NL, UK	X				
Fluazinam	79622-59-6	Phenylpyridinamine	Approved	01/03/2009	28/02/2023	AT, BE, BG, CY, CZ, DE, DK, EE, EL, ES, FI, FR, HR, HU, IE, LT, LU, LV, MT, NL, PL, PT, RO, SE, SI, SK, UK			X		X
Flusilazole	85509-19-9	Triazole	Not approved		30/06/2008						
Iprovalicarb	140923-17-7	Carbamate	Approved	01/04/2016	31/03/2031	AT, BG, CY, CZ, DE, EL, FR, HR, HU, PT, RO, SI, SK					
Kresoxim-methyl	143390-89-0	Strobilurin	Approved	01/01/2012	31/12/2024	AT, BE, BG, CY, CZ, DE, DK, EE, EL, ES, FI, FR, HR, HU, IE, LT, LU, LV, NL, PL, PT, RO, SE, SI, SK, UK					
Metalaxyl	57837-19-1	Phenylamide	Approved	01/07/2010	30/06/2023	AT, BE, BG, CY, CZ, DE, EL, ES, FR, HR, HU, IT, MT, PL, PT, RO, SI, SK					X

Table S5. Information on the individual herbicides (A), insecticides (B) and fungicides (C) analysed in this study (Continued)

(C)

Pesticide	CAS number[57]	Chemical class[57]	Status under Reg. (EC) No 1107/2009[75]	Date of approval[75]	Expiration of approval[75]	Countries in which the pesticide is or lately was authorized[75]	Biocide[55]	Used in the top 20 crops[72]	Priority active ingredient in France[73]	Highly hazardous pesticide[74]	High risk pesticide[74]
Prochloraz	67747-09-5	Imidazole	Not approved	01/01/2012	31/12/2021	BE, CY, CZ, EL, HR, HU, IE, LT, MT, PT, UK			X		
Propiconazole	60207-90-1	Triazole	Not approved	01/06/2004	19/12/2018	UK	X	X		X	
Pyrimethanil	53112-28-0	Anilinopyrimidine	Approved	01/06/2007	30/04/2023	AT, BE, BG, CY, CZ, DE, DK, EL, ES, FI, FR, HR, HU, IE, LT, LU, MT, NL, PL, PT, RO, SE, SI, SK, UK			X		
Spiroxamine	118134-30-8	Morpholine	Approved	01/01/2012	31/12/2023	AT, BE, BG, CY, CZ, DE, DK, EE, EL, ES, FI, FR, HR, HU, IE, LT, LU, LV, PL, PT, RO, SE, SI, SK, UK			X		
Tebuconazole	107534-96-3	Triazole	Approved	01/09/2009	31/08/2022	AT, BE, BG, CY, CZ, DE, DK, EE, EL, ES, FI, FR, HR, HU, IE, IT, LT, LU, LV, MT, NL, PL, PT, RO, SE, SI, SK, UK	X	X	X		
Tetraconazole	112281-77-3	Triazole	Approved	01/01/2010	31/12/2022	AT, BE, BG, CY, CZ, DE, EL, ES, FR, HR, HU, LT, MT, PL, PT, RO, SI, SK, UK					X

EU countries code glossary: https://ec.europa.eu/eurostat/statistics-explained/index.php?title=Glossary:Country_codes

Table S6. Concentrations of individual pesticides (in pg m^{-3}) observed in field blanks (FB, QFFs) for the rural (A), coastal (B), mountain and polar (C) sites. The average sampled volume at each site was used to estimate the concentrations. The cells in blue and orange represent results lower than the instrumental limit of detection (iLOD) and the instrumental limit of quantifications (iLOQ), respectively.

(A)

Analytes	FB-AMO	FB-BKO	FB-BRM	FB-CBW	FB-CRA	FB-DBG	FB-HTM	FB-HYY	FB-KOS	FB-MEL	FB-MSY	FB-OPE	FB-PAY	FB-RIG	FB-SME	FB-THK
**2,4-D**	<iLOQ	<iLOD	0.3	<iLOD	<iLOQ	<iLOQ	<iLOD	<iLOD	<iLOD	<iLOD	<iLOQ	<iLOQ	0.2	0.2	<iLOD	<iLOD
**Carbendazim**	13.7	0.9	<iLOD	7	0.2	0.4	0.1	3.7	4.3	0.1	0.2	0.6	0.4	0.2	2.7	1.9
**Carbofuran**	<iLOD	<iLOD	<iLOQ	<iLOD	<iLOD	<iLOD	<iLOD	<iLOD	<iLOD	<iLOD	<iLOD	<iLOD	<iLOQ	<iLOD	<iLOD	<iLOD
**Chlorpyrifos**	2.5	<iLOQ	0.8	0.6	<0.1	<0.1	<iLOD	1.9	0.7	0.1	<0.1	0.1	0.3	<iLOD	<iLOQ	<iLOD
**Cyprodinil**	<iLOD	<iLOD	<iLOD	<iLOD	<iLOD	<iLOD	<iLOQ	<iLOQ	<iLOD	<iLOD	0.4	<iLOD	<iLOD	<iLOD	<iLOD	<iLOQ
**Fenpropidin**	<iLOD	<iLOD	<iLOD	<iLOD	<iLOD	<iLOD	<iLOQ	<iLOQ	<iLOD	<iLOD	0.3	<iLOD	<iLOD	<iLOD	<iLOD	<iLOD
**Fenpropimorph**	<iLOD	0.2	<iLOD	<iLOD	<iLOD	0.1	<iLOD	<iLOD	<iLOD	<iLOD	<iLOQ	<iLOD	<iLOD	<iLOD	<iLOD	<iLOD
**Iprovalicarb**	<iLOD	<iLOD	9.5	<iLOD	<iLOD	<iLOD	<iLOD	<iLOD	<iLOD	<iLOD	<iLOD	<iLOD	9.2	<iLOD	<iLOD	<iLOD
**Mecoprop**	<iLOD	<iLOD	0.3	<iLOD	<iLOD	<iLOD	<iLOD	<iLOD	<iLOD	<iLOD	0.1	<iLOD	0.6	<iLOD	<iLOD	<iLOD
**Pendimethalin**	1.7	<iLOQ	1.9	<iLOQ	<0.1	0.1	<0.1	1.2	1.8	0.9	0.1	0.1	0.8	0.4	1.1	<iLOQ
**Phosalone**	<iLOD	<iLOQ	<iLOD	<iLOD	<iLOQ	<iLOD	<iLOD	<iLOD	<iLOD	<iLOQ	<iLOD	<iLOD	<iLOD	<iLOD	<iLOD	<iLOD
**Prochloraz**	<iLOD	<iLOQ	<iLOD	<iLOD	<iLOD	<iLOD	<iLOD	<iLOD	<iLOD	<iLOD	<iLOD	<iLOD	<iLOD	<iLOD	<iLOD	<iLOD
**Propiconazole**	<iLOD	<iLOD	<iLOD	<iLOD	<iLOD	<iLOD	<iLOD	<iLOD	<iLOD	<iLOD	<iLOD	<iLOD	<iLOD	<iLOD	<iLOQ	<iLOD
**Prosulfocarb**	<iLOQ	<iLOD	1.1	<iLOD	<iLOQ	<iLOD	<iLOD	<iLOD	2.5	0.2	<iLOD	<iLOD	0.5	0.6	<iLOD	<iLOD
**Quizalofop ethyl**	<iLOQ	<iLOD	<iLOD	<iLOD	<iLOD	<iLOD	<iLOD	<iLOD	<iLOD	<iLOD	<iLOD	<iLOQ	<iLOD	<iLOD	<iLOD	<iLOD
**S-Metolachlor**	<iLOD	<iLOD	3.3	<iLOD	<iLOD	<iLOD	<iLOD	<iLOD	<iLOD	<iLOD	<iLOD	<iLOD	7.4	1.9	<iLOD	<iLOD
**Tebuconazole**	<iLOD	<iLOD	<iLOD	<iLOQ	<0.1	<0.1	<iLOD	<iLOD	<iLOD	<iLOD	<0.1	<0.1	<iLOD	<iLOD	1.1	<iLOQ
**Terbuthylazine**	<iLOD	<iLOD	1.7	<iLOD	<iLOD	<iLOQ	<iLOD	<iLOD	0.5	0.3	<0.1	<iLOD	1.8	0.5	<iLOD	<iLOD

(B)

Analytes	FB-ARN	FB-FKL	FB-MHD	FB-WEY
**2,4-D**	0.1	0.1	0.3	0.4
**Acetochlor**	<iLOD	<iLOD	<iLOD	0.7
**Carbendazim**	0.5	0.2	0.1	0.2
**Chlorpyrifos**	<iLOQ	0.1	0.1	<iLOD
**Fenpropimorph**	<iLOD	<iLOD	<0.1	0.2
**Mecoprop**	<iLOD	<iLOD	0.2	0.1
**Metalaxyl**	<iLOD	<iLOD	<iLOD	<iLOQ
**Metazachlor**	<iLOD	<iLOD	<iLOD	<0.1
**Oxadiazon**	<iLOD	<iLOD	<iLOQ	<iLOQ
**Pendimethalin**	0.1	0.1	<0.1	0.1
**Pirimicarb**	<iLOD	<iLOD	<0.1	<0.1
**Propiconazole**	<iLOD	<iLOD	<iLOQ	0.3
**Prosulfocarb**	<iLOD	0.2	0.8	1.1
**S-Metolachlor**	<iLOD	<iLOD	<iLOD	0.3
**Tebuconazole**	<iLOQ	<iLOQ	<0.1	<0.1
**Terbuthylazine**	<iLOD	<iLOD	<0.1	0.1
**Thiacloprid**	<iLOD	<iLOD	<iLOD	<iLOQ

(C)

Analytes	FB-ADA	FB-CPK	FB-HAC	FB-JFJ	FB-PAL	FB-PDM	FB-SBO	FB-UFS	FB-ZPO
**2,4-D**	<iLOD	<iLOQ	<iLOQ	<iLOD	<iLOQ	<iLOD	<iLOD	<iLOD	<iLOD
**Acetochlor**	<iLOD	<iLOD	<iLOQ	<iLOD	<iLOD	<iLOD	1.3	<iLOD	<iLOD
**Carbendazim**	0.6	19.2	0.5	0.6	4.7	1.3	5.7	0.7	4.0
**Carbofuran**	<iLOD	<iLOD	<iLOD	<iLOD	<iLOD	<iLOD	<iLOD	<iLOD	<iLOD
**Chlorpyrifos**	0.0	3.2	3.6	0.1	<iLOQ	<iLOQ	0.3	<iLOQ	<iLOQ
**Cyprodinil**	<iLOD	<iLOD	<iLOD	<iLOD	<iLOD	<iLOQ	<iLOD	<iLOD	<iLOD
**Fenpropidin**	<iLOD	<iLOD	<iLOQ	<iLOD	<iLOD	<iLOD	<iLOD	<iLOD	<iLOD
**Fenpropimorph**	<iLOD	<iLOD	<iLOD	<iLOD	<iLOD	<iLOD	<iLOD	<iLOD	0.1
**Iprovalicarb**	<iLOD	<iLOD	<iLOD	<iLOD	<iLOD	<iLOD	24.5	<iLOD	<iLOD
**Mecoprop**	<iLOD	<iLOD	<iLOD	<iLOD	<iLOD	<iLOD	<iLOD	<iLOD	<iLOD
**Metalaxyl**	<iLOD	<iLOD	<iLOD	<iLOD	<iLOD	<iLOD	<iLOD	<iLOD	<iLOD
**Metazachlor**	<iLOD	<iLOD	<iLOD	<iLOD	<iLOD	<iLOD	<iLOD	<iLOD	<iLOD
**Oxadiazon**	<iLOD	<iLOD	<iLOD	<iLOD	<iLOD	<iLOD	<iLOQ	<iLOD	<iLOD
**Pendimethalin**	0.1	1.9	0.9	1.3	<iLOQ	<iLOQ	0.5	<iLOQ	0.2
**Phosalone**	<iLOD	<iLOD	<iLOD	<iLOD	<iLOD	<iLOD	<iLOD	<iLOD	<iLOD
**Pirimicarb**	<iLOD	<iLOD	<iLOD	<iLOD	<iLOD	<iLOD	<iLOD	<iLOD	<iLOD
**Prochloraz**	<iLOD	<iLOD	<iLOD	<iLOD	<iLOD	<iLOD	<iLOD	<iLOD	<iLOD
**Propiconazole**	<iLOD	<iLOD	<iLOD	<iLOD	<iLOD	<iLOD	<iLOQ	<iLOD	<iLOD
**Prosulfocarb**	<iLOD	<iLOD	<iLOQ	<iLOQ	5.5	<iLOD	<iLOQ	<iLOQ	<iLOD
**Quizalofop ethyl**	<iLOD	<iLOD	<iLOD	<iLOD	<iLOD	<iLOD	<iLOD	<iLOD	<iLOD
**S-Metolachlor**	<iLOD	<iLOD	<iLOD	<iLOD	<iLOD	<iLOD	<iLOD	<iLOD	<iLOD
**Tebuconazole**	<iLOD	<iLOD	<iLOD	<iLOD	<iLOD	<iLOD	<iLOD	<iLOD	<iLOD
**Terbuthylazine**	<iLOD	<iLOD	<iLOD	<iLOD	<iLOD	<iLOD	<iLOD	0.0	<iLOD
**Thiacloprid**	<iLOD	<iLOD	<iLOD	<iLOD	<iLOD	<iLOD	<iLOD	<iLOD	<iLOD

Table S7. Concentrations of individual pesticides (in pg m^{-3}) observed in field blanks (FB, PUF-XAD2-PUF sandwiches) at all sites. The average sampled volume at each site was used to estimate the concentrations. The cells in blue and orange represent results lower than the instrumental limit of detection (iLOD) and the instrumental limit of quantifications (iLOQ), respectively.

Analytes	FB-ADA	FB-BKO	FB-KOS	FB-SBO	FB-UFS	FB-ZPO
**2,4-D**	0.2	0.1	<iLOD	0.6	0.4	0.2
**Carbendazim**	1.2	2.4	24.4	9.5	6.1	1.5
**Carbofuran**	<iLOD	<iLOD	<iLOD	<iLOD	<iLOQ	<iLOD
**Chlorpyrifos**	1.1	2.3	4.3	3.2	1.9	2.7
**Diuron**	<iLOD	<iLOD	<iLOQ	<iLOD	<iLOD	<iLOD
**Fenpropimorph**	<iLOD	<iLOD	<iLOD	11.5	<iLOQ	<iLOD
**Iprovalicarb**	2.5	8.2	<iLOD	13.7	29.7	<iLOD
**Kresoxim-methyl**	<iLOD	<iLOD	<iLOD	<iLOD	<iLOD	<iLOQ
**Mecoprop**	<iLOD	<iLOD	<iLOD	1.9	0.3	0.1
**Metazachlor**	0.1	<iLOD	1.0	0.9	0.3	0.2
**Pendimethalin**	0.1	0.6	<iLOQ	0.5	0.3	1.0
**Phosalone**	<iLOD	0.8	<iLOD	<iLOD	<iLOD	<iLOQ
**Propiconazole**	0.3	0.4	<iLOD	1.4	1.0	0.4
**Prosulfocarb**	<iLOD	<iLOD	<iLOD	1.0	2.0	<iLOD
**S-Metolachlor**	<iLOD	<iLOD	<iLOD	5.1	<iLOD	<iLOD
**Tebuconazole**	<0.1	<iLOD	<iLOD	0.8	0.3	0.0
**Terbuthylazine**	<iLOD	<iLOD	0.8	<iLOD	<iLOD	<iLOD

Table S8. Origin of air samples collected at mountain sites

FT = free troposphere, PBL = planetary boundary layer

**Sites**	**Sampling period: dates**	**Air collected**
CPK	1: 28-30/04/2020	Mixed PBL and FT
	2: 12-14/05/2020	Mixed PBL and FT
HAC	2: 12-14/05/2020	**FT**
	3: 26-28/05/2020	Mixed PBL and FT
JFJ	1: 28-30/04/2020	Mixed PBL and FT
	2: 12-14/05/2020	Mixed PBL and FT
	3: 26-28/05/2020	Mixed PBL and FT
PDM	3: 26-28/05/2020	Mixed PBL and FT
SBO	2: 12-14/05/2020	**FT**
	3: 26-28/05/2020	**FT**
UFS	1: 28-30/04/2020	Mixed PBL and FT
	2: 12-14/05/2020	Mixed PBL and FT
	3: 26-28/05/2020	**FT**

Table S9. Quantification frequency (QF, in %) per site of individual pesticides present in the particulate phase

Analyte	QF	Analyte	QF	Analyte	QF	Analyte	QF
Tebuconazole	82.8	Prochloraz	34.5	Simazine	13.8	Quizalofop ethyl	3.45
Spiroxamine	79.3	Acetamiprid	27.6	Triallate	13.8	Alachlor	0
Cyprodinil	65.5	Aclonifen	27.6	Dimethomorph	10.3	Azinphos methyl	0
Fenpropidin	65.5	Metamitron	27.6	Iprovalicarb	10.3	Chloridazon	0
Fenpropimorph	65.5	Carbendazim	24.1	Oxadiazon	10.3	Chlorsulfuron	0
Metazachlor	65.5	Diuron	24.1	Tetraconazole	10.3	Cypermethrin	0
Terbuthylazine	65.5	Mecoprop	24.1	Chlorpyrifos	6.90	Diclofop-methyl	0
2,4-D	62.1	Chlorotoluron	20.7	Clomazone	6.90	Disulfoton	0
S-Metolachlor	55.2	Dimethachlor	20.7	Difenoconazole	6.90	Esbiothrin	0
Thiacloprid	55.2	Dimethoate	20.7	Lenacil	6.90	Fipronil	0
Atrazine	51.7	Epoxiconazole	20.7	Permethrin	6.90	Fluazinam	0
Boscalid	48.3	Acetochlor	17.2	Phosalone	6.90	Flusilazole	0
Pendimethalin	48.3	Dimethenamid-P	17.2	Pyrimethanil	6.90	Fonofos	0
Propiconazole	48.3	Fenoxycarb	17.2	2,4-MCPA	3.45	Linuron	0
Prosulfocarb	44.8	Fluroxypyr	17.2	Carbofuran	3.45	Malathion	0
Carbaryl	41.4	Kresoxim-methyl	17.2	Chlorpropham	3.45	Parathion methyl	0
Metalaxyl	37.9	Diazinon	13.8	Fenitrothion	3.45	Sulcotrione	0
Isoproturon	34.5	Diflufenican	13.8	Flumioxazine	3.45	Temephos	0
Pirimicarb	34.5	Metribuzin	13.8	Flurochloridone	3.45	Terbufos	0

Table S10. Summary of pesticides previously quantified in the atmosphere of polar sites.

Pesticides	CAS number	Type of sampler used	Quantified in:	Ref.:
2,6-Dichlorobenzonitrile	1194-65-6	Active sampling	Gaseous phase	*(24)*
Alachlor	15972-60-8	Active sampling	Total	*(22)*
Atrazine	1912-24-9	Active sampling	Total	*(22)*
Chlorobenzilate	510-15-6	Active sampling	Total	*(22)*
Chloroneb	2675-77-6	Active sampling	Total	*(22)*
		Active sampling	Gaseous phase	*(24)*
Chlorothalonil	1897-45-6	Active sampling	Total	*(23)*
Chlorpyrifos	2921-88-2	Active sampling	Total	*(23)*
Dacthal	1861-32-1	Active sampling	Total	*(23)*
Dichlofluanid	1085-98-9	Active sampling	Gaseous and particulate phases	*(24)*
Dicofol	115-32-2	Active sampling	Gaseous phase	*(23)*
Endosulfan	115-29-7	Active sampling	Total	*(23)*
MCPA	94-74-6	Passive sampling		*(23)*
Methoxychlor	72-43-5	Active sampling	Total	*(23)*
Metribuzin	21087-64-9	Active sampling	Total	*(23)*
Nitrapyrin	1929-82-4	Active sampling	Gaseous phase	*(24)*
Pendimethalin	40487-42-1	Active sampling	Total	*(23)*
Pentachloro-nitrobenzene	82-68-8	Active sampling	Total	*(23)*
Permethrin	52645-53-1	Active sampling	Total	*(22)*
Phosalone	2310-17-0	Active sampling	Total	*(23)*
Quizalofop-ethyl	76578-14-8	Active sampling	Total	*(23)*
Simazine	122-34-9	Active sampling	Total	*(22)*
Tefluthrin	79538-32-2	Active sampling	Total	*(23)*
Triallate	2303-17-5	Passive sampling		*(23)*
		Active sampling	Gaseous and particulate phases	*(24)*
Trifluralin	1582-09-8	Active sampling	Total	*(23)*

Total = the gaseous and particulate phases were extracted together, therefore no distinction can be made

Table S11. Physico-chemical properties and half-lives in different environmental matrices of the individual herbicides (A), insecticides (B) and fungicides (C) analysed in this study

(A)

Pesticide	Half-life in air (hours, 25°C)[36]	Half-life in soil (days)[57]	Half-life in water at pH = 7 (days)[57]	log K_{OA} (25°C) (est.)[36]	Saturation vapor pressure (25°C, Pa, est.)[36]	Saturation vapor pressure (Pa, exp.)[36]	Characteristic travel distance (CTD, km)[a]
2,4-D	19.35	28.8	7.7	8.65	3.7E-03	1.1E-02 [b]	286
2,4-MCPA	10.166	25	13.5	10.51	6.0E-02	7.9E-04 [c]	49
Acetochlor	2.589	12.1	40.5	9.41	3.7E-03	*N.A.*	101
Aclonifen	10.1	80.4	197	10.84	3.3E-04	1.6E-05 [c]	486
Alachlor	2.847	14	2	9.99	2.7E-03	*N.A.*	53
Atrazine	4.693	29	80	9.63	3.8E-03	3.9E-05 [c]	199
Chloridazon	3.192	18.6	51.5	9.01	3.9E-05	6.0E-05 [b]	128
Chlorotoluron	3.288	12.5	44.4	10.64	1.8E-03	4.8E-06 [b]	110
Chlorpropham	3.25	13.1	60	8.14	4.5E-01	2.4E-02 [b]	149
Chlorsulfuron	50.3945	36.2	21	15.85	1.6E-08	3.0E-09 [c]	52
Clomazone	5.839	27.3	54	8.27	9.4E-01	1.9E+00 [c]	134
Diclofop-methyl	6.763	19	0.06	8.71	9.2E-04	4.7E-04 [c]	150
Diflufenican	40.158	64.6	175	9.78	5.4E-07	4.2E-06 [c]	682
Dimethachlor	3.174	3.2	15.7	9.34	5.8E-02	1.5E-03 [c]	39
Dimethenamid-P	2.452	15.8	24	7.62	8.1E-03	3.7E-02 [c]	60
Diuron	11.801	229	8.8	10.37	6.2E-04	9.2E-06 [c]	22
Flumioxazin	2.26	17.6	4	7.14	2.4E-10	3.2E-04 [d]	10
Flurochloridone	17.183	40.6	6.55	9.16	2.2E-03	4.4E-04 [c]	269
Fluroxypyr	4.449	3	10.5	12.54	2.6E-05	3.8E-08 [b]	26
Isoproturon	2.303	23	40	11.21	1.1E-03	3.3E-06 [c]	100
Lenacil	2.756	39.8	91	11.59	4.0E-08	2.0E-07 [c]	226
Linuron	12.409	48	13	9.79	1.6E-03	1.9E-04 [c]	123
Mecoprop	7.378	*N.A.*	37	10.64	6.1E-02	*N.A.*	92
Metamitron	6.595	11.1	10.5	11.24	9.9E-05	8.6E-07 [b]	26
Metazachlor	2.174	6.8	216	9.77	1.5E-04	*N.A.*	534
Metribuzin	7.028	19	41	10.02	6.9E-04	5.8E-05 [b]	102
Oxadiazon	5.272	165	17.9	10.33	4.5E-05	1.5E-05 [d]	120
Pendimethalin	4.223	100.6	4	9.64	2.7E-02	4.0E-03 [c]	10
Prosulfocarb	3.934	9.8	0.94	10.93	6.4E-03	6.9E-05 [c]	115
Quizalofop ethyl	4.513	60	39	10.64	5.9E-06	8.7E-07 [b]	100
Simazine	7.201	90	46	9.59	1.2E-03	3.0E-06 [c]	114
S-Metolachlor	2.296	0.9654167	9	9.33	6.7E-01	*N.A.*	42
Sulcotrione	17.085	0.15	9.5	10.71	1.2E-06	5.0E-06 [c]	24
Terbuthylazine	13.549	21.8	6	9.03	2.2E-03	1.5E-04 [c]	222
Triallate	3.851	46	104	7.91	2.4E-02	1.6E-02 [c]	176

[a] using the OECD screening tool for LRT[65] input data as listed with estimated values for log K_{OW} and log K_{AW}[36]; using a global annual mean OH concentration of 0.75×10^{6} cm^{-3} [36]; using experimental values for half-lives in soil. [b] T = 293 K; [c] T = 298 K; [d] T= 295 K; *N.A.* = not applicable

(B)

Pesticide	Half-life in air (hours, 25°C)[36]	Half-life in soil (days)[57]	Half-life in water at pH = 7 (days)[57]	log K_{OA} (25°C) (est.)[36]	Saturation vapor pressure (25°C, Pa, est.)[36]	Saturation vapor pressure (Pa, exp.)[36]	Characteristic travel distance (CTD, km)[a]
Acetamiprid	1.679	3	4.7	8.1	5.81E-03	*N.A.*	34
Azinphos methyl	0.842	*N.A.*	8	11.38	1.02E-05	*N.A.*	20
Carbaryl	4.937	*N.A.*	3.1	9.23	7.11E-03	1.81E-04 [b]	62
Carbofuran	4.935	14	6.1	9.22	7.38E-03	6.47E-04 [c]	54
Chlorpyrifos	1.4	27.6	5	8.88	2.73E-03	2.71E-03 [b]	29
Cypermethrin	5.99	0.9125	3	11.71	1.74E-05	4.09E-07 [b]	183
Diazinon	1.328	18.4	4.3	9.15	7.24E-03	1.20E-02 [b]	28
Dimethoate	1.625	7.2	12.6	8.78	5.50E-03	2.51E-03 [b]	31
Disulfoton	0.963	*N.A.*	15	8.07	4.66E-02	1.30E-02 [b]	37
Esbiothrine	0.578	32	*N.A.*	10.15	4.70E-03	1.60E-04 [d]	N.A.
Fenitrothion	2.143	*N.A.*	1.1	7.72	1.02E-02	7.20E-03 [e]	44
Fenoxycarb	1.966	5.94	4.13	12.06	5.90E-04	8.67E-07 [b]	116
Fipronil	1.336	65	54	11.46	1.51E-07	3.71E-07 [b]	134
Fonofos	1.493	40	277.5	7.49	7.34E-02	4.51E-02 [b]	420
Malathion	1.658	1	0.4	9.06	3.28E-02	4.51E-04 [b]	29
Parathion methyl	2.174	10	15	8.25	1.55E-02	4.67E-04 [b]	44
Permethrin	5.609	42	23	10.62	1.10E-04	2.91E-06 [b]	151
Phosalone	0.408	*N.A.*	2.8	9.17	1.55E-05	*N.A.*	9
Pirimicarb	0.847	9	29.6	9.16	1.50E-02	9.71E-04 [b]	74
Temephos	0.946	*N.A.*	17.2	13.06	1.05E-05	*N.A.*	980
Terbufos	0.528	12	16	7.49	4.66E-02	4.27E-02 [b]	37
Thiacloprid	1.438	8.1	1000	10.33	1.51E-04	*N.A.*	2398

[a] using the OECD screening tool for LRT[65] input data as listed with estimated values for log K_{OW} and log K_{AW}[36]; global annual mean =0.75e6 OH cm^{-3} [36]; experimental values for half-lives in soil. [b] T=298 K; [c] T=292 K; [d] T=294 K; [e] T=293 K; *N.A.* = not applicable

(C)

**Pesticide**	**Half-life in air (hours, 25°C)[36]**	**Half-life in soil (days)[57]**	**Half-life in water at pH = 7 (days)[57]**	**log K_{OA} (25°C) (est.)[36]**	**Saturation vapor pressure (25°C, Pa, est.)[36]**	**Saturation vapor pressure (Pa, exp.)[36]**	**Characteristic travel distance (CTD, km)[a]**
Boscalid	14.194	254	5	12.72	9.19E-09	*N.A.*	73
Carbendazim	0.64	22	7.9	10.58	7.25E-07	1.00E-07 [b]	20
Cyprodinil	0.641	45	12.5	9.46	5.31E-03	4.91E-05 [c]	31
Difenoconazole	5.95	91.8	3	13.71	2.42E-06	3.33E-08 [c]	142
Dimethomorph	1.203	44	10	10.77	2.57E-07	9.84E-07 [c]	25
Epoxiconazole	14.622	97.7	1000	11.23	3.75E-05	*N.A.*	2394
Fenpropidin	1.137	49.2	1.8	11.87	2.92E-01	1.71E-02 [c]	160
Fenpropimorph	0.93	25.5	2.65	8.93	9.93E-03	3.51E-03 [b]	19
Fluazinam	3919.2	25.9	4.5	10.68	7.34E-06	*N.A.*	205
Flusilazole	20.83	94	1	8.38	2.55E-03	3.91E-05 [c]	384
Iprovalicarb	2.397	15.5	54	11.07	2.76E-06	*N.A.*	134
Kresoxim-methyl	3.356	1	0.85	10.24	1.67E-04	2.29E-06 [b]	51
Metalaxyl	4.774	14.1	56	8.63	7.71E-03	7.49E-04 [c]	139
Prochloraz	1.645	68.8	2	10.27	2.13E-06	1.51E-04 [c]	37
Propiconazole	5.533	35.2	6	10.87	9.95E-04	5.60E-05 [c]	80.3
Pyrimethanil	0.641	31.4	6.7	8.68	1.37E-02	2.20E-03 [c]	17
Spiroxamine	0.999	52.4	0.8	10.87	3.22E-02	1.71E-02 [c]	33
Tebuconazole	11.184	47.1	1.775	11.93	6.14E-06	1.71E-06 [b]	41
Tetraconazole	11.677	460	2	10.32	1.10E-02	1.80E-04 [b]	108

[a] using the OECD screening tool for LRT[65] input data as listed with estimated values for log K_{OW} and log K_{AW}[36]; global annual mean =0.75e6 OH cm^{-3} [36]; experimental values for half-lives in soil. [b] T=293 K;
[c] T=298 K; *N.A.* = not applicable

Table S12. Average particulate mass fraction (θ, %) of pesticides measured at both rural and polar sites where both phases were sampled

	Rural sites		Polar sites	
	BKO	KOS	ADA	ZPO
2,4-D	*N.A.*	100	0	*N.A.*
Carbaryl	100	73.6	0	*N.A.*
Carbendazim	0	100	0	0
Chlorpyrifos	0	34.7	0	0
Cyprodinil	*N.A.*	100	*N.A.*	0
Fenpropidin	*N.A.*	100	*N.A.*	0
Fenpropimorph	100	100	100	0
Pendimethalin	0	28.4	0	0
Prochloraz	*N.A.*	100	*N.A.*	0
Prosulfocarb	0	62.6	*N.A.*	0
Spiroxamine	*N.A.*	100	*N.A.*	0
Tebuconazole	100	100	0	*N.A.*
Terbuthylazine	*N.A.*	98.8	*N.A.*	0
Thiacloprid	*N.A.*	100	*N.A.*	0
Triallate	0	0	100	*N.A.*

N.A.: not applicable

Table S13. Selected HPLC-MS/MS experimental parameters, instrumental limits of detection (iLODs) and instrumental limits of quantification (iLOQs)

**Analyte**	**Precursor ion** (m/z)	**Product ion 1** (m/z)	**Product ion 2** (m/z)	**R_t** (min)	**iLOD** (ng/mL)	**iLOQ** (ng/mL)
2,4-D	218.8	160.9	124.9	2.4	0.02	0.06
Acetamiprid	222.9	142.0	*N.A.*	3.2	0.08	0.29
Acetochlor	270.1	224.2	148.1	4.9	0.10	0.25
Alachlor	270.1	238.1	162.1	5.0	0.10	0.25
Atrazine	216.1	174.2	68.0	3.8	0.01	0.03
Azinphos methyl	318.0	130.0	160.0	4.1	0.03	0.10
Boscalid	343.2	307.1	140.1	5.9	0.12	0.40
Carbaryl	201.9	145.0	127.1	3.0	0.10	0.30
Carbendazim	192.0	160.0	131.9	1.2	0.03	0.05
Carbofuran	222.1	165.1	123.0	5.0	0.04	0.13
Chloridazon	222.0	104.0	77.0	2.3	0.06	0.12
Chlorotoluron	213.1	72.2	46.2	3.7	0.10	0.30
Chlorpyrifos	349.9	197.9	96.9	6.7	0.01	0.03
Chlorsulfuron	357.9	141.0	167.0	3.0	0.01	0.03
Cyprodinil	226.0	93.1	108.1	5.8	0.11	0.36
Diazinon	305.0	169.0	153.1	5.4	0.01	0.03
Dimethachlor	256.1	224.0	148.1	4.0	0.03	0.10
Dimethoate	230.0	198.9	124.9	2.2	0.03	0.10
Disulfoton	274.9	88.9	61.0	5.8	0.05	0.10
Diuron	232.9	71.8	46.1	4.0	0.10	0.25
Fenitrothion	277.9	125.1	109.0	4.7	0.30	1.00
Fenpropidin	274.1	147.1	86.1	5.5	0.10	0.34
Fenpropimorph	304.1	147.1	117.1	2.4	0.01	0.03
Fluroxypyr	255.0	180.9	208.9	2.7	1.00	3.00
Fonofos	246.9	109.0	137.0	5.4	0.10	0.25
Iprovalicarb	321.0	119.0	203.0	6.1	0.10	0.32
Isoproturon	207.2	72.1	46.1	3.9	0.01	0.03
Kresoxim methyl	314.0	116.1	131.1	6.3	0.22	0.74
Malathion	331.0	127.0	99.0	4.5	0.10	0.30
Mecoprop	212.9	141.0	71.1	2.5	0.02	0.07
Metalaxyl	280.0	220.1	192.0	5.5	0.12	0.41
Metamitron	203.0	175.1	104.0	2.2	0.25	0.50
Metazachlor	278.2	134.2	210.2	3.7	0.03	0.05
Metribuzin	215.1	187.1	84.0	3.3	0.03	0.10
Oxadiazon	345.0	303.0	219.9	6.7	0.06	0.22
Parathion methyl	264.0	125.0	232.0	4.0	0.50	0.10
Pendimethalin	282.2	212.0	194.2	6.4	0.01	0.04
Phosalone	367.9	111.0	181.9	6.4	0.07	0.25
Pirimicarb	239.0	72.0	182.1	2.1	0.01	0.03
Prochloraz	376.0	308.0	70.0	4.9	0.01	0.03
Propiconazole	342.0	159.0	69.0	5.0	0.03	0.10
Prosulfocarb	252.0	91.1	128.1	6.6	0.06	0.21
Quizalofop ethyl	273.0	299.0	271.0	6.6	0.03	0.10
Simazine	202.0	132.0	124.0	3.3	0.01	0.03
S-Metolachlor	284.1	252.2	176.1	4.9	0.01	0.03
Spiroxamine	298.1	144.2	100.0	5.6	0.09	0.29
Tebuconazole	308.1	70.0	125.0	4.9	0.01	0.03
Temephos	466.9	419.0	124.9	6.3	0.03	0.05
Terbufos	288.9	103.0	233.0	6.4	0.05	0.10
Terbuthylazine	230.0	174.0	96.0	4.5	0.01	0.03
Thiacloprid	252.9	126.0	90.0	4.4	0.04	0.14

R_t = retention time

Table S14. Selected GC-MS/MS and UPLC-MS/MS experimental parameters, instrumental limits of detection (iLODs) and instrumental limits of quantification (iLOQs)

**Analyte**	**Precursor ion** (m/z)	**Product ion 1** (m/z)	**Product ion 2** (m/z)	**R_t** (min)	**iLOD** (ng/mL)	**iLOQ** (ng/mL)
2,4-MCPA	199.0	201.0	141.0	2.1	*N.A.*	142.80
Aclonifen	264.0	194.0	*N.A.*	17.9	3.26	9.77
Chlorpropham	213.1	127.0	*N.A.*	10.0	1.46	4.37
Clomazone	124.9	88.9	*N.A.*	10.9	1.36	4.09
Cypermethrin	163.0	127.0	*N.A.*	25.7	1.19	3.57
Diclofop-methyl	253.0	162.2	*N.A.*	19.6	4.57	13.70
Difenoconazole	323.1	265.0	*N.A.*	28.2	1.46	4.37
Diflufenican	394.1	266.1	*N.A.*	19.6	0.45	1.34
Dimethenamid-P	230.0	154.4	*N.A.*	12.2	1.36	4.09
Dimethomorph	301.1	165.1	*N.A.*	29.3	2.47	7.41
Epoxiconazole	191.9	138.0	*N.A.*	19.9	0.59	1.78
Esbiothrin	123.0	81.1	*N.A.*	14.9	3.52	10.55
Fenoxycarb	186.1	109.1	*N.A.*	20.9	6.75	20.26
Fipronil	367.0	213.0	*N.A.*	14.6	0.74	2.23
Fluazinam	462.9	415.9	398.0	2.3	*N.A.*	26.28
Flumioxazin	354.1	176.1	*N.A.*	27.3	2.72	8.17
Flurochloridone	311.0	174.1	*N.A.*	14.0	0.45	1.34
Flusilazole	233.1	183.1	*N.A.*	16.7	0.68	2.04
Lenacil	152.9	136.2	*N.A.*	18.9	1.46	4.37
Linuron	248.0	61.0	*N.A.*	13.3	3.86	11.59
Permethrin	183.0	165.0	*N.A.*	24.1	0.59	1.78
Pyrimethanil	198.1	118.1	*N.A.*	11.4	1.19	3.57
Sulcotrione	329.0	293.0	139.0	2.17	N.A.	67.5
Tetraconazole	336.0	204.0	*N.A.*	13.8	0.59	1.78
Triallate	267.9	184.0	*N.A.*	11.7	*0.89*	2.68

R_t = retention time; *N.A.* = not applicable

Table S15. Limits of quantifications determined from field blanks (LOQb) (in pg m^{-3})

The average sampled volumes were used to calculate the concentrations (1732 m^3 for HVAS and 114 m^3 for LVAS). The compounds never found in field blanks are not included.

Analyte	*LOQb value*			
	Particulate phase (HVAS)	***Particulate phase (LVAS)***	***Gaseous phase (HVAS)***	***Gaseous phase (LVAS)***
2,4-D	0.55	0.44	0.42	6.41
2,4-MCPA	0.09	0.53	<LOD	<LOD
Acetochlor	0.96	<LOD	0.48	7.38
Aclonifen	0.01	0.4	0.03	0.44
Carbendazim	3	27.67	5.89	89.7
Carbofuran	0.04	<LOD	0	0.70
Chlorpyrifos	1.28	2	4.73	71.9
Cypermethrin	0.09	1.49	<LOD	<LOD
Cyprodinil	0.42	2.6	<LOD	<LOD
Diclofop-methyl	<LOD	0	0.01	0.09
Difenoconazole	<LOD	0	0.02	0.26
Dimethomorph	0.01	0	<LOD	<LOD
Diuron	<LOD	<LOD	0.18	2.81
Fenoxycarb	0.01	0.18	0.01	0.09
Fenpropidin	0.31	1.49	<LOD	<LOD
Fenpropimorph	0.27	<LOD	2.89	43.9
Fipronil	<LOD	<LOD	0.01	0.09
Fluazinam	0.24	1.58	<LOD	<LOD
Flumioxazin	0.01	0.09	0.05	0.79
Iprovalicarb	4.58	45.7	18.0	273
Kresoxim-methyl	<LOD	<LOD	0.27	4.13
Lenacil	<LOD	<LOD	0.12	1.84
Mecoprop	0.23	<LOD	0.76	11.6
Metazachlor	0.03	<LOD	0.38	5.71
Oxadiazon	0.06	<LOD	<LOD	<LOD
Pendimethalin	0.91	2.20	1.58	24.1
Phosalone	0.09	<LOD	0.91	13.9
Pirimicarb	0.02	<LOD	<LOD	<LOD
Propiconazole	0.39	0.44	0.58	8.78
Prosulfocarb	1.54	3.43	0.91	13.9
Quizalofop ethyl	0.02	0.44	<LOD	<LOD
S-Metolachlor	2.42	1.41	1.22	18.5
Spiroxamine	0.16	1.23	<LOD	<LOD
Tebuconazole	0.06	0.88	0.21	3.16
Terbuthylazine	0.64	1.76	0.13	1.93

HVAS = High-volume air sampler; LVAS = Low-volume air sampler

Table S16. Recoveries (in %) and standard deviations (SD) of individual herbicides (A), insecticides (B) and fungicides (C) analysed using HPLC-MS/MS determined from spike recovery tests of air sampling media (QFFs and PUF-XAD2-PUF sandwiches, n=5 each)

(A)

	QFF		PUF-XAD2-PUF	
**Compounds**	**Average recovery (%)**	**SD**	**Average recovery (%)**	**SD**
2,4-D	102	3.8	101	5.8
Acetochlor	76.3	8.2	91	6.9
Alachlor	73.9	4.3	79.7	5.8
Atrazine	72.8	6.9	75.6	7.7
Chloridazon	61.6	4.1	61.1	1.6
Chlorotoluron	103	17	97.5	3.6
Chlorsulfuron	96.6	10	46.8	5.3
Dimethachlor	83.6	6.5	89.3	4.2
Diuron	65.4	5.4	100	6.8
Fluroxypyr	65.4	6.6	80.5	8.1
Isoproturon	111	19	96	4.8
Mecoprop	107	4.6	105	7.6
Metamitron	105	14	96.5	6.3
Metazachlor	113	19	104	4.2
Metribuzin	72.4	7	70.1	6
Oxadiazon	65	37	35.5	2.2
Pendimethalin	102	15	102	7.1
Prosulfocarb	106	4.4	95.8	8.6
Quizalofop ethyl	100	1.7	100	9.3
Simazine	104	19	93.4	2.8
S-metolachlor	74.4	2.5	99.7	6.2
Terbuthylazine	103	16	86	2.4

(B)

Compounds	QFF<br>Average recovery (%)	SD	PUF-XAD2-PUF<br>Average recovery (%)	SD
Acetamiprid	101	5.4	92.6	5.2
Azinphos methyl	77.3	8.8	63.8	10
Carbaryl	67.6	13	83.6	4
Carbofuran	102	7.6	79.8	4.4
Chlorpyrifos	77.8	7.3	68.8	3.8
Diazinon	61.4	16	34.1	13
Dimethoate	78.4	5.6	79	2
Disulfoton	66.4	8.8	71	7.2
Fenitrothion	70	5.2	104	15
Fonofos	80.6	12	56.7	4.3
Malathion	98.8	27	98.6	11
Parathion methyl	72.9	13	66.8	4.1
Phosalone	109	3.9	116	5.9
Pirimicarb	79.5	15	68.6	4.6
Temephos	99.9	13	57.2	34
Terbufos	74.4	4.7	47.3	9.2
Thiacloprid	103	4.5	100	2.4

(C)

Compounds	QFF Average recovery (%)	QFF SD	PUF-XAD2-PUF Average recovery (%)	PUF-XAD2-PUF SD
Boscalid	78.8	44	95.6	20
Carbendazim	102	14	90.2	3.8
Cyprodinil	99.6	6.5	104	2.9
Fenpropidin	102	4	97.8	11
Fenpropimorph	152	20	62.2	5.5
Iprovalicarb	52	27	41.4	6.6
Kresoxim methyl	72.8	3.4	86.4	16
Metalaxyl	103	6.4	112	7.7
Prochloraz	106	17	94.4	5.1
Propiconazole	109	20	103	4.3
Spiroxamine	102	3.9	98.4	11
Tebuconazole	67.9	4.9	80.8	5.9

Table S17. Results of the multiple linear regression statistical analysis applied to the number of pesticides found at each site

Variable	P-value	Coefficient
Latitude	0.0002	-0.55
Site category	0.0003	-4.887
Agricultural area (%)	0.0242	0.242

Table S18. Summary of pesticides previously quantified in the atmosphere at high mountain sites.

Pesticide	CAS number	Reference:	Location (altitude, meter a.s.l.)	Type of sampler used	Quantified in:
Chlorothalonil	1897-45-6	*(76)*	Sierra Nevada, California, USA (1920 m)	Active sampling	Total
		(77)	British Columbia and Alberta, Canada (>2000 m)	Passive sampling	Gaseous phase
Chlorpyrifos	2921-88-2	*(78)*	Sierra Nevada, California, USA (1920 m)	Active sampling	Total
		(76)	Sierra Nevada, California, USA (1920 m)	Active sampling	Total
		(77)	British Columbia and Alberta, Canada (>2000 m)	Passive sampling	Gaseous phase
		(79)	Southeast Brazil (2200 - 2470 m)	Passive sampling	
Cypermethrin	52315-07-8	*(79)*	Southeast Brazil (2200 - 2470 m)	Passive sampling	
Dacthal	1861-32-1	*(77)*	British Columbia and Alberta, Canada (>2000 m)	Passive sampling	Gaseous phase
Diazinon	333-45-5	*(76)*	Sierra Nevada, California, USA (1920 m)	Active sampling	Total
Ethephon	16672-87-0	*(20)*	Col Margherita Atmospheric Observatory, Italy (2543 m)	Active sampling	Particulate phase
Fosetyl-aluminium	39148-24-8	*(20)*	Col Margherita Atmospheric Observatory, Italy (2543 m)	Active sampling	Particulate phase
Glufosinate	51276-47-2	*(20)*	Col Margherita Atmospheric Observatory, Italy (2543 m)	Active sampling	Particulate phase
Glyphosate	1071-83-6	*(20)*	Col Margherita Atmospheric Observatory, Italy (2543 m)	Active sampling	Particulate phase
Maleic hydrazide	123-33-1	*(20)*	Col Margherita Atmospheric Observatory, Italy (2543 m)	Active sampling	Particulate phase
Methoxychlor	72-43-5	*(79)*	Southeast Brazil (2200 - 2470 m)	Passive sampling	
Pentachloronitrobenzene	82-68-8	*(77)*	British Columbia and Alberta, Canada (>2000 m)	Passive sampling	Gaseous phase
Permethrin	52645-53-1	*(79)*	Southeast Brazil (2200 - 2470 m)	Passive sampling	
Trifluralin	1582-09-8	*(76)*	Sierra Nevada, California, USA (1920 m)	Active sampling	Total

Total = the gaseous and particulate phases were extracted together, therefore no distinction can be made

References

(1) Alexandratos, N.; Bruinsma, J. World Agriculture towards 2030/2050: The 2012 Revision. ESA working paper 12−03; Rome. www.fao.org/economic/esa (accessed April 20, 2022).
(2) FAOSTAT. FAOSTAT: Pesticides use. In FAO.org. www.fao.org/faostat/en/#data/RP (accessed March 21, 2022).
(3) Sharma, A.; Kumar, V.; Shahzad, B.; Tanveer, M.; Sidhu, G. P. S.; Handa, N.; Kohli, S. K.; Yadav, P.; Bali, A. S.; Parihar, R. D.; Dar, O. I.; Singh, K.; Jasrotia, S.; Bakshi, P.; Ramakrishnan, M.; Kumar, S.; Bhardwaj, R.; Thukral, A. K. Worldwide pesticide usage and its impacts on ecosystem. *SN Appl. Sci.* **2019**, *1*, 1446
(4) Franklin, J.; Atkinson, R.; Howard, P. H.; Orlando, J. J.; Seigneur, C.; Wallington, T. J.; Zetsch, C. Quantitative Determination of Persistence in Air. In *Criteria for Persistence and Long-Range Transport of Chemicals in the Environment*; Klecka, G.; Boethling, B.; Franklin, J.; Grady, L.; Graham, D.; Howard, P. H.; Kannan, K.; Larson, R. J.; Mackay, D.; Muir, D.; van de Meent, D., eds.; SETAC press: Pensacola, 2000; pp 7−62.
(5) British Crop Protection Council. *The Pesticide Manual: A World Compendium*, 16th ed.; MacBean, C., Ed.; BCPC Publications: Alton, UK, 2012.
(6) Carvalho, F. P. Pesticides, Environment, and Food Safety. *Food Energy Secur.* 2017, *6*, 48−60.
(7) Rico, A.; van den Brink, P. J. Evaluating aquatic invertebrate vulnerability to insecticides based on intrinsic sensitivity, biological traits, and toxic mode of action. *Environ. Toxicol. Chem.* **2015**, *34*, 1907−1917.
(8) Sanchez-Bayo, F.; Wyckhuys, K. A. G. Worldwide decline of the entomofauna: A review of its drivers. *Biol. Conserv.* **2019**, *232*, 8−27.
(9) Vorkamp, K.; Riget, F. F. A review of new and current-use contaminants in the Arctic environment: evidence of long-range transport and indications of bioaccumulation. *Chemosphere* **2014**, *111*, 379−395.
(10) AMAP. AMAP Assessment 2016: Chemicals of Emerging Arctic Concern; Oslo, Norway. www.amap.no (accessed March 19, 2023).
(11) Mostafalou, S.; Abdollahi, M. Pesticides: An update of human exposure and toxicity. *Arch. Toxicol.* **2017**, *91* (2), 549−599.
(12) van den Berg, F.; Kubiak, R.; Benjey, W. G.; Majewski, M. S.; Yates, S. R.; Reeves, G. L.; Smelt, J. H.; van der Linden, A. M. A. Emission of pesticides into the air. *Water Air Soil Pollut.* **1999**, *115*, 195−218.
(13) Glotfelty, D. E.; Leech, M. M.; Jersey, J.; Taylor, A. W. Volatilization and wind erosion of soil surface applied atrazine,
simazine, alachlor and toxaphene. *J. Agric. Food Chem.* **1989**, *37*, 546−551.
(14) Davie-Martin, C. L.; Hageman, K. J.; Chin, Y. P.; Rouge, V.; Fujita, Y. Influence of temperature, relative humidity, and soil properties on the soil-air partitioning of semivolatile pesticides: laboratory measurements and predictive models. *Environ. Sci. Technol.* **2015**, *49* (17), 10431−10439.
(15) Degrendele, C.; Okonski, K.; Melymuk, L.; Landlová, L.; Kukučka, P.; Audy, O.; Kohoutek, J.; Čupr, P.; Klánová, J. Pesticides in the atmosphere: a comparison of gas-particle partitioning and particle size distribution of legacy and current-use pesticides. *Atmos. Chem. Phys.* **2016**, *16* (3), 1531−1544.
(16) Desert, M.; Ravier, S.; Gille, G.; Quinapallo, A.; Armengaud, A.; Pochet, G.; Savelli, J. L.; Wortham, H.; Quivet, E. Spatial and temporal distribution of current-use pesticides in ambient air of Provence-Alpes-Côte-d'Azur Region and Corsica, France. *Atmos. Environ.* **2018**, *192*, 241−256.
(17) Lopez, A.; Yusà, V.; Muñoz, A.; Vera, T.; Borràs, E.; Rodenas, M.; Coscollà C. Risk assessment of airborne pesticides in a Mediterranean region of Spain. *Sci. Total Environ.* **2017**, *574*, 724−734.
(18) Klöppel, H.; Kördel, W. Pesticides volatilisation and exposure of terrestrial ecosystems. *Chemosphere* **1997**, *35* (6), 1271−1289.
(19) Gkotsis, G.; Nika, M. C.; Nikolopoulou, V.; Alygizakis, N.; Bizani, E.; Aalizadeh, R.; Badry, A.; Chadwick, E.; Cincinelli, A.;Clasen, D.; Danielsson, S.; Dekker, R.; Duke, G.; Drost, W.; Glowacka, N.; Gockener, B.; Jansman, H. A. H.; Juergens, M.; Knopf, B.; Koschorreck, J.; Krone, O.; Martellini, T.;

Movalli, P.; Persson, S.; Potter, E. D.; Rohner, S.; Roos, A.; O' Rourke, E.; Siebert, U.; Treu, G.; van den Brink, N. W.; Walker, L. A.; Williams, R.; Slobodnik, J.; Thomaidis, N.
S. Assessment of Contaminants of Emerging Concern in European apex predators and their prey by LC-QToF MS wide-scope target analysis. *Environ. Int.* **2022**, *170*, 107623.
(20) Feltracco, M.; Barbaro, E.; Maule, F.; Bortolini, M.; Gabrieli, J.; De Blasi, F.; Cairns, W. R.; Dallo, F.; Zangrando, R.; Barbante, C.; Gambaro, A. Airborne Polar pesticides in rural and mountain sites of north-eastern Italy: an emerging air quality issue. *Environ. Pollut.* **2022**, *308*, 119657.
(21) Mai, C.; Theobald, N.; Lammel, G.; Hühnerfuss, H. Spatial, Seasonal and vertical distributions of currently-used pesticides in the marine boundary layer of the North Sea. *Atmos. Environ.* **2013**, *75*, 92−102.
(22) Gao, Y.; Zheng, H.; Xia, Y.; Chen, M.; Meng, X. Z.; Cai, M. Spatial Distributions and seasonal changes of current-use pesticides from the North Pacific to the Arctic Oceans. *J. Geophys. Res.: Atmos.* **2019**, *124* (16), 9716−9729.
(23) Balmer, J. E.; Morris, A.D.; Hung,H.; Jantunen, L.; Vorkamp, K.; Riget, F.; Evans, M.; Houde, M.; Muir, D. C. G. Levels and trends of current-use pesticides (CUPs) in the Arctic: An updated review, 2010−2018. *Emerging Contam.* **2019**, *5*, 0−88. (24) Rohler, L.; Schlabach, M.; Haglund, P.; Breivik, K.; Kallenborn, R.; Bohlin-Nizzetto, P. Non-target and suspect characterisation of organic contaminants in Arctic air - Part 2: Application of a new tool for identification and prioritisation of Chemicals of Emerging Arctic Concern in air. *Atmos. Chem. Phys.* **2020**, *20* (14), 9031−9049.
(25) *IVL, Nationell Luftövervakning 2019, Report No. C584* Swedish Environmental Research Institute: Stockholm 2021.
(26) UNEP. Stockholm Convention on Persistent Organic Pollutants (POPs) 2001 http://chm.pops.int/tabid/208/Default.aspx (accessed March 20, 2023).
(27) *European Commission, Regulation No. 107/, Regulation of the European Parliament and of the Council of 21 October 2009 concerning the placing of plant protection products on the market and repealing council directives 79/117/EEC and 91/414/EEC2009L3091*, 2009.
(28) Semeena, V. S.; Lammel, G. The significance of the grasshopper effect on the atmospheric distribution of persistent organic substances *Geophys. Res. Lett.* **2005**, *32* (7).
(29) Bidleman, T. F. Atmospheric processes. *Environ. Sci. Technol.* **1988**, *22* (4), 361−367.
(30) Borras, E.; Rodenas, M.; Vazquez, M.; Vera, T.; Muñoz, A. Particulate and gas-phase products from the atmospheric degradation of chlorpyrifos and chlorpyrifos-oxon. *Atmos. Environ.* **2015**, *123*, 112−120.
(31) Murschell, T.; Farmer, D. K. Atmospheric OH oxidation of three chlorinated aromatic herbicides. *Environ. Sci. Technol.* **2018**, *52* (8), 4583−4591.
(32) Murschell, T.; Farmer, D. K. Atmospheric OH oxidation chemistry of trifluralin and acetochlor. *Environ. Sci. Process Impacts* **2019**, *21* (4), 650−658.
(33) Socorro, J.; Durand, A.; Temime-Roussel, B.; Gligorovski, S.; Wortham, H.; Quivet, E. The persistence of pesticides in atmospheric particulate phase: an emerging air quality issue. *Sci. Rep.* **2016**, *6* (1), 33456.
(34) Socorro, J.; Lakey, P. S. J.; Han, L.; Berkemeier, T.; Lammel, G.; Zetzsch, C.; Pöschl, U.; Shiraiwa, M. Heterogeneous OH oxidation, shielding effects, and implications for the atmospheric fate of terbuthylazine and other pesticides. *Environ. Sci. Technol.* **2017**, *51* (23), 13749−13754.
(35) Muñoz, A.; Borras, E.; Vera, T.; Colmenar, I.; Rodenas, M.; Gimeno, C.; Fuentes, E.; Coscollà, C.; Calvete-Sogo, H. Atmospheric degradation of two pesticides mixed with volatile organic compounds emitted by citrus trees. Ozone and secondary organic aerosol production. *Atmos. Environ.* **2023**, *295*, 119541.
(36) US EPA. *Estimation Programs Interface Suite™ for Microsoft Windows*. United States Environmental Protection Agency: Washington, DC, USA, 2012.
(37) CORINE Land Cover. European Environment Agency (EEA). © European Union, Copernicus Land Monitoring Service. 2018.
(38) Lopez, A.; Coscollà, C.; Yusà, V. Evaluation of sampling adsorbents and validation of a LC-HRMS method for determination of 28 airborne pesticides. *Talanta* **2018**, *189*, 211−219.

(39) Pisso, I.; Sollum, E.; Grythe, H.; Kristiansen, N. I.; Cassiani, M.; Eckhardt, S.; Arnold, D.; Morton, D.; Thompson, R. L.; Groot Zwaaftink, C. D.; Evangeliou, N.; Sodemann, H.; Haimberger, L.; Henne, S.; Brunner, D.; Burkhart, J. F.; Fouilloux, A.; Brioude, J.; Philipp, A.; Seibert, P.; Stohl, A. The Lagrangian particle dispersion model FLEXPART Version 10.4. *Geosci. Model Dev.* **2019**, *12* (12), 4955−4997.
(40) Simon, A.; Belluš, M.; Čatlošova, K.; Derková, M.; Dian, M.; Imrišek, M.; Kaňák, J.; Meri, L.; Neštiak, M.; Vivoda, J. Numerical simulations of June 7, 2020 convective precipitation over Slovakia using deterministic, probabilistic, and convection-permitting approaches. *Idöjaras* **2021**, *125* (4), 571−607.
(41) Collaud Coen, M. C.; Andrews, E.; Aliaga, D.; Andrade, M.; Angelov, H.; Bukowiecki, N.; Ealo, M.; Fialho, P.; Flentje, H.; Hallar, A. G.; Hooda, R.; Kalapov, I.; Krejci, R.; Lin, N.H.; Marinoni, A.; Ming, J.; Nguyen, N. A.; Pandolfi, M.; Pont, V.; Ries, L.; Rodriguez, S.; Schauer, G.; Sellegri, K.; Sharma, S.; Sun, J.; Tunved, P.; Velasquez, P.; Ruffieux, D. Identification of topographic features influencing aerosol observations at high altitude stations. *Atmos. Chem. Phys.* **2018**, *18* (16), 12289−12313.
(42) McClure, C. D.; Jaffe, D. A.; Gao, H. Carbon Dioxide in the free troposphere and boundary layer at the Mt. Bachelor Observatory. *Aerosol Air Qual. Res.* **2016**, *16* (3), 717−728.
(43) Andrews, E.; Ogren, J. A.; Bonasoni, P.;Marinoni, A.; Cuevas, E.; Rodriguez, S.; Sun, J. Y.; Jaffe, D. A.; Fischer, E. V.; Baltensperger, U.; Weingartner, E.; Coen, M. C.; Sharma, S.; Macdonald, A. M.; Leaitch, W. R.; Lin, N. H.; Laj, P.; Arsov, T.; Kalapov, I.; Jefferson, A.; Sheridan, P. Climatology of aerosol radiative properties in the free troposphere. *Atmos. Res.* **2011**, *102*, 365−393.
(44) Herrmann, E.; Weingartner, E.; Henne, S.; Vuilleumier, L.; Bukowiecki, N.; Steinbacher, M.; Conen, F.; Collaud Coen, M. C.; Hammer, E.; Juranyi, Z.; Baltensperger, U.; Gysel, M. Analysis of long-term aerosol size distribution data from Jungfraujoch with emphasis on free tropospheric conditions, cloud influence, and air mass transport. *J. Geophys. Res.* **2015**, *120* (18), 9459−9480.
(45) Poltera, Y.; Martucci, G.; Collaud Coen, M. C.; Hervo, M.; Emmenegger, L.; Henne, S.; Brunner, D.; Haefele, A. PathfinderTURB: An automatic boundary layer algorithm. Development, validation and application to study the impact on in situ measurements at the Jungfraujoch. *Atmos. Chem. Phys.* **2017**, *17* (16), 10051−10070.
(46) Lotteraner, C.; Piringer, M. Mixing-height time series from operational ceilometer aerosol-layer heights. *Boundary Layer Meteorol.* **2016**, *161* (2), 265−287.
(47) Frank, G.; Salvamoser, J.; Steinkopff, T. Radon-222 and Beryllium-7 as natural tracer, 2019 URL: https://www.dach2019.de/
DACH2019-abstracts.pdf (accessed April 02, 2023).
(48) Thomas, W. *Personal Communication* DWD: Hohenpeißenberg, Germany; 2022.
(49) Maturilli, M. Ceilometer cloud base eight from station Ny-Alesund (2017−08 et Seq). 2022.
(50) Commission Decision 2004/248/EC of 10 March 2004 Concerning the Non-Inclusion of Atrazine in Annex I to Council Directive 91/414/EEC and the Withdrawal of Authorisations for Plant Protection Products Containing this Active Substance, 2004.
(51) Chernyak, S. M.; Rice, C. P.; McConnell, L. L. Evidence of currently-used pesticides in air, ice, fog, seawater and surfacemicrolayer in the Bering and Chukchi Seas. *Mar. Pollut. Bull.* **1996**, *32* (5), 410−419.
(52) Muir, D. C. G.; Teixeira, C.; Wania, F. Empirical and modelling evidence of regional atmospheric transport of current-use pesticides. *Environ. Toxicol. Chem.* **2004**, *23* (10), 2421−2432.
(53) Fuhrimann, S.; Klánová, J.; Přibylová, P.; Kohoutek, J.; Dalvie, M. A.; Röösli, M.; Degrendele, C. Qualitative Assessment of 27 current-use pesticides in air at 20 sampling sites across Africa. *Chemosphere* **2020**, *258*, 127333.
(54) Jablonowski, N. D.; Schaffer, A.; Burauel, P. Still present after all these years: persistence plus potential toxicity raise questions about the use of atrazine. *Environ. Sci. Pollut. Res.* **2011**, *18* (2), 328−331.
(55) Information on Biocides - ECHA. https://echa.europa.eu/information-on-chemicals/biocidal-active-substances (accessed Jan. 11, 2023).
(56) Igel, A. L.; Ekman, A. M. L.; Leck, C.; Tjernstrom, M.; Savre, J.; Sedlar, J. The Free Troposphere as a potential source of Arctic boundary layer aerosol particles. *Geophys. Res. Lett.* **2017**, *44* (13), 7053−7060.
(57) Lewis, K. A.; Tzilivakis, J.; Warner, D. J.; Green, A. An international database for pesticide risk assessments and management. *Hum. Ecol. Risk Assess.: Int. J.* **2016**, *22* (4), 1050−1064.

(58) Gotz, C. W.; Scheringer, M.; Macleod, M.; Roth, C. M.; Hungerbühler, K. Alternative approaches for modeling gas-particle partitioning of semivolatile organic chemicals: model development and comparison. *Environ. Sci. Technol.* **2007**, *41* (4), 1272−1278.
(59) Krejci, R.; Zieger, P. *Personal Communication*; University of Stockholm, 2023.
(60) Harner, T.; Bidleman, T. F. Octanol-air partition coefficient for describing particle/gas partitioning of aromatic compounds in urban air. *Environ. Sci. Technol.* **1998**, *32*, 1494−1502.
(61) Meyer, T.; Lei, Y. D.; Muradi, I.; Wania, F. Organic contaminant release from melting snow. 1. Influence of chemical partitioning. *Environ. Sci. Technol.* **2009**, *43* (3), 657−662.
(62) Atkinson, R. Kinetics and mechanisms of the gas-phase reactions of the hydroxyl radical with organic compounds under atmospheric conditions. *Chem. Rev.* **1985**, *85* (1), 69−201.
(63) Spivakovsky, C.M.; Logan, J. A.;Montzka, S. A.; Balkanski, Y. J.; Foreman-Fowler, M.; Jones, D. B. A.; Horowitz, L. W.; Fusco, A. C.; Brenninkmeijer, C. A. M.; Prather, M. J.; Wofsy, S. C.; McElroy, M. B. Three-Dimensional Climatological Distribution of Tropospheric OH: Update and evaluation. *J. Geophys. Res.: Atmos.* **2000**, *105* (D7), 8931−8980.
(64) Lammel, G. Effects of time-averaging climate parameters on predicted multicompartmental fate of pesticides and POPs. *Environ. Pollut.* **2004**, *128* (1−2), 291−302.
(65) Beyer, A.; Mackay, D.; Matthies, M.; Wania, F.; Webster, E. Assessing long-range transport potential of persistent organic pollutants. *Environ. Sci. Technol.* **2000**, *34* (4), 699−703.
(66) Bennett, D. H.; McKone, T. E.; Matthies, M.; Kastenberg, W. E. General formulation of characteristic travel distance for semivolatile organic chemicals in a multimedia environment. *Environ. Sci. Technol.* **1998**, *32* (24), 4023−4030.
(67) Mattei, C.;Wortham, H.; Quivet, E. Heterogeneous degradation of Pesticides by OH radicals in the atmosphere: influence of humidity and particle type on the kinetics. *Sci. Total Environ.* **2019**, *664*, 1084−1094.
(68) Smit, A. A. M. F. R.; van den Berg, F.; Leistra, M. *Estimation Method for the Volatilisation of Pesticides from Fallow Soil, Technical Report 2, Environmental Planning Bureau Series* DLO Winand Staring Centre: Wageningen, the Netherlands; 1997.
(69) Smit, A. A. M. F. R.; Leistra, M.; van den Berg, F. *Estimation Method for the Volatilisation of Pesticides from Plants, Technical Report 2, Environmental Planning Bureau Series*; DLO Winand: Wageningen, the
Netherlands, 1998.
(70) Nuyttens, D.; Devarrewaere, W.; Verboven, P.; Foque, D. Pesticide-laden dust emission and drift from treated seeds during seed drilling: a review. *Pest. Manag. Sci.* **2013**, *69* (5), 564−575.
(71) Das, S.; Hageman, K. J. Influence of adjuvants on pesticide soil-air partition coefficients: laboratory measurements and predicted effects on volatilization. *Environ. Sci. Technol.* **2020**, *54* (12), 7302-7308.
(72) Maggi, F.; Tang, F. H. M.; la Cecilia, D.; McBratney, A. PEST-CHEMGRIDS, global gridded maps of the top 20 crop-specific pesticide application rates from 2015 to 2025. Sci. Data **2019**, 6(1), 170.
(73) Hulin, M.; Leroux, C.; Mathieu, A.; Gouzy, A.; Berthet, A.; Boivin, A.; Bonicelli, B.; Chubilleau, C.; Hulin, A.; Leoz Garziandia, E.; Mamy, L.; Millet, M.; Pernot, P.; Quivet, E.; Scelo, A. L.; Merlo, M.; Ruelle, B.; Bedos, C. Monitoring of pesticides in ambient air: prioritization of substances. Sci. Total Environ. **2021**, 753, 141722.
(74) Jepson, P. C.; Murray, K.; Bach, O.; Bonilla, M. A.; Neumeister, L. Selection of pesticides to reduce human and environmental health risks: a global guideline and minimum pesticides list. The Lancet Planet. Health **2020**, 4(2), e56–e63.
(75) EU Pesticides Database - Active substances. https://ec.europa.eu/food/plant/pesticides/eu-pesticides-database/start/screen/active-substances (accessed Nov. 1st 2023).
(76) Lenoir, J. S.; McConnell, L. L.; Fellers, G. M.; Cahill, T. M.; Seiber, J. N. Summertime transport of current-use pesticides from California's central valley to the Sierra Nevada mountain range, USA. Environ. Toxicol. Chem. **1999**, 18(12), 2715–2722.
(77) Daly, G. L.; Lei, Y. D.; Teixeira, C.; Muir, D. C. G.; Wania, F. Pesticides in western Canadian mountain air and soil. Environ. Sci. Technol. **2007**, 41(17), 6020–6025

(78) Aston, L. S.; Seiber, J. N. Fate of Summertime Airborne organophosphate pesticide residues in the Sierra Nevada mountains. J. Environ. Qual. **1997**, 26(6), 1483–1492.
(79) Guida, Y. de S.; Meire, R. O.; Torres, J. P. M.; Malm, O. Air Contamination by legacy and current-use pesticides in brazilian mountains: an overview of national regulations by monitoring pollutant presence in pristine areas. Environmental Pollution **2018**, 242, 19–30.
